# Tuning the Properties of Rigidified Acyclic DEDPA^2–^ Derivatives for Application in PET Using Copper-64

**DOI:** 10.1021/acs.inorgchem.4c04050

**Published:** 2024-11-07

**Authors:** Daniel Torralba-Maldonado, Axia Marlin, Fátima Lucio-Martínez, Antía Freire-García, Jennifer Whetter, Isabel Brandariz, Emilia Iglesias, Paulo Pérez-Lourido, Rosa M. Ortuño, Eszter Boros, Ona Illa, David Esteban-Gómez, Carlos Platas-Iglesias

**Affiliations:** † Departament de Química, Universitat Autònoma de Barcelona, 08193, Cerdanyola del Vallès, Spain; ‡ Department of Chemistry, University of Wisconsin-Madison, Madison, Wisconsin 53706, United States; § Centro Interdisciplinar de Química e Bioloxía (CICA) and Departamento de Química, Facultade de Ciencias, Universidade da Coruña, Galicia, 15071 A Coruña, Spain; ∥ Departamento de Química Inorgánica, Facultad de Química, Universidade de Vigo, As Lagoas, Marcosende, 36310 Pontevedra, Spain

## Abstract

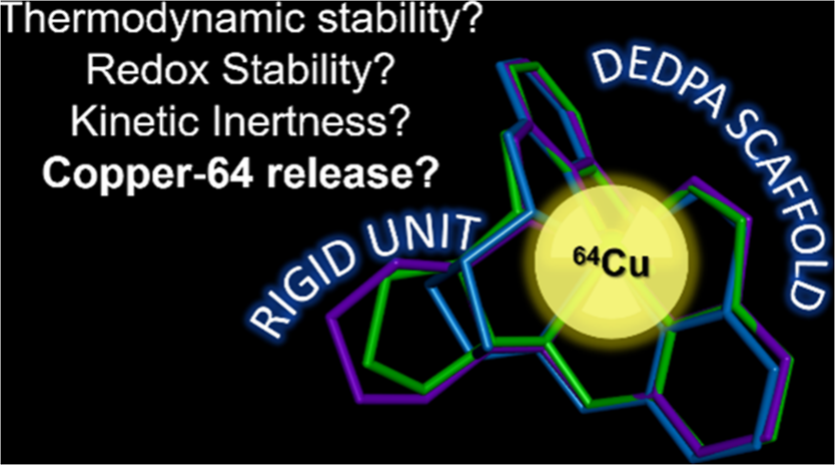

We present a detailed investigation of the coordination chemistry toward [^nat^Cu/^64^Cu]copper of a series of H_2_DEDPA derivatives (H_2_DEDPA = 6,6′-((ethane-1,2-diylbis(azanediyl))bis(methylene))dipicolinic acid) containing cyclohexyl (H_2_CHXDEDPA), cyclopentyl (H_2_CpDEDPA) or cyclobutyl (H_2_CBuDEDPA) spacers. Furthermore, we also developed a strategy that allowed the synthesis of a H_2_CBuDEDPA analogue containing an additional NHBoc group at the cyclobutyl ring, which can be used for conjugation to targeting units. The X-ray structures of the Cu(II) complexes evidence distorted octahedral coordination around the metal ion in all cases. Cyclic voltammetry experiments (0.15 M NaCl) evidence quasi-reversible reduction waves associated with the reduction of Cu(II) to Cu(I). The complexes show a high thermodynamic stability, with log *K*_CuL_ values of 25.11(1), 22.18(1) and 20.19(1) for the complexes of CHXDEDPA^2–^, CpDEDPA^2–^ and CBuDEDPA^2–^, respectively (25 °C, 1 M NaCl). Dissociation kinetics experiments reveal that both the spontaneous- and proton-assisted pathways operate at physiological pH. Quantitative labeling with ^64^CuCl_2_ was observed at 0.1 nmol for CHXDEDPA^2–^ and CpDEDPA^2–^, 0.025 nmol for CBuDEDPA^2–^ and 1 nmol for CBuDEDPA-NHBoc^2–^, with no significant differences observed at 15, 30, and 60 min. The radio-complexes are stable in PBS over a period of 24 h.

## Abstract 2

The nature of the rigid central spacer in acyclic chelators has a significant impact in the properties of their toward [^nat^Cu/^64^Cu]copper complexes

## Introduction

Among the radionuclides with interesting properties for positron emission tomography (PET) is copper-64, which decays with a half-life of *t*_1/2_ = 12.7 h through electron capture (43.9%) and β^–^ (38.5%) and β^+^ (17.6%) decays, and can be produced both using reactors and cyclotrons.^1,2^ The development of ^64^Cu-based radiopharmaceuticals requires the preparation of bifunctional chelators, which incorporate a metal ion binding site and a coupling function separated by a spacer. The coupling function is used to link the chelator to a targeting unit, which is responsible for taking the probe to the desired target in vivo.

**Chart 1:**
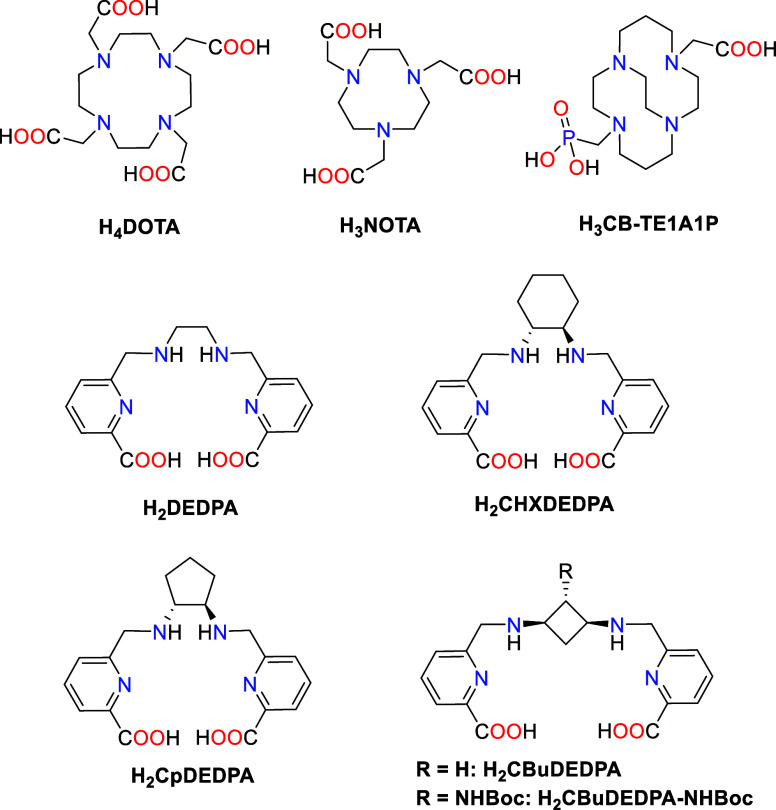
Structures of Ligands Discussed in the Present Work

In 2020, the Food and Drug Administration approved the first ^64^Cu-based radiopharmaceutical for clinical use, [^64^Cu]Cu-DOTATATE, which is based on a DOTA chelating unit and was shown to provide similar results to [^68^Ga]Ga-DOTATATE.^3^ Despite their widespread use, it is well-known that DOTA derivatives are not ideal chelators for Cu^2+^, with NOTA performing significantly better (Chart 1).^4−7^ The number of ^64^Cu-based radiopharmaceuticals entering clinical trials is growing rapidly,^8^ with current candidates being most commonly based on DOTA,^9^ NOTA,^10^ cyclam cross-bridge derivatives such as CB-TE1A1P^11^ and sarcophagine.^12,13^ However, the seek for new efficient chelators remains an active area of research.^14−23^ In particular, the biodistribution of the radioconjugate may be significantly affected by the nature of the bifunctional chelator if peptides are used as targeting units.^24^ Thus, it is important to expand the library of currently available chelators suitable for ^64^Cu-based radiopharmaceuticals.

The acyclic chelator H_2_DEDPA and the rigidified derivative H_2_CHXDEDPA^25^ were highlighted as very promising chelators for the development of ^68^Ga-based radiopharmaceuticals (Chart 1).^26,27^ Early studies performed on H_2_DEDPA derivatives evidenced poor stability in serum of the radiolabeled [^64^Cu(DEDPA)] complex,^28^ while the [^64^Cu(CHXDEDPA)] complex is 98% stable after 24 h in human serum at 37 °C.^29^ This striking different behavior highlights the beneficial effect that introducing a rigid cyclohexyl group has in the stability of the ^64^Cu-labeled complexes. A number of structural modifications were introduced to the H_2_DEDPA scaffold to obtain bifunctional derivatives^26^ or to introduce redox-active^29^ or fluorescent^30^ units. *N*-alkylation^31^ and functionalization of position 4 of the pyridyl rings^30^ of H_2_DEDPA were found to be detrimental for the radiolabeling efficiency and/or stability of the radiolabeled ^68^Ga-labeled complexes, with C-functionalization being the most promising strategy.^26^ The situation appears to be somewhat different for ^64^Cu-derivatives, with some studies demonstrating an increased stability of *N*-alkylated derivatives.^32,33^

Considering the very promising properties of H_2_DEDPA compounds for the development of ^64^Cu-based radiopharmaceuticals, we envisaged to expand the family of rigid derivatives and analyze their potential for ^64^Cu-based radiopharmaceutical development. Furthermore, we also aimed at developing the synthetic methodology required to prepare *C*-functionalized derivatives of H_2_DEDPA, gaining access to bifunctional ligands. Thus, we have modified the ethylene bridge of H_2_DEDPA by introducing rigid units to give the corresponding cyclopentyl (H_2_CpDEDPA) and cyclobutyl (H_2_CBuDEDPA) compounds. The coordination chemistry properties of these chelators were then compared with those of the cyclohexyl analogue H_2_CHXDEDPA (Chart 1). The H_2_CHXDEDPA ligand was reported in 2008 and studied for Ni(II), Zn(II), Cd(II) and Pb(II) complexation.^25,34^ The H_2_CBuDEDPA ligand was reported recently and used for Fe(III) complexation,^35^ while H_2_CpDEDPA is reported here for the first time. 1,2-Cyclobutanediamine-based ligands had been described before for the complexation of Gd(III) and Mn(II),^36,37^ demonstrating that the rigidity of the 4-membered carbocycle used as spacer enhanced the kinetic inertness of the resulting complexes.

## Results and Discussion

### Syntheses

The synthesis of the H_2_CpDEDPA ligand was achieved by reaction of commercially available *trans*-1,2-cyclopentanediamine and methyl 6-formylpyridine-2-carboxylate,^38^ followed by reduction of the intermediate Schiff-base with NaBH_4_. Hydrolysis of the methyl ester groups in 6 M HCl and purification using reverse-phase chromatography afforded the ligand in 48% yield.

**Scheme 1:**
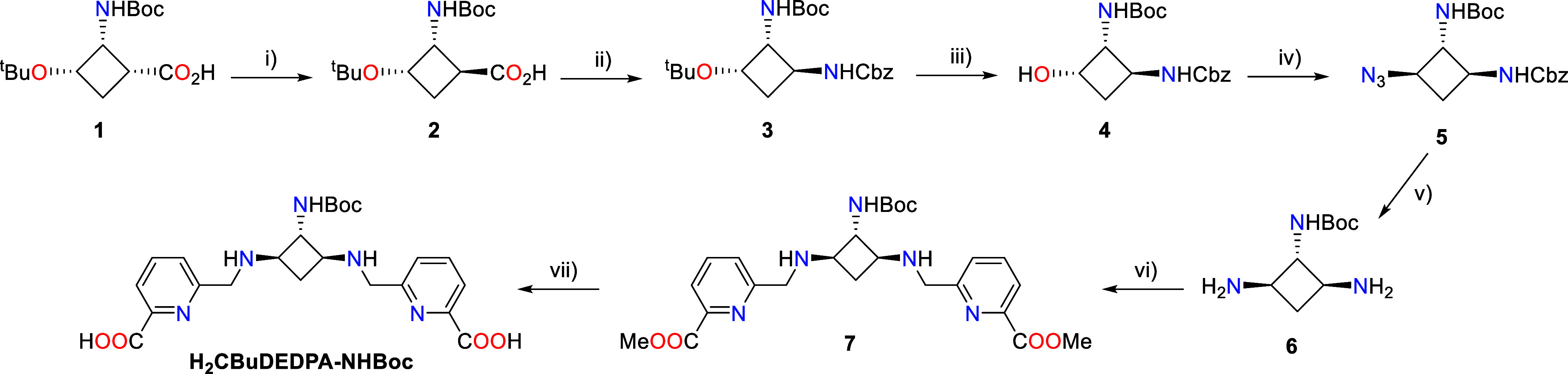
Synthesis of the Bifunctional precursor H_2_CBuDEDPA-NHBoc Reagents and conditions: (i) (a) Boc_2_O, pyridine, NH_4_HCO_3_, dioxane, 0 °C to rt, 4 h; (b) 6.25 M NaOH, MeOH, reflux, 18 h, 76%; (ii) (a) DPPA, Et_3_N, toluene, reflux, 2 h; (b) BnOH, 80 °C, 6 h, 81%; (iii) (a) TFA, 0 °C to rt, 3 h; (b) Boc_2_O, Et_3_N, THF, 0 °C to rt, 4 h, 87%; (iv) DPPA, PPh_3_, DIAD, THF, 0 °C to rt, 3 h, 79%; (v) H_2_, Pd/C, MeOH, rt, 16 h, Quantitative; (vi) (a) methyl 6-formylpicolinate, MeOH, 40 °C, 2.5 h; (b) NaBH_3_CN 0 °C to rt, 4 h, 50%.; (vii) LiOH, H_2_O:THF, rt, 3 h, 53%

The synthesis of the protected bifunctional ligand H_2_CBuDEDPA-NHBoc was achieved following the strategy presented in Scheme 1 (see Supporting Information for details). The synthesis started from previously reported trifunctionalized cyclobutane derivative **1**,^39^ which affords a protected alcohol and a carboxyl group as precursors of the *cis*-1,3-diamino system. To epimerize the α-position to the carboxyl group, compound **1** was converted temporarily into a mixed acid anhydride, which was subsequently hydrolyzed to give **2** as the major product in a 5:1 mixture together with starting material **1** (76% yield over the two steps). This mixture was subjected to a Curtius rearrangement using diphenylphosphoryl azide under reflux and the resulting isocyanate was reacted with benzyl alcohol to yield dicarbamate **3** in 81% yield after purification. Deprotection of the hydroxyl group in **3** was carried out using neat TFA. This reaction also implied the acidolysis of the *tert*-butylcarbamate. The resulting amine was reprotected using di-*tert*-butyl dicarbonate, **4** being obtained in 87% yield over the two steps. After that, a Mitsunobu reaction was carried out in order to install an azide in place of the hydroxyl group with inversion of configuration, which afforded **5** in 79% yield. Subsequently, Pd-catalyzed hydrogenolysis of the benzyl carbamate and reduction of the azide group led to triamine **6** in quantitative yield. Compound **6** was submitted to double reductive amination with methyl 6-formylpicolinate,^38^ rendering orthogonally protected precursor **7** in 50% yield. The saponification of the methyl ester groups with LiOH afforded the desired bifunctional *all*-*trans*-triamine ligand H_2_CBuDEDPA-NHBoc, which contains a spacer bearing a protected amino group suitable for conjugation. The ligand was obtained in a 7-step sequence in 11% overall yield.

The Cu(II) complexes of CHXDEDPA^2–^, CpDEDPA^2–^ and CBuDEDPA^2–^ were synthesized by reaction of the corresponding ligand with Cu(OTf)_2_ in aqueous solution at pH ∼7 and isolated in ∼50% yields after purification through reverse-phase chromatography using a C18AQ column (see Supporting Information for details). The UV–vis absorption spectra of the three complexes are very similar (Figure S27, Supporting Information), showing d–d absorption bands at 720 ([Cu(CHXDEDPA)]), 732 ([Cu(CpDEDPA)]) and 728 nm ([Cu(CBuDEDPA)]) (ε ∼ 75 M^–1^ cm^–1^). These absorption data are very similar to those reported for [Cu(OCTAPA)]^2–^ and [Cu(DEDPA)], which have distorted N_4_O_2_ octahedral coordination.^40^ This indicates that the nature of the spacer has little impact on the structures of these complexes in solution.

### X-ray Structures

**Figure 1:**
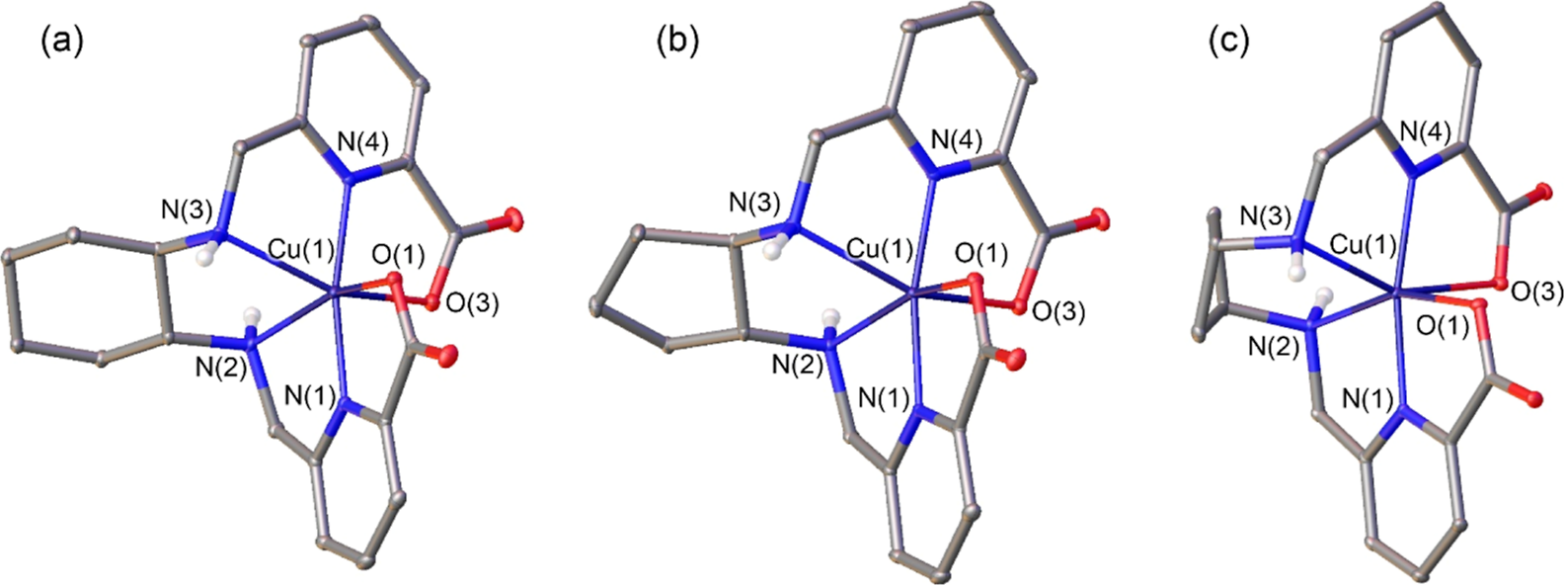
X-ray structures of: (a) [Cu(CHXDEDPA)]·(CH_3_)_2_CO·H_2_O, (b) [Cu(CpDEDPA)]·4H_2_O and (c) [Cu(CBuDEDPA)] with ellipsoids plotted at the 30% probability level. Solvent molecules and H atoms bonded to C atoms are omitted for simplicity.

Single crystals were obtained by slow diffusion of acetone into aqueous solutions of the Cu(II) complexes. Crystals contain the charge neutral complexes and solvent molecules (water and/or acetone). The bond distances of the Cu(II) coordination sphere are listed in Table 1, while views of the structures of the complexes are shown in Figure 1 (see Table S3 for bond angles). The metal ions are coordinated to the six donor atoms of the ligand, which wraps around the metal ion to offer a distorted octahedral coordination environment. However, the bond angles of the metal coordination sphere evidence a strong distortion of the octahedral coordination, with *trans* angles in the range of ∼149–169°. Furthermore, the bond distances of the metal coordination environment involving the same type of donor atom differ significantly, which is characteristic of six-coordinate Cu(II) complexes with Jahn–Teller distortion.^41,42^ This effect is particularly pronounced for the Cu(1)–O(1) and Cu(1)–O(3) distances, which differ by 0.181, 0.224, and 0.179 Å for [Cu(CHXDEDPA)], [Cu(CpDEDPA)] and [Cu(CBuDEDPA)], respectively. Similar distortions of the metal coordination sphere were observed previously for [Cu(DEDPA)] and *N*-alkylated derivatives of H_2_CHXDEDPA.^28,29^

**Table 1:** Bond Distances of the Cu(II) Coordination Environments Determined by Single-Crystal X-ray Measurements and Shape Measures for Octahedral and Trigonal Prismatic Coordination^a^

	CHXDEDPA^2–^	CpDEDPA^2–^	CBuDEDPA^2–^
Cu(1)–N(1)	1.9283(17)	1.916(2)	1.944(2)
Cu(1)–N(2)	2.1429(19)	2.122(2)	2.156(2)
Cu(1)–N(3)	2.2651(19)	2.303(3)	2.258(2)
Cu(1)–N(4)	1.9748(17)	1.962(2)	1.993(2)
Cu(1)–O(1)	2.0961(15)	2.079(2)	2.1102(18)
Cu(1)–O(3)	2.2771(15)	2.294(2)	2.2887(18)
S(OC-6)^a^	4.786	4.629	3.541
S(TPR-6)^a^	6.243	6.375	9.581

a Shape measures for octahedral, S(OC-6), and trigonal prismatic S(TPR-6), coordination.

Shape measurements were conducted to assess the degree of distortion of the metal coordination environment.^43−45^ The [Cu(CHXDEDPA)] and [Cu(CpDEDPA)] complexes are characterized by similar Shape measures for an octahedral coordination [S(OC-6), Table 1], while a trigonal prismatic coordination affords higher Shape measures [S(TPR-6), Table 1]. This indicates that the coordination environment is best described as octahedral, though rather distorted (an ideal octahedral coordination is characterized by S(OC-6) = 0). Shape measures indicate that the octahedral coordination in [Cu(CBuDEDPA)] is less distorted than in [Cu(CHXDEDPA)] and [Cu(CpDEDPA)]. The Shape measures obtained for this series of structurally related compounds differ significantly from those characterizing the minimal distortion pathway between an octahedron and a trigonal prism (Figure S23, Supporting Information),^46^ which reflects the constraints on the metal coordination environment imposed by these rigid ligands.

### Cyclic Voltammetry

Cyclic voltammetry experiments were carried out to assess the stability of the Cu(II) complexes toward reduction. A recent study demonstrated that the redox potential of Cu(II) complexes must be shifted out of the window of bioreducing agents like ascorbate, as even very inert Cu(II) complexes can dissociate quickly upon reduction to Cu(I).^47^ The threshold of bioreducing agents was estimated to be −0.4 V versus NHE.^48^

**Figure 2:**
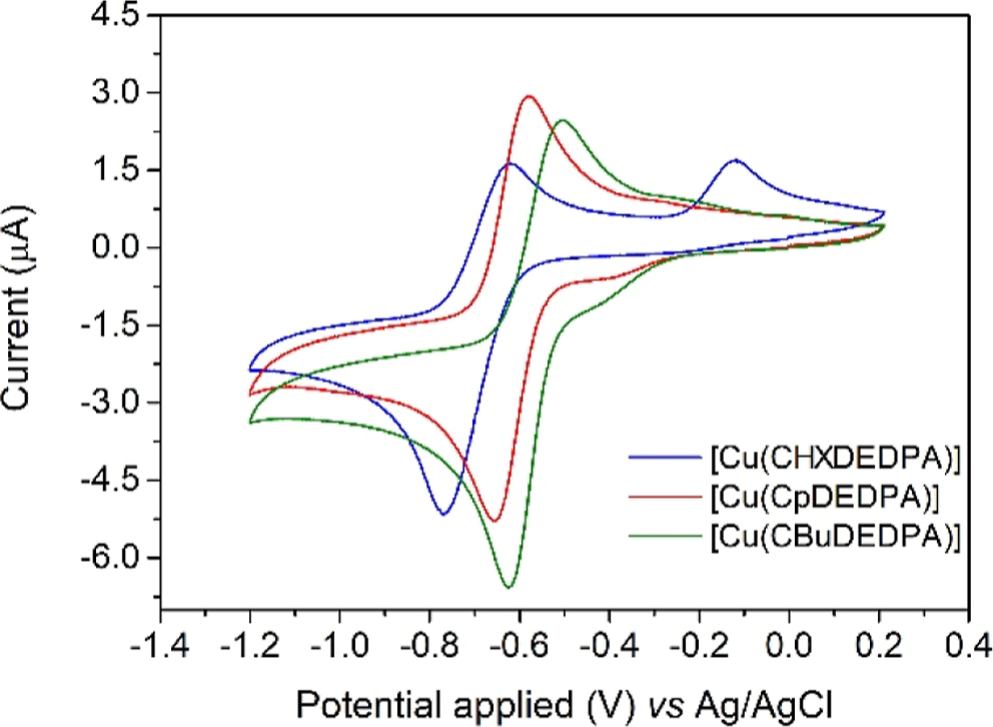
Cyclic voltammograms recorded from aqueous solutions of the Cu(II) complexes (0.15 M NaCl, scan rate 50 mV/s). Conditions: [Cu(CHXDEDPA)], 1.4 mM, pH 6.6; [Cu(CpDEDPA)], 1.3 mM, pH 6.9; [Cu(CBuDEDPA)], 1.3 mM, pH 5.0.

The cyclic voltammograms (50 mV/s) recorded from aqueous solutions of the complexes in 0.15 M NaCl (vs Ag/AgCl) display quasi-reversible features with half-wave potentials *E*_1/2_ = −695 mV (Δ*E*_p_ = 149 mV), *E*_1/2_ = −618 mV (Δ*E*_p_ = 73 mV) and *E*_1/2_ = −565 mV (Δ*E*_p_ = 120 mV) for [Cu(CHXDEDPA)], [Cu(CpDEDPA)] and [Cu(CBuDEDPA)], respectively (Figure 2). For all three complexes Δ*E*_p_ increases upon increasing the scan rate, in line with electrochemically quasi-reversible processes. The peak current varies linearly with the square-root of the scan rate, which suggests that the electrochemical processes are diffusion-controlled (Figures S24–S26, Supporting Information).^49^ The cyclic voltammograms show a second irreversible oxidation wave that is more prominent at low scan rates, most likely arising from the structural reorganization of the reduced Cu(I) species. This irreversible oxidation wave is particularly prominent at −160 mV for [Cu(CHXDEDPA)] (Figure 2). Of note, the cyclic voltammogram reported for [Cu(DEDPA)] shows an irreversible reduction peak at −1.12 V (vs Ag/AgCl),^28^ as well as an oxidative stripping peak of Cu(0) to Cu(II) around 0.0 V^50^ that indicates dissociation of the Cu(I) complex within the time scale of the experiment. This is indicative of an increased stability upon reduction of the rigid derivatives described here with respect to the parent [Cu(DEDPA)] complex.

The reduction potential of [Cu(CHXDEDPA)] is clearly out of the threshold of common bioreductants (*E*_1/2_ = −695 mV versus Ag/AgCl corresponds to −475 mV versus NHE).^51^ The reduction potentials determined by cyclic voltammetry for [Cu(CpDEDPA)] and [Cu(CBuDEDPA)] are right on the edge of this threshold.

The *E*_1/2_ values obtained for this series of complexes indicate that the ability to stabilize Cu(I) increases following the trend CBuDEDPA^2–^ > CpDEDPA^2–^ > CHXDEDPA^2–^. This can be correlated with the ability of the ligand to provide an octahedral coordination with Jahn–Teller distortion. The Jahn–Teller distortion provides an additional stability to six-coordinate Cu(II) complexes,^52^ a contribution that is evidenced in the Irving-Williams order.^53^ A more negative *E*_1/2_ value is therefore expected for complexes having a larger shape measure for octahedral distortion (S(OC-6), Table 1), due to a limited stability enhancement due to the Jahn–Teller effect.

### Thermodynamic Stability

The protonation constants of the DEDPA^2–^ derivatives investigated here were determined using potentiometric titrations. The Cu(II) complexes of these ligands dissociate at rather low pH values, which prevented complex stability constant determination using direct methods and an ionic strength of *I* = 0.15 M NaCl. Thus, we determined the protonation constants of CHXDEDPA^2–^ using *I* = 1.0 M NaCl as the ionic strength. The protonation constants determined here using *I* = 1.0 M NaCl compare well with those reported in 0.15 M NaCl^27^ and 0.1 M (Me_4_N)(NO_3_),^25^ which indicates that the different nature and concentration of the background electrolyte does not have a significant impact on the protonation constants (Table 2).

**Table 2:** Ligand Protonation Constants and Stability Constants of the Cu(II) Complexes Determined Using Potentiometric and Spectrophotometric Titrations (1 M NaCl, 25 °C)

	CHXDEDPA^2–^	CpDEDPA^2–^	CBuDEDPA^2–^	DEDPA^2–^c
*I*	1 M NaCl	1 M NaCl	1 M NaCl	1 M NaCl
log *K*_1_^H^	9.41(1)/9.23^a^/9.13^b^	9.05(1)	8.95(1)	9.00^c^/8.69^b^
log *K*_2_^H^	6.45(1)/6.47^a^/6.44^b^	6.51(2)	6.87(1)	6.30^c^/6.18^b^
log *K*_3_^H^	3.35(2)/2.99^a^/3.25^b^	3.28(3)	3.32(2)	3.06^c^/3.08^b^
log *K*_4_^H^	2.48(2)/2.40^a^/2.40^b^	2.51(4)	2.52(3)	2.59^c^/2.33^b^
Σ log *K*_i_^H^ (*i* = 1–4)	21.69/21.09^a^/21.22^b^	22.18	20.19	20.95^c^
log *K*_CuL_	25.11(1)	22.18(1)	20.19(1)	19.16^d^
pCu^e^	24.0	21.4	19.5	18.5

a Data in 0.15 M NaCl from ref (27).

b Data in 0.1 M (Me_4_N)(NO_3_) from ref (25).

c Data in 0.15 M NaCl from ref (26).

d Data in 0.15 M NaCl from ref (28).

e Defined as–log [Cu(II)]_free_, for [L]_tot_ = 10 μM and [Cu(II)]_tot_ = 1 μM.

The first and second protonation constants (log *K*_1_^H^ and log *K*_2_^H^) correspond to the protonation of the amine N atoms of the ligand. The value of log *K*_1_^H^ increases slightly on replacing the central ethyl group of DEDPA^2–^ by a cyclohexyl ring. A similar effect was observed previously for the first protonation constant of EDTA^4–^ and the cyclohexyl derivative CDTA^4–^.^54^ This can be attributed, at least in part, to the electron-donating effect of the ring.^55^ The value of log *K*_1_^H^ decreases following the order CHXDEDPA^2–^ > CpDEDPA^2–^ > CBuDEDPA^2–^, likely reflecting a decreased cooperation between the amine N atoms during the first protonation process.^56^ The value of log *K*_2_^H^ determined for CBuDEDPA^2–^ is the highest among this series of closely related derivatives, which likely reflects a decreased electrostatic repulsion in the deprotonated form due to the large distance between the amine N atoms, which are placed at positions 1 and 3 of the cyclobutyl unit.

The stability constants of the Cu(II) complexes were determined using spectrophotometric titrations, as complex dissociation occurs in a pH range that is not appropriate for direct potentiometric titrations (<2.0, see Figures S28–S30, Supporting Information). The absorption spectra of the complexes display the characteristic absorption due to the picolinate chromophore at 270 nm. The intensity of this band decreases upon lowering the pH due to complex dissociation, eventually yielding the spectrum of the LH_4_^2+^ species. To aid data analysis, the absorption spectrum of LH_4_^2+^ was obtained independently and provided to the fitting program.

**Figure 3:**
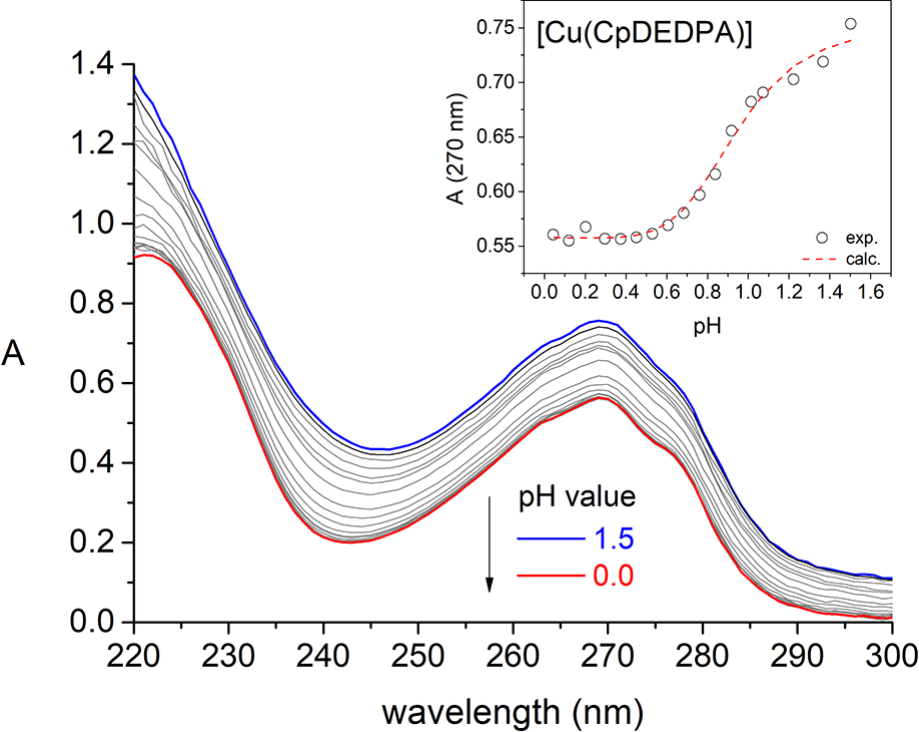
Spectrophotometric titration of [Cu(CpDEDPA)] (6.36 × 10^–5^, *I* = 1 M NaCl) with pH. The inset shows the experimental absorbance values at 270 nm and the dashed line the fit of the data for stability constant determination.

The fits of the spectrophotometric data (Figure 3, see also Figures S31–S32, Supporting Information) afford the stability constants shown in Table 2. The complex with CHXDEDPA^2–^ displays the highest thermodynamic stability constant among this series of complexes, with a remarkably high log *K*_CuL_ = 25.11. This represents an increase in complex stability of 6 orders of magnitude compared with DEDPA^2–^. The stability constants of the Cu(II) complexes with these ligands follow the trend CHXDEDPA^2–^ > CpDEDPA^2–^ > CBuDEDPA^2–^, the latter being still 1 order of magnitude higher than that reported for DEDPA^2–^.^28^ Thus, modification of the ligand scaffold by introducing a rigid spacer has a beneficial impact in terms of complex stability, an effect that is particularly pronounced for the cyclohexyl derivative CHXDEDPA^2–^.

The stability of metal complexes for medical applications at physiological pH is generally assessed by their pM (pCu) values, which are often defined as–log [Cu(II)]_free_ for a total metal concentration of 1 μM and a total ligand concentration of 10 μM.^57^ The pCu values calculated under these conditions follow closely the trend observed for log *K*_CuL_ (Table 2). This is not surprising, given that DEDPA^2–^ and the three derivatives described here display similar basicities, as indicated by the sum of log *K*_i_^H^ values determined for each ligand (Table 2).

The stability constants and pCu values determined here are comparable to those of complexes with ligands commonly used as ^64^Cu chelators such as DOTA^4–^ (pCu = 17.6, 0.1 M KCl),^58^ NOTA^3–^ (pCu = 18.4, 1.0 M Na(ClO_4_))^59,60^ or bispa^–^ (pCu = 19.3, 0.1 M KNO_3_).^61^ Cross-bridge cyclam derivatives display very high stability constants (log *K*_CuL_ = 27.1 for CB-cyclam), though radiolabeling often requires high temperatures.^62^

### Dissociation Kinetics

**Figure 4:**
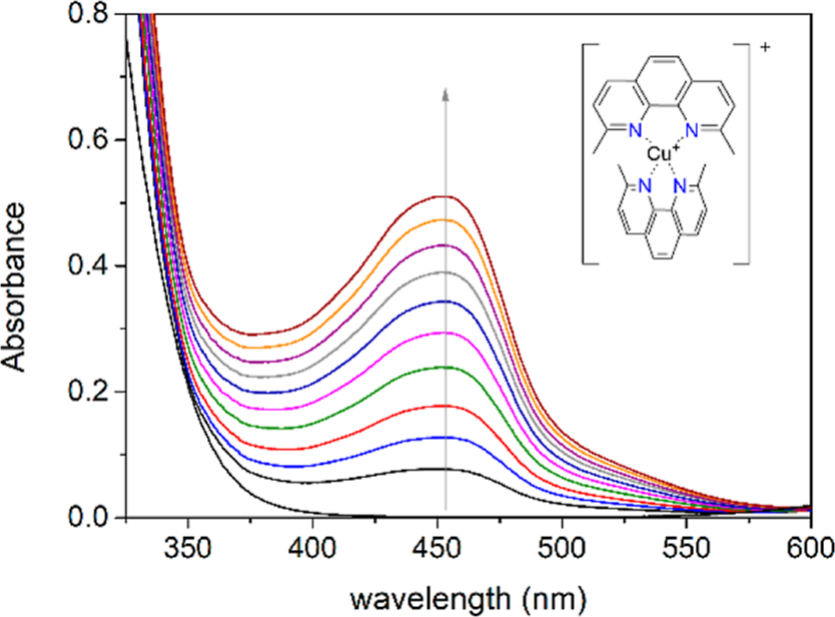
Spectral variations observed for a solution of [Cu(CBuDEDPA)] = 91 μM, [NC] = 0.24 mM, [AA] = 0.27 mM, [buffer] = 0.18 M. pH 6.7. Spectra were recorded every 6 min.

**Figure 5:**
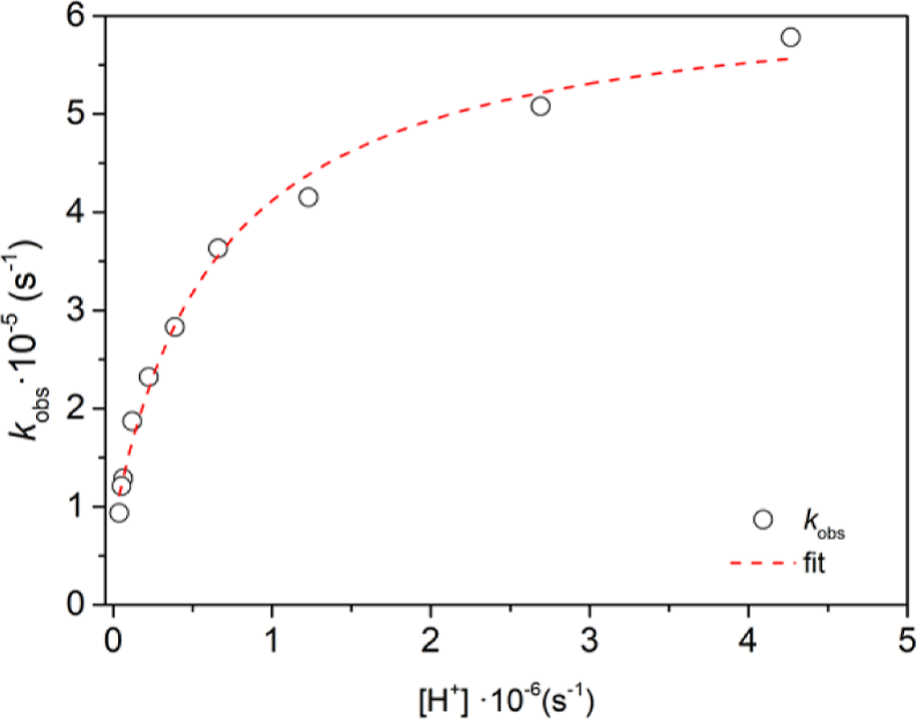
Dependence of the observed dissociation rate constants with proton concentration. Conditions: Spectral variations observed for a solution of [Cu(CBuDEDPA)] = 91 μM, [NC] = 0.24 mM, [AA] = 5.4 mM, [buffer] = 0.122 M.

The kinetic inertness of the radio-complexes is a key property for any radiopharmaceutical candidate. In the particular case of PET agents, the release of the radioisotope decreases the uptake of the probe in the target tissue and increases background noise. Often the inertness of the complex is tested in vitro by studying the acid-catalyzed dissociation under harsh acidic conditions, using acid concentrations of 1.0–6.0 M.^15,63−65^ However, these conditions are far away from those found in vivo. We recently proposed an alternative method to assess dissociation kinetics of Cu(II) complexes in a pH range relatively close to physiological conditions.^47^ In this approach, the dissociation reaction is triggered by the presence of ascorbate (AA) using neocuproine (NC) as a scavenger. Ascorbate reduces any free Cu(II) present in the solution to Cu(I), which forms a very stable complex with NC with a characteristic absorption band at 450 nm.^66^ The [Cu(CHXDEDPA)] complex does not dissociate in the presence of ascorbate at pH 6.7 over the course of 3 h (Figure S33, Supporting Information). Thus, we investigated the dissociation of the representative [Cu(CBuDEDPA)] complex in the pH range of 5.4–7.5 to gain information on the pathways that can potentially lead to complex dissociation under physiological conditions. These experiments were conducted using phosphate buffer and a large excess of both NC and ascorbate to ensure pseudo-first-order conditions (see details in Figure 4 and 5).

The observed dissociation rate constants *k*_obs_ do not vary with ascorbate concentration within experimental error (Table S4 and Figures S34–S35, Supporting Information). However, they increase with proton concentration, showing a saturation profile that indicates the formation of a protonated complex at the beginning of the reaction (Figure S36 and Table S5, Supporting Information). Thus, the observed first-order rate constants were fitted to eq 1
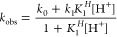
1

Here, *k*_0_ represents the rate constant for the spontaneous dissociation, while *k*_1_ is the rate constant characterizing the proton-assisted dissociation and *K*_1_^H^ is the protonation constant. The least-squares fit of the data affords *k*_0_ = (8.2 ± 1.3) × 10^–6^ s^–1^, *k*_1_ = (6.3 ± 0.2) × 10^–6^ s^–1^ and *K*_1_^H^ = (1.5 ± 0.2) × 10^6^ M^–1^. This kinetic data provide some unexpected results. First, this protonation constant corresponds to a p*K*_a_ of 6.2 that cannot be associated with any protonation constant of the octahedral complex. This p*K*_a_ value is actually only compatible with the protonation of an uncoordinated amino group. Thus, the kinetic data suggest that the proton-assisted dissociation pathway involves a kinetically active species in which amine N atoms are not directly coordinated to the metal. Second, the spontaneous dissociation pathway provides a significant contribution to the overall dissociation of the complex in the investigated pH range. However, at pH 7.4 the product *k*_1_*K*_1_^H^[H^+^] is 1 order of magnitude smaller than *k*_0_, and thus the spontaneous dissociation mechanism becomes dominant. In spite of this, the [Cu(CBuDEDPA)] complex is remarkably inert considering its acyclic nature, with a half-life at pH 7.4 of 23.7 h. Kinetic experiments performed in concentrated acid solutions are unlikely to provide information on the spontaneous pathway, which is likely negligible under those conditions.

### ^64^Cu Radiolabeling Experiments

**Figure 6:**
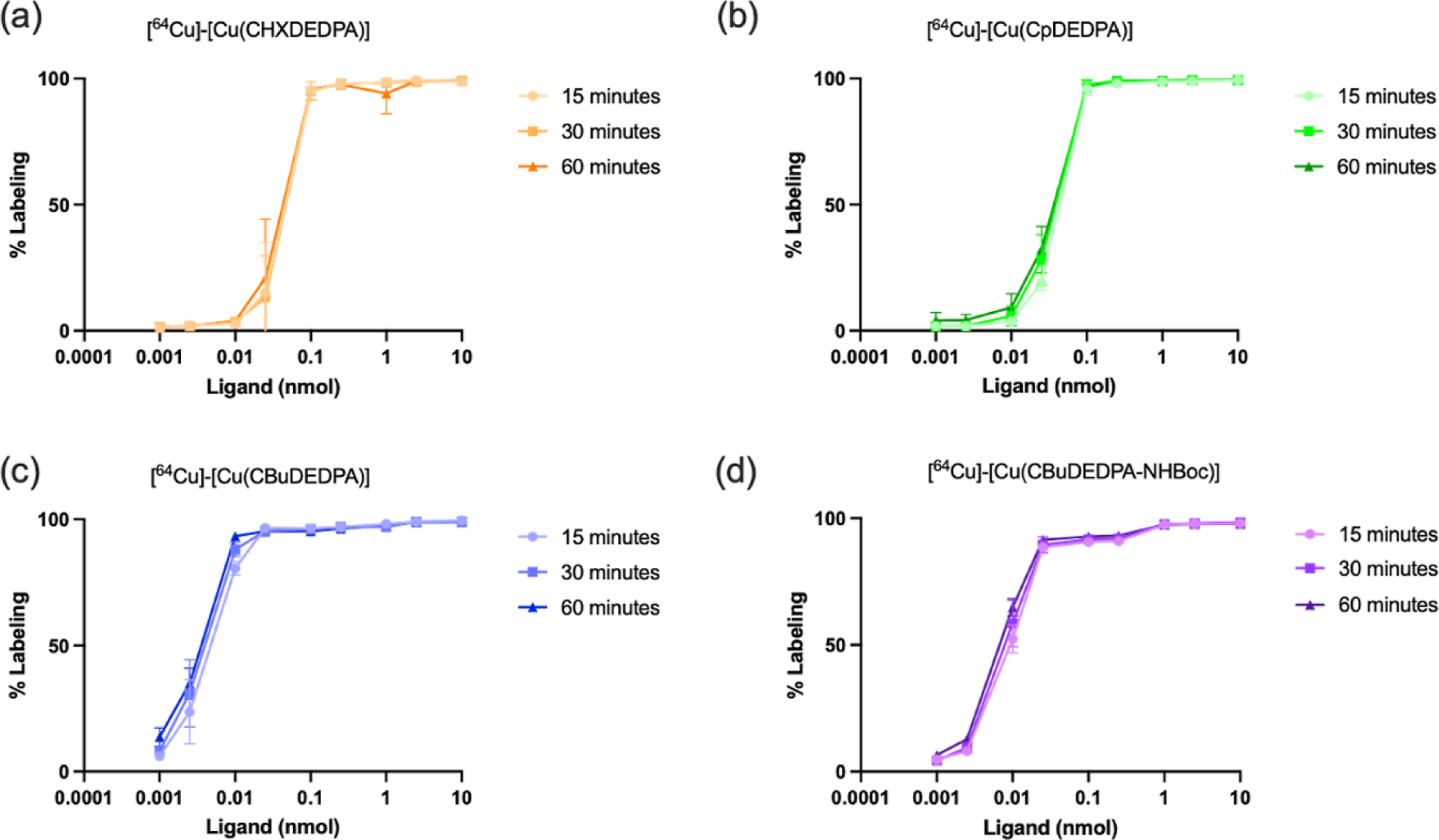
AMA determination curves for all chelator tested, with reaction yields analyzed at 3 time points, with *n* = 3 per data point. Activity per sample was 50–90 μCi with a total volume of 116 μL in 0.5 M ammonium acetate buffer pH 5.5.

**Figure 7:**
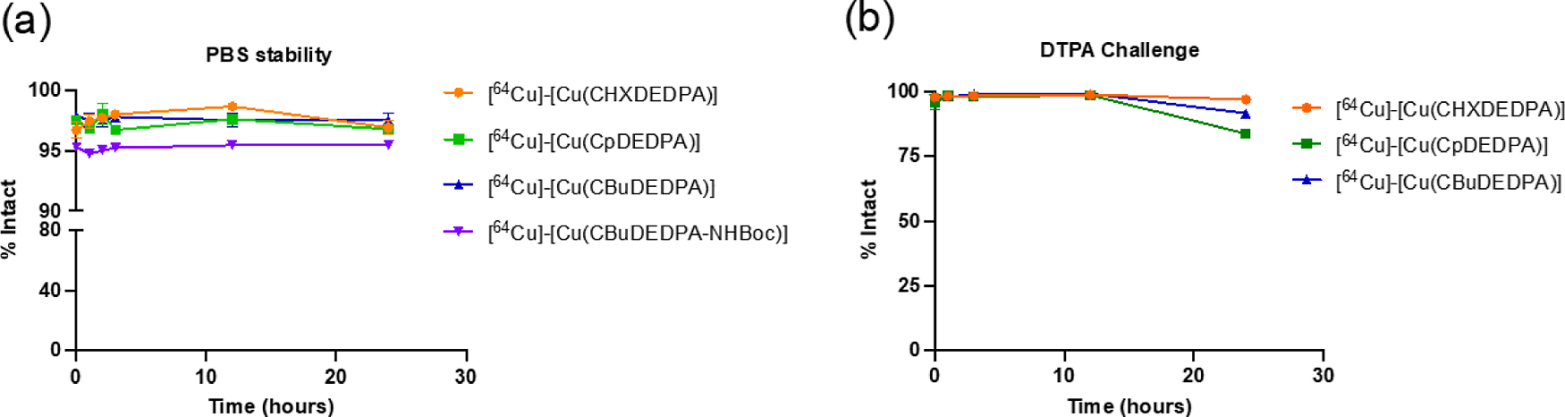
PBS stability (a) and DTPA challenge (b) of the ^64^Cu-complexes. Ligand = 1 nmol, DTPA = 100 nmol, *n* = 3.

The ability of the DEDPA-based ligands to coordinate ^64^Cu was determined by probing radiochelation at 25 °C in 0.5 M ammonium acetate buffer pH 5.5 with 50–90 μCi of ^64^CuCl_2_. Labeling efficiency was quantified via radio-TLC (Figure S37, Supporting Information). The Apparent Molar Activity (AMA) for each chelator at 15, 30, and 60 min was determined and is summarized in Table S6 (Supporting Information). Figure 6 shows the results of concentration dependent radiolabeling for all tested ligands. CBuDEDPA^2–^ produced quantitative radiolabeling at an AMA of 4.939 Ci·μmol^–1^ (Figure 6c). The CHXDEDPA^2–^ and CpDEDPA^2–^ chelators produced AMA values of 0.631 Ci·μmol^–1^ and 0.565 Ci·μmol^–1^ respectively (Figure 6a and 6b). Complexation experiments of CBuDEDPA-NHBoc^2–^ (Figure 6d) with ^64^Cu produced a specific molar activity of 3.563 Ci μmol^–1^, thus remaining at the same order of magnitude as the parent ligand structure. Quantitative labeling was observed at 0.1 nmol for CHXDEDPA^2–^ and CpDEDPA^2–^, 0.025 nmol for CBuDEDPA^2–^ and 1 nmol for CBuDEDPA-NHBoc^2–^ with no significant difference between 15, 30, and 60 min. The inertness of the formulated ^64^Cu complexes was evaluated in PBS, evidencing no significant decomplexation of the radio-complexes over 24 h (Table S7, Supporting Information). In addition, the ^64^Cu complex integrity was investigated using 100× excess of diethylenetriaminepentaacetic acid (DTPA). Transchelation was monitored by TLC over the course of 24 h (Figure 7, see also Table S8, Supporting Information). The inertness of CpDEDPA^2–^ is lower than that of CHXDEDPA^2–^ and CBuDEDPA^2–^ with 83.7 ± 1.5% intact at 24 h versus 96.9 ± 0.6% and 91.6 ± 1.8%, respectively. However, quantitative transchelation was observed for CBuDEDPA-NHBoc^2–^ at the 10 min time point, indicating that the presence of a bulky substituent at position 2 of the cyclobutyl ring has a negative impact in the stability of the radio-complex.

## Conclusions

We have investigated the coordination chemistry of a series of DEDPA^2–^ derivatives with [^nat^Cu/^64^Cu]copper by changing the nature of the central spacer. The analysis of the X-ray structures evidences that the nature of the spacer has a relatively minor impact in the metal coordination environment, although this subtle differences have some effect on the ability of the chelator to stabilize Cu(I). All three chelators containing rigid spacers form Cu(II) complexes with higher thermodynamic stability than the parent DEDPA^2–^ ligand, with stability following the sequence CHXDEDPA^2–^ > CpDEDPA^2–^> CBuDEDPA^2–^. The dissociation kinetics of the [Cu(CBuDEDPA)] complex were investigated close to physiological pH. This methodology allowed to identify both the spontaneous and proton-assisted pathways as responsible for the dissociation of the complex close to neutral pH. In contrast, dissociation experiments using high acid concentrations are unlikely to afford information on the spontaneous dissociation mechanism. The three chelators can be labeled with copper-64 at very low concentrations, with CBuDEDPA^2–^ providing the best radiolabeling efficiency. Furthermore, the radio-complexes of CHXDEDPA^2–^ and CBuDEDPA^2–^ show comparable stabilities in PBS and in the presence of excess DTPA as a competitor. This prompted us to develop a bifunctional analogue of CBuDEDPA^2–^containing an NHBoc group at the cyclobutyl ring. However, while this structural modification had a minor impact in the radiolabeling efficiency, it is clearly detrimental in terms of radio-complex stability. We are currently developing alternative strategies toward bifunctional derivatives of this family of acyclic chelators, among which CHXDEDPA^2–^ displays the most promising properties in terms of thermodynamic stability and kinetic inertness, as judged by the DTPA competition experiments.

## Experimental Section

### General

Solvents and reagents were purchased from commercial sources and were directly used without further purification, unless otherwise stated. Anhydrous dichloromethane was freshly distilled when needed under a nitrogen atmosphere from calcium chloride. Anhydrous toluene was freshly distilled from sodium/benzophenone. TLC on silica gel-coated aluminum plates was performed in the systems indicated for each product in their description. The compounds were visualized by exposure to UV light at 254 nm, dipping in a basic potassium permanganate solution or in an acid vanillin solution. Flash chromatography purifications were carried out on silica gel (200–400 mesh) or on basic alumina (0.06–0.2 mm) using the solvent system specified for each product in the Supporting Information. Melting points were recorded on a Reicher Kofler block and values are uncorrected. IR spectra were obtained from samples in neat form with a BRUKER Alpha II spectrophotometer with an ATR (Attenuated Total Reflectance) accessory. Medium performance liquid chromatography was performed in a Puriflash XS 420 InterChim Chromatographer equipped with a UV-DAD detector in reverse phase, using a 20 g BGB Aquarius C18AQ reversed-phase column (100 Å, spherical, 15 μm). The experimental conditions are described below for each case. Preparative high performance liquid chromatography (HPLC) was performed using an Agilent 1260 Infinity II instrument equipped with an UV Variable Wavelenghth Detector, in manual injection and collection mode, using an Agilent InfinityLab ZORBAX 5 Eclipse Plus C18 (5 μm, 21.2 × 250 mm) and 10 mM ammonium acetate aqueous solution (phase A) and CH_3_CN with 10% of phase A (phase B) as the mobile phases, operating at a flow rate of 20 mL/min. High-resolution electrospray-ionization time-of-flight (ESI-TOF) mass spectra were recorded in positive mode using a LTQ-Orbitrap Discovery Mass Spectrometer coupled to a Thermo Accela HPLC. Elemental analyses were obtained using a ThermoQuest Flash EA 1112 elemental analyzer. Aqueous solutions were lyophilized using a Biobase BK-FD10 Series apparatus. ^1^H and ^13^C NMR spectra of the ligands and their precursors were recorded on Bruker 300 MHz Ascend or Bruker 300 MHz AVANCE III, Bruker 400 MHz AVANCE III or Bruker AVANCE 500 spectrometers.

^64^CuCl_2_ was obtained from the University of Wisconsin Madison in a 0.1 M HCl solution. Radio-HPLC analysis was carried out using a Shimadzu HPLC-20AR chromatographer equipped with a binary gradient, pump, UV–vis detector, autoinjector, and Laura radiodetector on a Phenomenex Gemini C18 column (3 μm, 3 × 150 mm). Method A: (A) 0.1% TFA in water and (B) 0.1% TFA in CH_3_CN with a flow rate of 0.8 mL min^–1^ (Gradient. 0–2 min: 5% B. 2–24 min: 5–95%B. 24–26 min: 95%B. 26–28 min: 95–5%B. 28–30 min: 5%B). Method B: (A) 10 mM NH_4_OAc pH 7 (B) CH_3_CN with a flow rate of 0.8 mL min^–1^ (Gradient. 0–2 min: 5% B. 2–24 min: 5–95%B. 24–28 min: 95% B. 28–30 min: 5% B) and UV detection at 220 and 254 nm. HPLC traces are shown in Figures S38–S39, Supporting Information.

### Electrochemical Measurements

Cyclic voltammetry experiments were conducted using a three-electrode setup with an Autolab PGSTAT302 M potentiostat/galvanostat. The working electrode was a glassy carbon disk (Metrohm 61204600), which was polished with α-Al_2_O_3_ (0.3 μm) and rinsed with distilled water prior to each measurement. An Ag/AgCl reference electrode filled with 3 M KCl (Metrohm 6.0726.100) served as the reference electrode, while a platinum wire was used as the counter electrode. Prior to each measurement, the complex solutions containing 0.15 M NaCl were deoxygenated by bubbling nitrogen through them. Conditions: [Cu(CHXDEDPA)], 1.4 mM, pH 6.6; [Cu(CpDEDPA)], 1.3 mM, pH 6.9; [Cu(CBuDEDPA)], 1.3 mM, pH 5.0.

### Thermodynamic Studies

Ligand protonation constants and stability constants of the complexes were determined using potentiometric and spectrophotometric titrations using HYPERQUAD.^67^ All experimental data were collected at 25 °C using NaCl as inert electrolyte to keep constant the ionic strength (*I* = 1 M). Potentiometric titrations were conducted in a dual-wall thermostated cell with recirculating water to maintain a consistent temperature. To prevent CO_2_ absorption, nitrogen was bubbled over the surface of the solution, and magnetic stirring was used to ensure thorough mixing. A Crison microBu 2030 automatic buret was employed to add the titrant, and the electromotive force (emf) was measured with a Crison micropH 2000 pH meter, which was connected to a Radiometer pHG211 glass electrode and a Radiometer REF201 reference electrode. Initially, 10 mL of a 1.5–2.0 mM chelator solution was added to the cell, and the pH was adjusted to 11 with NaOH. This solution was then titrated with a standard HCl solution to determine all protonation constant values within a single experiment. All potentiometric titrations were performed in duplicate. Due to the low pH at which dissociation occurs, the stability constants of all complexes were measured by spectrophotometry, as the glass electrode could not accurately determine these values. The electrode calibration was carried out using a standard method described in detail elsewhere.^68^ Spectrophotometric titrations were performed with a Uvikon-XS (Bio-Tek Instruments) double-beam spectrophotometer, utilizing 1 cm path length quartz cuvettes and recording spectra in the range of 220 to 300 nm.

### Dissociation Kinetics

Kinetic reducing reactions of the [Cu(CBuDEDPA)] complex were studied in phosphate buffer (∼0.2 M) of varying pH in the presence of ascorbate, which is able to reduce Cu(II) to Cu(I), and neocuproine, an efficient Cu(I) scavenger due to 1:2 complex formation ([Cu(NC)_2_]^+^). The reactions were monitored by conventional spectroscopy following the increase in absorbance at 450 nm due to the formation of the Cu(I)-neocuproine complex (ε ≈ 7000 M^–1^·cm^–1^) and keeping the ratio [neocuproine]/[CuL] higher than ca. 2.5. Neocuproine was added upon dissolution in a small amount of dioxane (% v/v dioxane/water = 1.0–2% in the final solutions used for kinetics experiments). The ascorbate concentration (varying from 2 to 20 mM) was in high excess relative to complex concentration (∼10^–4^ M). All reactions were monitored by a Kontron-Uvikon 942 UV–vis spectrophotometer at 25 °C using 1 cm path length quartz cuvettes, with the complex being the last reagent added in the reaction mixture. In every case, absorbance (*A*) versus time (*t*) curves were appropriately fitted by a first-order integrated rate law [eq 2], with *A*_0_, *A*_t_, and *A*_∞_ being the absorbance values at times zero, *t*, and at the end of the reaction, respectively, and *k*_0_ being the calculated pseudo-first order rate constant.
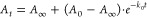
2

### Ligand Stock Concentration

To determine the concentration of H_2_CHXDEDPA, H_2_CpDEDPA and H_2_CBuDEDPA ligands used for radiolabeling experiments, spectrophotometric titrations were carried out with Cu^2+^. The formation of [Cu(CHXDEDPA)], [Cu(CpDEDPA)] and [Cu(CBuDEDPA)] was monitored at 300 nm using a 1 cm path length cuvette and a NanoDrop spectrophotometer. The pH was adjusted to 5.5 using 10 mM ammonium acetate buffer. For H_2_CHXDEDPA, a 1.78 mM ligand stock solution (60 μL) was titrated with addition of 10 μL (86.8 nmol) Cu^2+^ aliquots (as determined by ICP-OES) to determine the concentration of ligand by equivalents of Cu^2+^. A 1.31 mM H_2_CpDEDPA stock solution (80 μL) was titrated with addition of 10 μL (106.6 nmol) Cu^2+^ aliquots. For H_2_CBuDEDPA, a 2.07 mM ligand stock solution (50 μL) was titrated with addition of 10 μL (146.9 nmol) Cu^2+^ aliquots. For H_2_CBuDEDPA-NHBoc, a 1.41 mM (80 μL) ligand stock solution was titrated with addition of 10 μL (144.8 nmol) Cu^2+^ aliquots. The titration end point was determined when no further change in the absorbance intensity at 300 nm was added, testifying the complex formation (Figures S40–S43, Supporting Information).

### ^64^Cu Radiolabeling

Two μL of the ^64^CuCl_2_ stock solution was diluted in 0.05 M HCl to a total volume of 200 μL. The ligands were prepared from serial dilution of stock solutions (1, 0.25, 0.1, 0.025, 0.01, 0.0025, 0.001, 0.00025, 0.0001 mM) in deionized water. A 10 μL aliquot of each chelator stock solution (10, 2.5, 1.0, 0.25, 0.1, 0.025, 0.01, 0.0025, or 0.001 nmol, respectively) was diluted with 100 μL of ammonium acetate buffer (0.5 M, pH 5.5). A 50–90 μCi aliquot of ^64^CuCl_2_ (6 μL) was then added and the solution was mixed thoroughly. The complexation was carried out at room temperature and the radiochemical yield was measured after 15, 30, and 60 min via radio-TLC on aluminum-backed silica plates with methanol as mobile phase. The degree of binding was quantified via autoradiography and integration of the signal of the bound complex (*R*_*f*_ = 0.5 for CHXDEDPA, *R*_*f*_ = 0.5 for CpDEDPA, 0.25 for CBuDEDPA and 0.48 for CBuDEDPA-NHBoc) vs the free ^64^Cu (*R*_*f*_ = 0.04). The AMA was determined by ratio between the activity in the sample and the amount of ligand at 50% binding. All experiments were performed in triplicate.

Caution! Work with radioactive ^64^Cu should only be carried out by trained personnel at facilities equipped to safely handle and store these radionuclides.

### Stability in PBS

Experimental samples were prepared as previously described at a ligand concentration producing quantitative complex formation within 60 min (1 nmol). Once the labeling solution was prepared and quantitative formation of the desired ^64^Cu complex was verified, a 15 μL aliquot of the labeling solution was added to 100 μL of 1× Dulbecco’s phosphate-buffered saline. The stability of the complex was monitored by radio-TLC at 0, 1, 2, 3, 12, and 24 h.

### DTPA Challenge

Radiolabeling stock solutions were prepared as previously described using 1 nmol of ligand. The radiolabeling solution was mixed with 100 μL of 10 mM DTPA solution. Transchelation was monitored by radio-TLC at 0, 1, 3, 12, and 24 h.

### X-ray Diffraction Measurements

Single crystals of [Cu(CHXDEDPA)]·(CH_3_)_2_CO·H_2_O, [Cu(CpDEDPA)]·4H_2_O and [Cu(CBuDEDPA)] were analyzed by X-ray diffraction. Table S9 shows crystallographic data and the structure refinement parameters. Crystallographic data were collected at 100 K on a Bruker D8 Venture diffractometer with a Photon 100 CMOS detector and Mo Kα radiation (λ = 0.71073 Å) generated by an Incoatec high brillance microfocus source equipped with Incoatec Helios multilayer optics. The APEX3^69^ software was used for collecting frames of data, indexing reflections, and the determination of lattice parameters, SAINT^70^ for integration of intensity of reflections, and SADABS^71^ for scaling and empirical absorption correction. The structures were solved by dual-space methods using the program SHELXT.^72^ All non-hydrogen atoms were refined with anisotropic thermal parameters by full-matrix least-squares calculations on *F*^2^ using the program SHELXL-2014.^72^ For [Cu(CBuDEDPA)], the solvent mask command from Olex2 was used to correct the reflection data for the diffuse scattering due to the disordered molecules present in the unit cell. Hydrogen atoms of the compound were inserted at calculated positions and constrained with isotropic thermal parameters. Crystallographic data for the structures reported in this paper have been deposited with the Cambridge Crystallographic Data Centre as a supplementary publication no. 2382367–2382369, which can be obtained free of charge via www.ccdc.ac.uk/data_request/cif.

## Supporting Information Available

Details on the synthesis and characterization of the chelators, additional spectroscopic, electrochemical, kinetic and HPLC data (PDF)
