# Supplementary material for: Tuning the Properties of Rigidified Acyclic DEDPA2– Derivatives for Application in PET Using Copper-64
Source: Inorg Chem. 2024 Nov 7;63(46):22297–307. doi: 10.1021/acs.inorgchem.4c04050 (PMC11577316; doi:10.1021/acs.inorgchem.4c04050)
Supplement: Supplementary file 1 — ic4c04050_si_001.pdf [file ic4c04050_si_001.pdf]

Supporting Information for:

Tuning the Properties of Rigidified Acyclic DEDPA^{2-} Derivatives for Application in PET using copper-64

Daniel Torralba-Maldonado,[a] Axia Marlin,[b] Fátima Lucio-Martínez,[c] Antía Freire-García,[c] Jennifer Whetter,[b] Isabel Brandariz,[c] Emilia Iglesias,[c] Paulo Pérez-Lourido,[d] Rosa M. Ortuño,[a] Eszter Boros,[b]* Ona Illa,[a]* David Esteban-Gómez,[c] and Carlos Platas-Iglesias[c]*

[a] Departament de Química, Universitat Autònoma de Barcelona, 08193, Cerdanyola del Vallès, Spain

[b] Department of Chemistry, University of Wisconsin-Madison, Madison, Wisconsin 53706, United States

[c] Universidade da Coruña, Centro Interdisciplinar de Química e Bioloxía (CICA) and Departamento de Química, Facultade de Ciencias, 15071, A Coruña, Galicia, Spain

[d] Departamento de Química Inorgánica, Facultad de Química, Universidade de Vigo, As Lagoas, Marcosende, 36310 Pontevedra, Spain

Email: eboros@wisc.edu (E. B.); ona.illa@uab.cat (O. I.); carlos.platas.iglesias@udc.es (C. P.-I.)

Figure S1. Experimental high resolution mass spectrum (ESI$^+$) of **Me_2CpDEDPA**..10
Figure S2. ^{1}H NMR spectrum of **H_2CpDEDPA** (400 MHz, D_2O, pD 2.4, 298 K)..11
Figure S3. ^{13}C NMR spectrum of **H_2CpDEDPA** (400 MHz, D_2O, pD 2.4, 298 K)..11
Figure S4. ^{1}H NMR spectrum of compound **1** (300 MHz, $CDCl_3$, 298K). 12
Figure S5. ^{1}H NMR spectrum of compound **2** (300 MHz, $CDCl_3$, 298K). 13
Figure S6. ^{13}C NMR spectrum of compound **2** (75 MHz, $CDCl_3$, 298K). 13
Figure S7. ^{1}H NMR spectrum of compound **3** (400 MHz, $CDCl_3$, 298K). 14
Figure S8. ^{13}C NMR spectrum of compound **3** (100 MHz, $CDCl_3$, 298K). 14
Figure S9. ^{1}H NMR spectrum of compound **4** (400 MHz, DMSO-d_6, 298K)..15
Figure S10. ^{13}C NMR spectrum of compound **4** (100 MHz, DMSO-d_6, 298K)..15
Figure S11. ^{1}H NMR spectrum of compound **5** (400 MHz, $CDCl_3$, 298K). 16
Figure S12. ^{13}C NMR spectrum of compound 5 (100 MHz, $CDCl_3$, 298K). 16
Figure S13. ^{1}H NMR spectrum of compound **6** (300 MHz, MeOD, 298K)..17
Figure S14. ^{1}H NMR spectrum of compound **7** (300 MHz, MeOD, 298K)..18
Figure S15. ^{13}C NMR spectrum of compound **7** (100 MHz, MeOD, 298K)..18
Figure S16. ^{1}H NMR spectrum of compound **H_2CBuDEDPA-NHBoc** (300 MHz, D_2O, pD 6.0, 298K). 19

Figure S17. ^{13}C NMR spectrum of compound **H_2CBuDEDPA-NHBoc** (75 MHz, D_2O, pD 6.0, 298K). 19
Figure S18. Experimental high resolution mass spectrum (ESI^+) of **H_2CBuDEDPA-NHBoc**.......................20
Figure S19. HPLC chromatogram (272 nm) of **H_2CBuDEDPA-NHBoc** at t_R= 19.9 min. 21
Figure S20. Experimental high resolution mass spectrum (ESI^+) of **[Cu(CHXDEDPA)]**. 22
Figure S21. Experimental high resolution mass spectrum (ESI^+) of **[Cu(CpDEDPA)]**. 22
Figure S22. Experimental high resolution mass spectrum (ESI^+) of **[Cu(CBuDEDPA)]**. 23
Figure S23. Minimal distortion pathway between an octahedron and a trigonal prism (dashed line) and Shape measures obtained for octahedral [S(OC-6)] and trigonal prismatic [S(TPR-6)] in **[Cu(CBuDEDPA)]**, **[Cu(CpDEDPA)]** and **[Cu(CHXDEDPA)]**. 24
Figure S24. Cyclic voltammogram of **[Cu(CHXDEDPA)]** complex in aqueous solution in 0.15 M NaCl (1.4 mM, pH= 6.6), recorded at 10, 50, 100, 250, 400 and 500 mV·s^{-1} (top); and plots of the linear dependence of anodic and cathodic peak currents with the square root of the scan rate (bottom). 25
Figure S25. Cyclic voltammogram of **[Cu(CpDEDPA)]** complex in aqueous solution in 0.15 M NaCl (1.3 mM, pH= 5.9), recorded at 10, 50, 100, 250, 400 and 500 mV·s^{-1} (top); and plots of the linear dependence of anodic and cathodic peak currents with the square root of the scan rate (bottom). 26
Figure S26. Cyclic voltammogram of [Cu(CBuDEDPA)] complex in aqueous solution in 0.15 M NaCl (1.3 mM, pH= 5.0), recorded at 10, 50, 100, 250, 400 and 500 mV·s^{-1} (top); and plots of the linear dependence of anodic and cathodic peak currents with the square root of the scan rate (bottom). 27
Figure S27. Spectra of **[Cu(CHXDEDPA)]** (1.4 mM, blue curve), **[Cu(CpDEDPA)]** (1.3 mM, red curve), **[Cu(CBuDEDPA)]** (1.3 mM, green curve) and $CuCl_2$ (1.3 mM, black curve) dissolved in water at 298 K. ..28
Figure S34. Variation of the absorption at selected wavelength versus [AA] for **[Cu(CBuDEDPA)]** (91 μM); [NC]= 0.24 mM; [buffer]= 0.12 M; pH 6.3 recorded at 298 K. 31
Figure S35. Dependence of k_{obs} versus [AA] for **[Cu(CBuDEDPA)]** (91 μM); [NC]= 0.24 mM; [buffer]= 0.12 M and pH 6.3 recorded at 298 K...32
Figure S36. Variation of the absorption at selected wavelength versus [pH] for **[Cu(CBuDEDPA)]** (91 μM); [NC]= 0.24 mM; [buffer]= 0.12 M and [AA]= 5.4 mM recorded at 298 K. 33
Figure S38. HPLC traces of ligands. For **CHXDEDPA**, **CpDEDPA** and **CBuDEDPA** (Method A: (A) 0.1% TFA in water and (B) 0.1% TFA in CH_3CN). For **CBuDEDPA-NHBoc** (Method B: (A) 10 mM NH_4OAc pH 7 (B) CH_3CN). Gradient: 0-2 min: 5% B. 2-24 min: 5-95%B. 24-26 min: 95%B. 26-28 min: 95-5%B. 28-30 min: 5%B, UV detection at 220 and 254 nm. 34
Figure S39. HPLC traces of $^{nat}Cu^{2+}$ complexes. Complexation in MeOH/H_2O (pH 5, 15 min). For **CHXDEDPA**, **CpDEDPA** and **CBuDEDPA** (Method A: (A) 0.1% TFA in water and (B) 0.1% TFA in CH_3CN). For **CBuDEDPA-NHBoc** (Method B: (A) 10 mM NH_4OAc pH 7 (B) CH_3CN). Gradient: 0-2 min: 5% B. 2-24 min: 5-95%B. 24-26 min: 95%B. 26-28 min: 95-5%B. 28-30 min: 5%B, UV detection at 220 and 254 nm. 35
Figure S41. Spectrophotometric titration of **CpDEDPA** with Cu^{2+}. UV-Vis absorbance spectra of **CpDEDPA** upon Cu^{2+} addition (left) and UV-vis titration to endpoint to determine ligand concentration (right). 36
Figure S42. Spectrophotometric titration of **CBuDEDPA** with Cu^{2+}. UV-Vis absorbance spectra of **CBuDEDPA** upon Cu^{2+} addition (left) and UV-vis titration to endpoint to determine ligand concentration (right). 36
Figure S43. Spectrophotometric titration of **CBuDEDPA-NHBoc** with Cu^{2+}. UV-Vis absorbance spectra of **CBuDEDPA-NHBoc** upon Cu^{2+} addition (left) and UV-vis titration to endpoint to determine ligand concentration (right)...36

Table S1. MPLC method applied for the purification of **H_2CBuDEDPA-NHBoc** (with mobile phases A= ammonium acetate 10 mM aqueous solution and B= CH_3CN + 10% A). 21
Table S2. HPLC method applied for the purification of **H_2CBuDEDPA-NHBoc** (with mobile phases A= ammonium acetate 10 mM aqueous solution and B= CH_3CN + 10% A). 21
Table S3. Selected bond angles(°) of the Cu(II) coordination environments in **[Cu(CHXDEDPA)]**, **[Cu(CpDEDPA)]** and **[Cu(CBuDEDPA)]** determined by single-crystal X-ray measurements. 24

Table S4. Parameters obtained when studying the influence of [AA] concentration for **[Cu(CBuDEDPA)]** (91 μM); ([NC]=0.24 mM; buffer $HPO_4^{-2}/H_2PO_4^-$ 0.122 M, pH 6.3; I= 0.15 M)..32
Table S5. Parameters obtained when studying the influence of pH for **[Cu(CBuDEDPA)]** (91 μM); ([NC]=0.24 mM; [5.4 mM; buffer $HPO_4^{-2}/H_2PO_4^-$ 0.122 M; I= 0.15 M) recorded at 298 K. 33
Table S6. Apparent Molar Activity (AMA) in mCi.μmol^{-1}. 37
Table S7. Percentage of the intact ^{64}Cu-complexes in PBS buffer. 37
Table S8. Percentage of the intact ^{64}Cu-complexes in DTPA solution. 37
Table S9. Crystal data and structure refinement for **[Cu(CHXDEDPA)]·$(CH_3)_2CO$·H_2O**, **[Cu(CpDEDPA)]·4H_2O** and **[Cu(CBuDEDPA)]**………………………………………………………..…39

Synthesis of the H_2CpDEDPA ligand and orthogonally protected H_2CBuDEDPA-NHBoc.

General considerations

Solvents and reagents were purchased from commercial sources and were directly used without further purification. Medium performance liquid chromatography (MPLC) was performed in a Puriflash XS 420 InterChim Chromatographer equipped with a UV-DAD detector in reverse phase, using a 20 g BGB Aquarius C18AQ reversed-phase column (100 Å, spherical, 15 μm). The experimental conditions are described below for each case. Preparative high performance liquid chromatography (HPLC) was performed using an Agilent 1260 Infinity II instrument equipped with an UV Variable Wavelenghth Detector (VWD), in manual injection and collection mode, using an Agilent InfinityLab ZORBAX 5 Eclipse Plus C18 (5 μm, 21.2 x 250 mm) and 10 mM ammonium acetate aqueous solution (phase A) and CH_3CN with 10% of phase A (phase B) as the mobile phases, operating at a flow rate of 20 mL/min. High-resolution electrospray-ionization time-of-flight (ESI-TOF) mass spectra were recorded in positive mode using a LTQ-Orbitrap Discovery Mass Spectrometer coupled to a Thermo Accela HPLC. Aqueous solutions were lyophilized using a Biobase BK-FD10 Series apparatus. ^{1}H and ^{13}C NMR spectra of the ligands and their precursors were recorded on a Bruker AVANCE III 300, a Bruker AVANCE 400 or a Bruker AVANCE 500 spectrometers.

Experimental

6,6'-((((*rac*-cyclopentane-1,2-diyl)bis(azanediyl))bis(methylene))dipicolinic acid (H_2CpDEDPA): A solution of methyl 6-formylpyridine-2-carboxylate (223.3 mg, 1.35 mmol) in MeOH (30 mL) was added dropwise to a refluxing solution of *rac*-1,2-cyclopentanediamine dihydrochloride (115.9 mg, 0.67 mmol) and N,N-diisopropylethylamine (DIPEA, 0.23 mL, 1.34 mmol) in MeOH (10 mL). The resulting mixture was refluxed for 4 h. After this time, it was cooled to 0 ^{0}C and $NaBH_4$ (36.6 mg, 0.97 mmol) was added. The mixture was stirred at 0 ^{0}C for additional 1.5 h, until complete reduction of the imine was confirmed by MS (ESI$^+$, MeOH/H_2O): m/z 399.147;

calculated for $[C_{21}H_{26}N_4O_4]H^+$ 399.203 (Figure S1). Then saturated $NaHCO_3$ aqueous solution (50 mL) was added, and it was stirred for 10 min. The resulting solution was extracted with CH_2Cl_2 (3 × 50 mL). The combined organic extracts were dried over Na_2SO_4 and evaporated to give an orange oil that was hydrolyzed with 20 mL of HCl 6 M (reflux, overnight). The product was lyophilized to afford a white solid that was purified by MPLC on reverse phase using a C18AQ (20 g) column and H_2O (0.1 % TFA) / CH_3CN (0.1 % TFA) as mobile phase (compound eluted at 42 % CH_3CN). (200.6 mg, 48% yield) ^{1}H NMR (300 MHz, D_2O) δ 8.16 (d, J = 7.8, 1.3 Hz, 2H), 8.10 (dd, J = J' = 7.8 Hz, 2H), 7.75 (d, J = 7.8, 1.3 Hz, 2H), 4.75 (d, J = 15 Hz, 2H), 4.65 (d, J = 15 Hz, 2H), 4.19 (m, 2H), 2.39 (m, 2H), 1.94 (m, 4H). ^{13}C NMR (75 MHz, D_2O) δ 167.81, 150.43, 147.24, 139.91, 127.32, 125.47, 60.49, 48.90, 28.29, 21.86. Elemental analysis calcd (%) for $[C_{19}H_{22}N_4O_4]\cdot 2TFA\cdot 0.5H_2O$: C 45.26, H 3.80, N 8.44; found: C 45.25, H 3.89, N 8.79. IR (ATR, cm−1): 1716 and 1660 ν(C=O).

6,6'-((((1*R*,3*S*)-2-((*tert*-butoxycarbonyl)amino)cyclobutane-1,3-diyl)bis(azanediyl))bis(methylene))dipicolinic acid (H_2CBuDEDPA-NHBoc): The ligand was synthesized according to Scheme S1, starting from previously reported trifunctionalized cyclobutane derivative **1**[1] which affords a protected alcohol and a carboxyl group as precursors of the *cis*-1,3-diamino system.

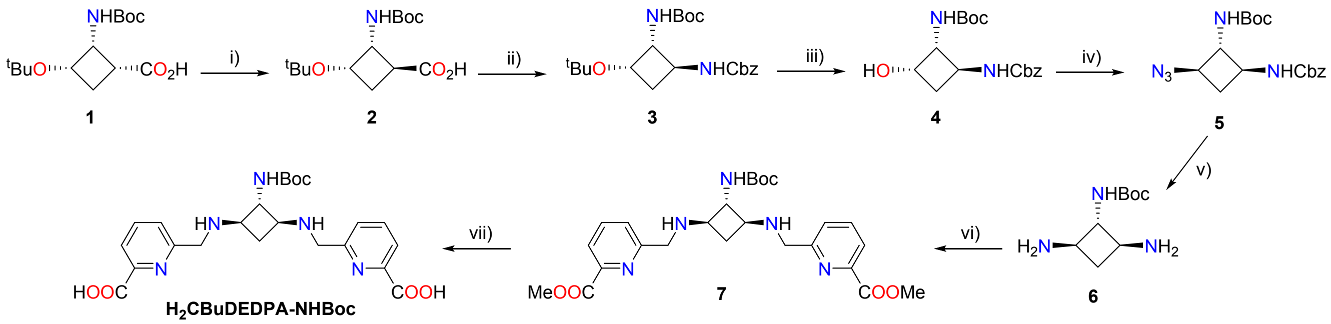

Scheme S1. Synthesis of the bifunctional precursor **H_2CBuDEDPA-NHBoc**. *Reagents and conditions*: i) a) Boc_2O, pyridine, NH_4HCO_3, dioxane, 0 °C to rt, 4 h; b) 6.25 M NaOH, MeOH, reflux, 18 h, 76%; ii) a) DPPA, Et_3N, toluene, reflux, 2 h; b) BnOH, 80 °C, 6 h, 81%; iii) a) TFA, 0 °C to rt, 3 h; b) Boc_2O, Et_3N, THF, 0 °C to rt, 4 h, 87%; iv) DPPA, PPh_3, DIAD, THF, 0 °C to rt, 3 h, 79%; v) H_2, Pd/C, MeOH, rt, 16 h, quantitative; vi) a) methyl 6-formylpicolinate, MeOH, 40 °C, 2.5 h; b) $NaBH_3CN$ 0 °C to rt, 4 h, 50%.; vii) LiOH, H_2O:THF, rt, 3 h, 53%.

To epimerize the α-position to the carboxylic group, compound **1** was converted temporarily into a mixed acid anhydride, so compound **2** was thus obtained as the major product in a 5:1 mixture together with starting material **1** in 76% yield over the two steps. This mixture was subjected to a Curtius rearrangement using diphenylphosphoryl azide under reflux and the resulting isocyanate was reacted with benzyl alcohol to yield dicarbamate **3** in 81% after purification. Deprotection of the hydroxyl group in **3** was carried out using neat TFA. This reaction also implied the acidolysis of the *tert*-butylcarbamate. The resulting amine was re-protected using di-*tert*-butyl dicarbonate, **4** being

obtained in 87% yield over the two steps. After that, a Mitsunobu reaction was carried out in order to install an azide in place of the hydroxyl group with inversion of configuration. In this way, **5** was obtained in 79% yield. Subsequently, Pd-catalyzed hydrogenolysis of the benzyl carbamate and reduction of the azide group led to triamine **6** in quantitative yield. **6** was submitted to double reductive amination with methyl 6-formylpicolinate, rendering orthogonally protected ligand **7** in 50% yield. The *all-trans*-triamine ligand **7** was obtained in a 6-step sequence in 21% overall yield. Finally, saponification of the methyl ester groups rendered **H_2CBuDEDPA-NHBoc** in 53% yield.

(1*RS*,2*SR*,3*RS*)-3-(*tert*-Butoxy)-2-((*tert*-butoxycarbonylamino)cyclobutane-1-carboxylic acid (2). Carboxylic acid **1**[1] (0.70 g, 2.44 mmol) was dissolved in 1,4-dioxane (8 mL) and the solution was cooled to 0 ºC before adding pyridine (0.49 mL, 6.09 mmol, 2.50 eq.). The solution was heated to room temperature and Boc_2O was added to the mixture, which was stirred for 30 min. Then, ammonium carbonate (0.58 g, 7.31 mmol, 3.00 eq.) was added and the crude mixture was stirred at room temperature for 4 h. Water (20 mL) was added, and the solvent was removed under vacuum to dryness. The crude obtained was dissolved in EtOAc (20 mL) and the organic phase was washed with water (4 x 20 mL). The organic phase was dried over $MgSO_4$ and the solvent was evaporated under reduced pressure. The obtained solid was dissolved in MeOH (30 mL), treated with 6.25 M NaOH aqueous solution (16 mL), and the mixture was heated to reflux overnight. Methanol was then removed by careful evaporation under vacuum, and the aqueous phase was cooled to 0 °C and treated with concentrated HCl to pH 3. The aqueous phase was extracted with CH_2Cl_2 (6 × 30 mL), the combined organic layers were dried over $MgSO_4$, and the solvent was evaporated under reduced pressure to obtain a mixture of *trans-cis* : *all-cis* products, **2** and **1**, in a 5:1 ratio as a white powder (0.53 g, 1.84 mmol, 76 % yield). Purification of diastereoisomers using column chromatography was not fully successful. As a result, both products were used in the next step and the by-products were separated by column chromatography. One pure sample of **2** was used for characterization. R_f (hexane:EtOAc, 1:1) = 0.37; Mp: 107 - 110 ºC (from hexane:EtOAc); IR (ATR, cm^1) ν 3393, 3280, 2972, 1711, 1665, 1387, 1129; ^{1}H NMR (300 MHz, $CDCl_3$) δ 5.73 (br s, 1H), 4.31 (td, $J = J'$ = 7.6 Hz, J'' = 3.0 Hz, 1H), 4.18 (m, 1H), 3.20 (dt, J = 10.7 Hz, $J' = J''$ = 7.6 Hz, 1H), 2.61 (dt, J = 14 H, J' = J'' = 7.9 Hz, 1H), 2.18 (m, 1H), 1.48 (s, 9H), 1.20 (s, 9H); ^{13}C NMR (75 MHz, $CDCl_3$) δ 174.3, 157.7, 81.9, 75.5, 63.5, 52.1, 46.6, 31.5, 28.4 (6C); HRMS (ESI$^+$) *m/z* $[M+Na]^+$ Calcd for $C_{14}H_{25}NNaO_5$, 310.1625; found, 310.1630.

Benzyl *tert*-butyl ((1*RS*,2*SR*,3*RS*)-3-(*tert*-butoxy)cyclobutane-1,2-diyl)dicarbamate (3). Carboxylic acid **2** (1.20 g, 4.18 mmol), under a N_2 atmosphere, was dissolved in anhydrous toluene (22 mL). Anhydrous triethylamine (0.64 mL, 4.60 mmol, 1.10 eq.) was added to the solution and the

mixture was stirred at 0 ºC. Diphenylphosphoryl azide (DPPA) (0.99 mL, 4.60 mmol, 1.10 eq.) was added, and the mixture was refluxed for 2 h under a N_2 atmosphere. The crude was cooled to 60 ºC, and benzyl alcohol (0.87 mL, 8.35 mmol, 2.00 eq.) was added in one portion. The mixture was then heated at 80 °C for 6 h. After this time, toluene was evaporated under reduced pressure. The crude obtained was dissolved in H_2O (30 mL) and extracted with CH_2Cl_2 (6 x 30 mL). The organic layers were combined, dried over $MgSO_4$ and the solvent was evaporated under reduced pressure. The remaining benzyl alcohol was microdistilled, and the residual oil was purified by silica column chromatography (hexane:EtOAc, 1:1) to afford compound **3** (1.33 g, 3.38 mmol, 81% yield) as a white solid. R_f (hexane:EtOAc, 1:1) = 0.55; Mp: 111 -114 ºC (from hexane:EtOAc); IR (ATR, cm^1) ν 3377, 3262, 2943, 1708, 1653, 1384, 1122; ^1H NMR (400 MHz, $CDCl_3$) δ 7.36 - 7.28 (m, 5H), 5.26 – 5.22 (br s, 2H), 5.07 (m, 2H), 4.22 (t, *J* = 6.2 Hz, 1H), 4.13 (m, 1H), 4.00 (ddd, *J* = *J'* = *J''* = 6.9 Hz, 1H), 2.17 (m, 1H), 1.84 (m, 1H), 1.43 (s, 9H), 1.15 (s, 9H); ^{13}C NMR (75 MHz, $CDCl_3$) δ 155.7, 155.6, 136.7, 128.6 (3C), 128.2 (2C), 79.6, 74.7, 66.7, 65.5, 55.0, 53.2, 35.2, 28.4 (6C); HRMS (ESI^+) *m/z* $[M+Na]^+$ Calcd for $C_{21}H_{32}N_2NaO_5$, 415.2203; found, 415.2198.

Benzyl *tert*-butyl ((1S*R*,2*SR*,3*RS*)-3-hydroxycyclobutane-1,2-diyl)-dicarbamate (4). Compound **3** (0.80 g, 2.04 mmol) was dissolved in neat TFA (4.00 mL, 52.30 mmol, 26.00 eq.) under an inert atmosphere at 0 ºC. The mixture was stirred for 3 h while the temperature was slowly increased to room temperature. After the complete evaporation of TFA, the residue was diluted with anhydrous THF (5 mL). The mixture was cooled to 0 ºC before adding anhydrous Et_3N dropwise (0.60 mL, 4.28 mmol, 2.10 eq.) and Boc_2O (0.67 g, 3.06 mmol, 1.50 eq.) successively. The mixture was then stirred overnight at room temperature. The solvent was removed under reduced pressure and the crude obtained was dissolved in H_2O (30 mL) and extracted with EtOAc (4 x 30 mL). The combined organic phases were successively washed with 2 M NaOH (5 mL) and brine and dried over $MgSO_4$. The solvents were evaporated under reduced pressure, furnishing compound **4** (0.59 g, 1.77 mmol, 87% yield) as a white powder. R_f (hexane:EtOAc, 1:4) = 0.39; Mp: 123 -127 ºC (from hexane:EtOAc); IR (ATR, cm^{-1}) ν 3359, 2973, 1722, 1675, 1532, 1256, 1162, 1029; ^1H NMR (400 MHz, DMSO-d_6) δ 7.60 (d, *J* = 8.5 Hz, 1H), 7.36 - 7.29 (m, 5H), 6.63 (d, *J* = 8.5 Hz, 1H), 5.02 - 4.94 (m, 3H), 4.16 (ap quint, *J* =8.7 Hz 1H), 4.06 (m, 1H), 3.92 (ddd, *J* = *J'* = *J''* = 8.5 Hz, 1H), 1.85-1.73 (m, 2H), 1.37 (s, 9H); ^{13}C NMR (100 MHz, DMSO-d_6) δ 155.1, 154.6, 137.1, 128.3 (3C), 127.8 (2C), 77.8, 65.2, 64.5, 54.7, 51.2, 33.7, 28.2 (3C); HRMS (ESI^+) *m/z* $[M+Na]^+$ Calcd for $C_{17}H_{24}N_2NaO_5$, 359.1577; found, 359.1562.

Benzyl *tert*-butyl ((1*RS*,2*SR*,3*SR*)-3-azidocyclobutane-1,2-diyl)dicarbamate (5). To an ice-cooled solution of **4** (0.58 g, 1.73 mmol) in anhydrous THF (30 mL) were added PPh_3 (1.36 g, 5.17 mmol,

3.00 eq.), DIAD (1.02 mL, 5.17 mmol, 3.00 eq.), and DPPA (1.13 mL, 5.17 mmol, 3.00 eq.) successively under N_2 atmosphere. After 10 min, the temperature was raised to 50 ºC and the reaction was stirred for 3 h. The solvent was evaporated under vacuum, and the residue was purified by flash chromatography on silica gel (hexane:EtOAc, 3:1) to provide product **5** (0.49 g, 1.36 mmol, 79% yield) as a white solid. R_f (hexane:EtOAc, 2:1) = 0.37; Mp: 139 -142 ºC (from hexane:EtOAc); IR (ATR, cm^{-1}) ν 3341, 2983, 2091, 1681, 1523, 1240, 1038; ^{1}H NMR (400 MHz, $CDCl_3$) δ 7.33 (m, 5H), 5.57 (br s, 1H), 5.32 (br s, 1H), 5.07 (m, 2H), 3.89 - 3.79 (m, 2H), 3.42 (m, 1H), 2.54 (ddd, *J* = *J'* = *J''* = 7.9 Hz, 1H), 1.60 (ddd, *J* = *J'* = *J''* = 7.9 Hz, 1H), 1.43 (s, 9H); ^{13}C NMR (100 MHz, $CDCl_3$) δ 156.0, 155.1, 136.3, 128.7 (3C), 128.3 (2C), 80.3, 67.0, 59.6, 55.9, 46.8, 30.6, 28.5 (3C); HRMS (ESI^+) *m/z* $[M+Na]^+$ Calcd for $C_{17}H_{23}N_5NaO_4$, 384.1642; found, 384.1642.

***tert*-Butyl (*trans*-(2*RS*,4*SR*)-2,4-diaminocyclobutyl)carbamate (6).** To a solution of compound **5** (0.22 g, 0.61 mmol) in MeOH (5 mL) was added Pd/C (10%) (0.07 g, 0.06 mmol, 0.10 eq.). The mixture was stirred overnight (16 h) under a hydrogen atmosphere at room temperature. After filtration on a pad of Celite®, the filtrate was concentrated by evaporation to afford product **6** (0.12 g, 0.60 mmol, quantitative yield) as a pale-yellow sticky solid. This compound was used in the following step without further purification. ^{1}H NMR (300 MHz, MeOD) δ 3.25 (m, 1H), 2.77 (m, 2H), 2.37 (m, 1H), 1.46 (s, 9H), 1.24 (ddd, *J* = 10.2 Hz, *J'* = *J''* = 9.2 Hz, 1H).

Dimethyl 6,6'-(((*trans*-(1*RS*,3*SR*)-2-(*tert*-butoxycarbonylamino)cyclobutane-1,3-diyl)-bis(azanediyl))bis(methylene))dipicolinate (7). To a solution of **6** (0.12 g, 0.60 mmol) in anhydrous CH_2Cl_2 (6 mL) was added methyl 6-formylpicolinate[2] (0.21 g, 1.25 mmol, 2.10 eq) under a nitrogen atmosphere and the reaction mixture was heated to 40 ºC for 2.5 h. Then, volatiles were evaporated, and the residue was dissolved in MeOH. The mixture was cooled to 0 ºC and $NaBH_3CN$ was added (0.11 g, 1.79 mmol, 3.00 eq.) in small portions along 30 min. The reaction mixture was allowed to attain room temperature stirred for 3 h. Reaction was quenched by adding H_2O (15 mL) and MeOH was evaporated under vacuum. The aqueous phase was extracted with CH_2Cl_2 (4 x 15 mL), the organic layers were combined, dried over $MgSO_4$, and concentrated under reduced pressure. The residue was purified by column chromatography (Al_2O_3, DCM:MeOH, 98:2) to afford **7** (0.11 g, 0.30 mmol, 50% yield) as a white solid. R_f (DCM:MeOH, 98:2) = 0.23; Mp: 157 - 159 ºC (from DCM); IR (ATR, cm^{-1}) ν 3329, 2994, 1744, 1681, 1511, 1230, 1039; ^{1}H NMR (300 MHz, MeOD) δ 8.16 (d, *J* = 8.1 Hz, 2H), 8.05 (dd, *J* = *J'* = 8.1 Hz, 2H), 7.76 (d, *J* = 8.1 Hz, 2H), 4.14 – 4.01 (m, 10H), 3.45 (dd, *J* = *J'* = 7.1 Hz, 1H), 3.00 (ddd, *J* = *J'* = *J''* = 8.0 Hz, 2H), 2.34 (m, 1H), 1.56 (s, 9H), 1.38 (m, 1H); ^{13}C NMR (75 MHz, MeOD) δ 166.9 (2C), 161.4 (2C), 157.1, 148.3 (2C), 139.3 (2C), 127.4 (2C), 124.7 (2C),

79.9, 61.3, 55.7 (2C), 53.2 (2C), 52.8 (2C), 31.9, 28.8 (3C); HRMS (ESI$^+$) *m/z* $[M+H]^+$ Calcd for $C_{25}H_{34}N_5O_6$, 500.2504; found, 500.2485.

6,6'-((((1R,3S)-2-((tert-butoxycarbonyl)amino)cyclobutane-1,3-diyl)bis(azanediyl))bis(methylene))dipicolinic acid (H_2CBuDEDPA-NHBoc): To a solution of **7** (0.0831 g, 0.1663 mmol) in MeOH (3 mL), a solution of LiOH (0.0159 g, 0.6654 mmol, 4 eq) in H_2O:THF (1:1,2 mL) was added. The mixture was stirred at room temperature for 3 h and then the solvent was eliminated in the rotary evaporator. The resulting brown oil was dissolved in water (1.5 mL) and was purified by reverse phase MPLC with a 10 mM ammonium acetate aqueous solution (phase A) and CH_3CN with 10% of phase A (phase B) as the mobile phases. (method in Table S1). The desired product eluted at 12% phase B (retention time 20.3 min, 15.0 CV). Then the fraction of interest was lyophilized, obtaining an off-white solid (0.0420 g, 53% yield). ^1H NMR (300 MHz, D_2O, pD 6.0, 298 K) δ 7.87 – 7.71 (m, 4H), 7.41 – 7.29 (m, 2H), 4.39 – 4.21 (m, 4H), 3.65 (q, J = 8.5, 8.1 Hz, 2H), 2.64 – 2.49 (m, 1H), 2.17 – 2.01 (m, 1H), 1.83 (s, 5H), 1.22 (s, 4H), 1.08 (s, 1H). ^{13}C NMR (75 MHz, D_2O, pD 6.0, 298K) δ 180.1, 171.9, 155.6, 152.3, 149.8, 139.1, 124.8, 123.8, 81.6, 52.8, 51.9, 49.3, 27.4, 22.5. Elemental analysis calcd (%) for $(C_{23}H_{28}N_5O_6)NH_4\cdot 2KCl$: C 43.32, H 5.06, N 13.18; found C 43.31, H 5.19, N 13.31. Experimental MS (ESI$^+$, MeOH/H_2O): m/z 472.2193, 494.2012; calculated for $[C_{23}H_{30}N_5O_6]^+$ 472.2191, calculated for $[C_{23}H_{29}N_5O_6Na]^+$ 494.2010.
The chelator was purified by preparative HPLC before radiolabeling experiments to ensure high purity (Table S2, Figure S19).

General synthesis of Cu(II) complexes derived from the ligands H_2CHXDEDPA, H_2CpDEDPA and H_2CBuDEDPA: To a solution of the ligand in 3 mL of water $Cu(OTf)_2$ was added (1.1 eq.). Additional 2 mL of water were added, and the pH was adjusted to *ca* 7 with KOH 1M. The mixture was refluxed for 30 minutes, and a brown precipitate appears. After, the pH is increased over 8 and the solution was filtered using a 0.2 μm microfilter. The solution was then lyophilized to afford a blue solid that was purified by MPLC on reverser phase using a C18AQ (20 g) column and H_2O/CH_3CN as mobile phase.
[Cu(CHXDEDPA)]: Elutes at 51 % CH_3CN. Blue solid (30.8 mg, 61% yield). MS (ESI$^+$, MeOH/H_2O): m/z 446.1010; calcd for $[CuC_{20}H_{22}N_4O_4]H^+$ 446.1007. Elemental analysis calcd (%) for $[CuC_{20}H_{22}N_4O_4]\cdot 3.5(H_2O)$: C 47.19, H 5.74, N 11.01; found: C 47.22, H 5.54, N 10.60. IR (ATR, cm^{-1}): 1625 and 1591 ν(C=O).
[Cu(CpDEDPA)]: Elutes at 46 % CH_3CN. Blue solid (19.8 mg, 41% yield). MS (ESI$^+$, MeOH/H_2O): m/z 432.0853; calcd for $[CuC_{19}H_{20}N_4O_4]H^+$ 432.0853. Elemental analysis calcd (%) for

$[CuC_{19}H_{20}N_4O_4]\cdot 3.4(H_2O)$: C 46.27, H 5.48, N 11.36; found: C 46.67, H 4.95, N 10.60. IR (ATR, cm^{-1}): 1626 and 1592 ν(C=O).

[Cu(CBuDEDPA)]: Elutes at 45 % CH_3CN. Blue solid (26.0 mg, 0.06 mmol, 64% yield). MS (ESI^+, MeOH/H_2O): m/z 456.0258; calcd for $[CuC_{18}H_{18}N_4O_4]K^+$ 456.0256. Elemental analysis calcd (%) for $[CuC_{18}H_{20}N_4O_4]\cdot 2.3(H_2O)$: C 47.09, H 4.96, N 12.20; found: C 47.18, H 4.91, N 11.76. IR (ATR, cm^{-1}): 1635 and 1590 ν(C=O).

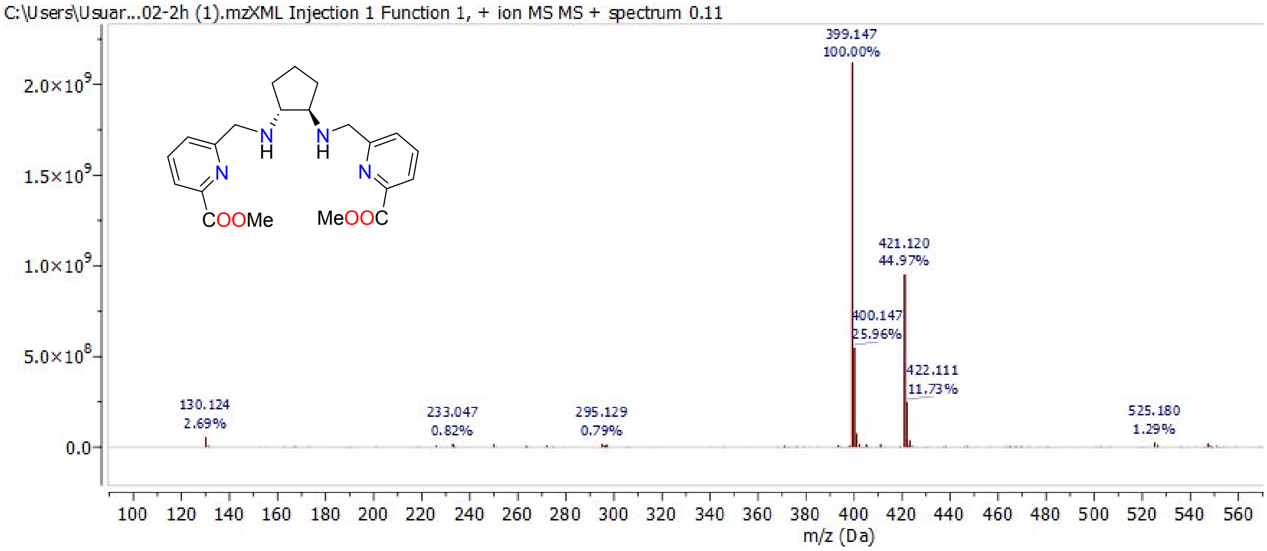

Figure S1. Experimental high resolution mass spectrum (ESI$^+$) of **Me$_2$CpDEDPA**.

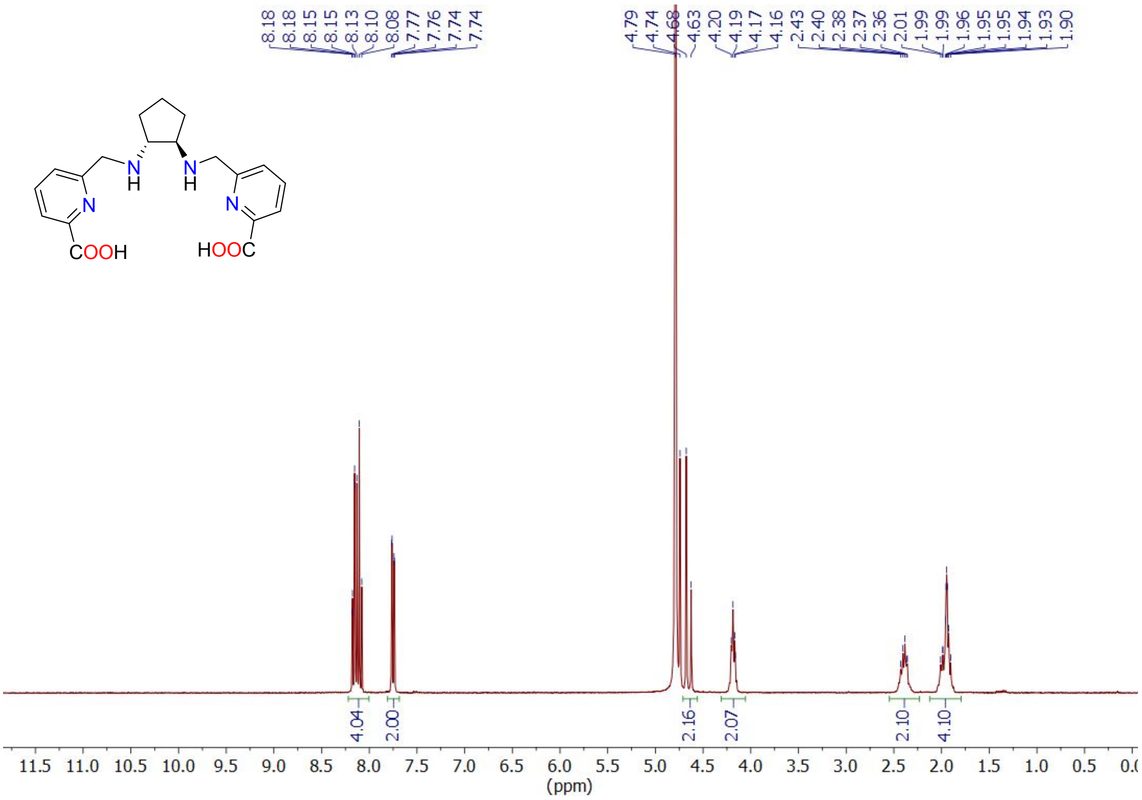

Figure S2. ^{1}H NMR spectrum of **H_2CpDEDPA** (400 MHz, D_2O, pD 2.4, 298 K).

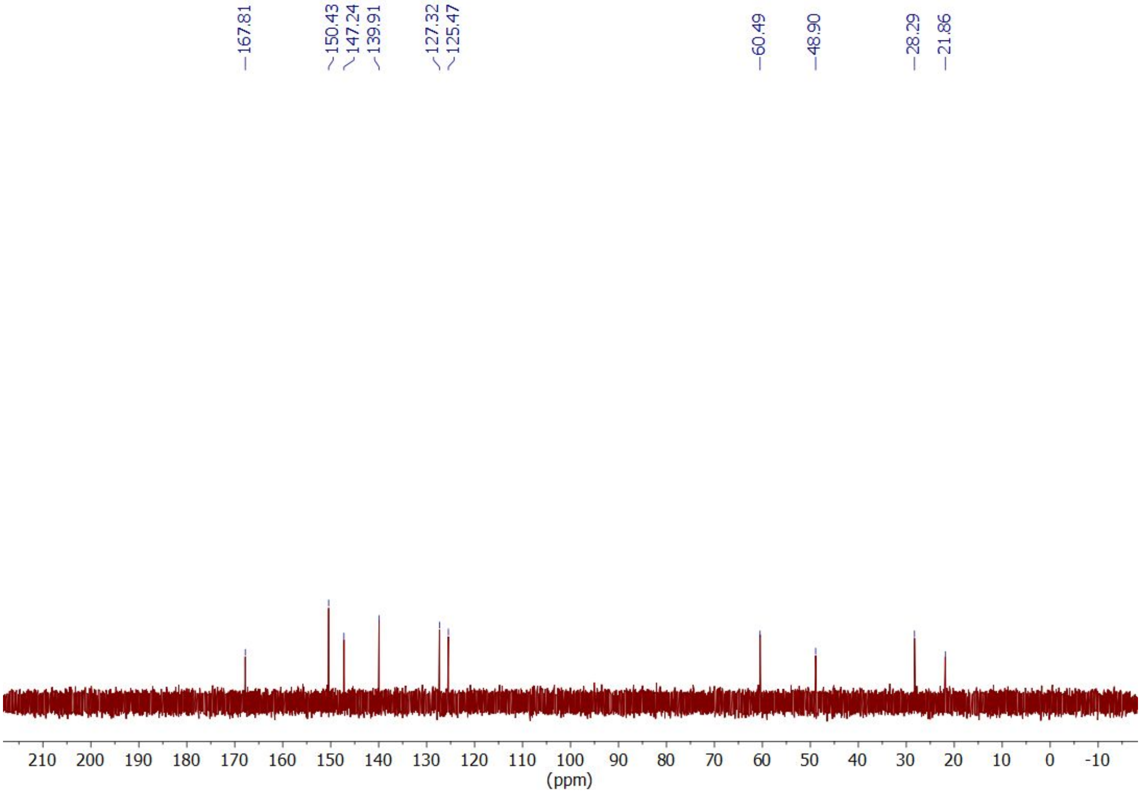

Figure S3. ^{13}C NMR spectrum of **H_2CpDEDPA** (400 MHz, D_2O, pD 2.4, 298 K).

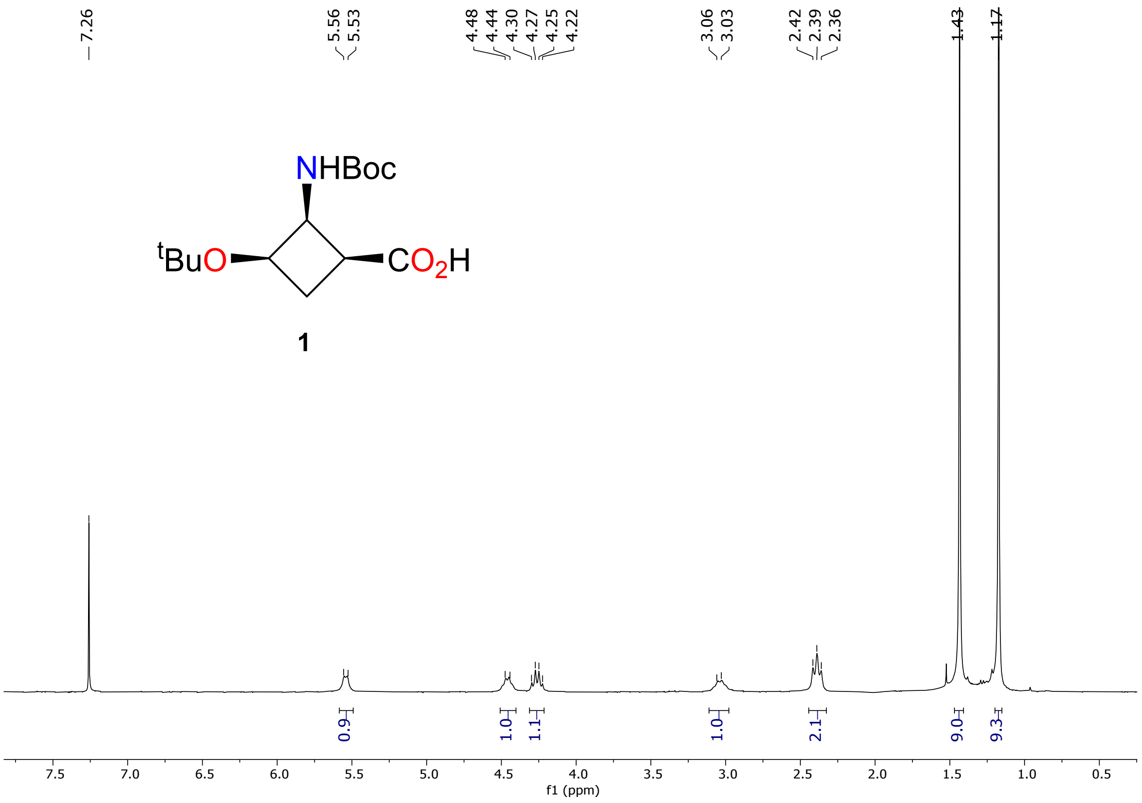

Figure S4. ^{1}H NMR spectrum of compound **1** (300 MHz, $CDCl_3$, 298K).

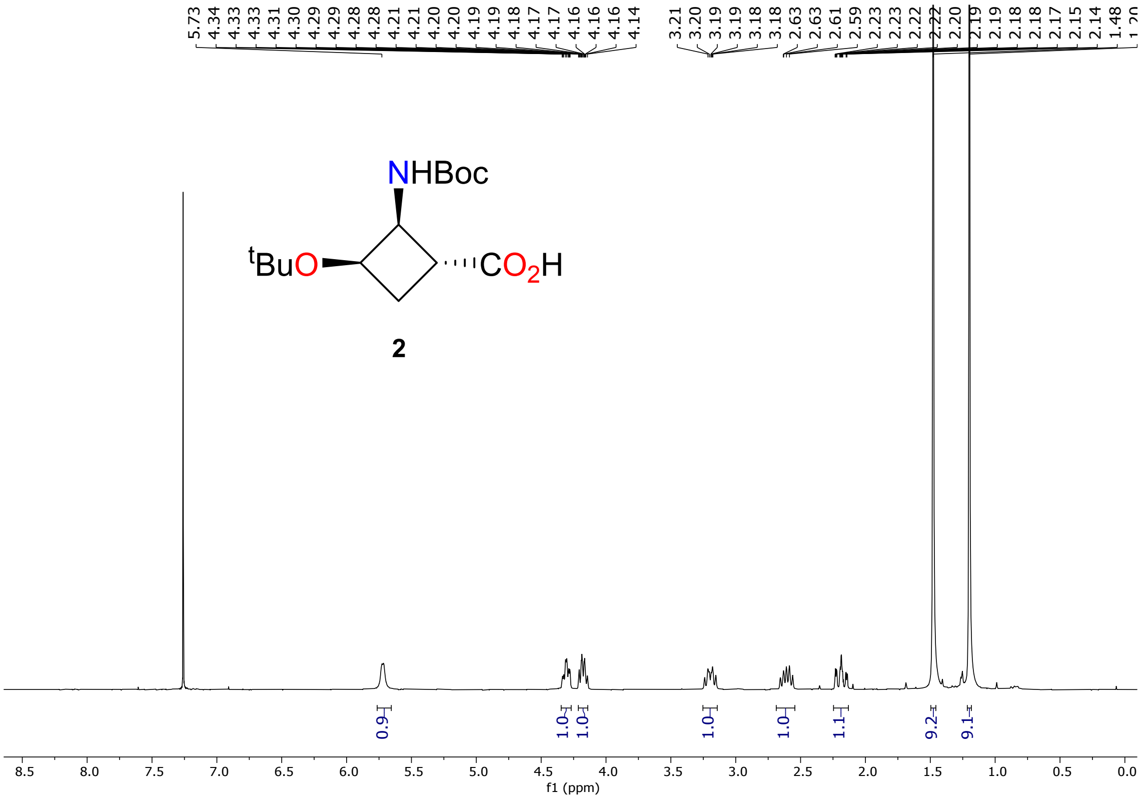

Figure S5. ^{1}H NMR spectrum of compound **2** (300 MHz, $CDCl_3$, 298K).

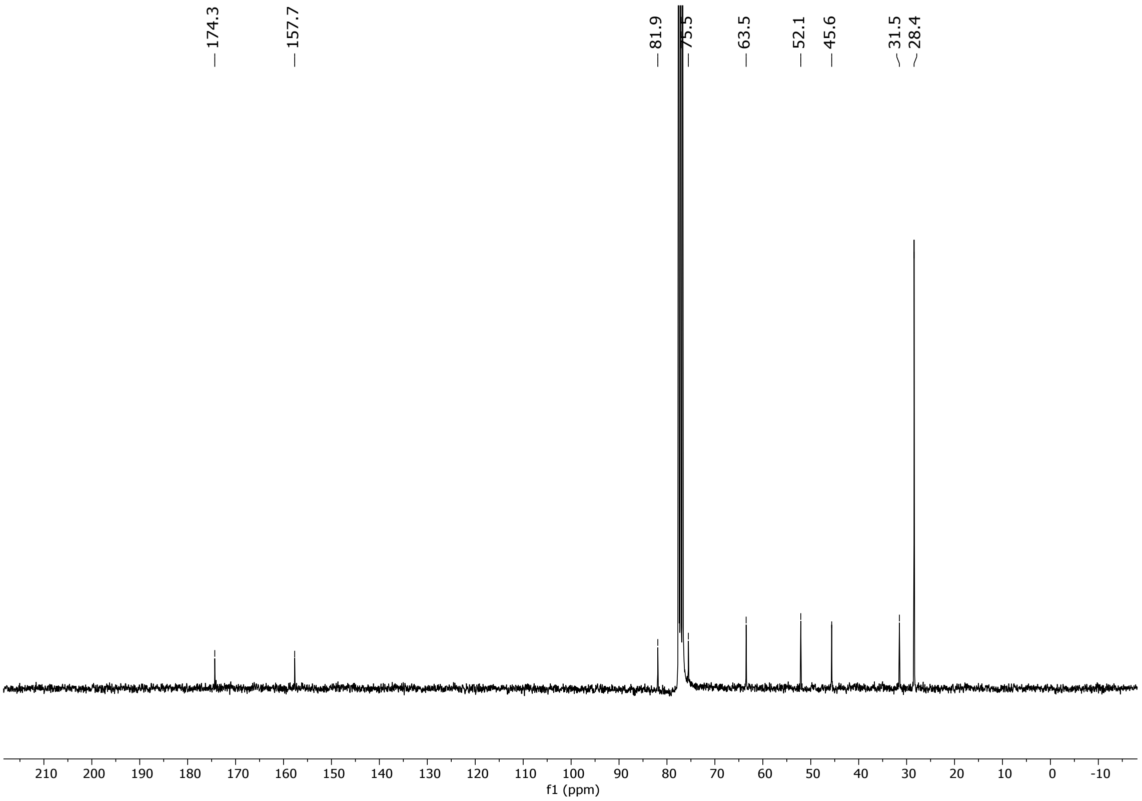

Figure S6. ^{13}C NMR spectrum of compound **2** (75 MHz, $CDCl_3$, 298K).

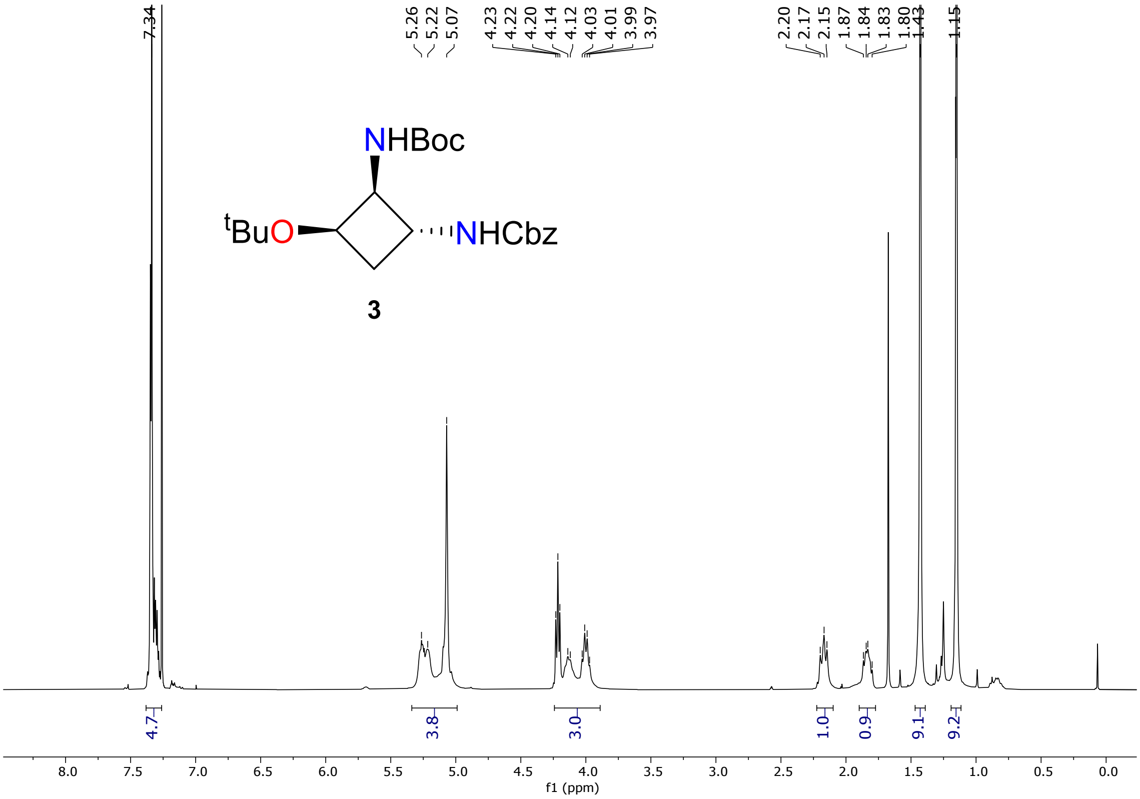

Figure S7. ^{1}H NMR spectrum of compound **3** (400 MHz, $CDCl_3$, 298K).

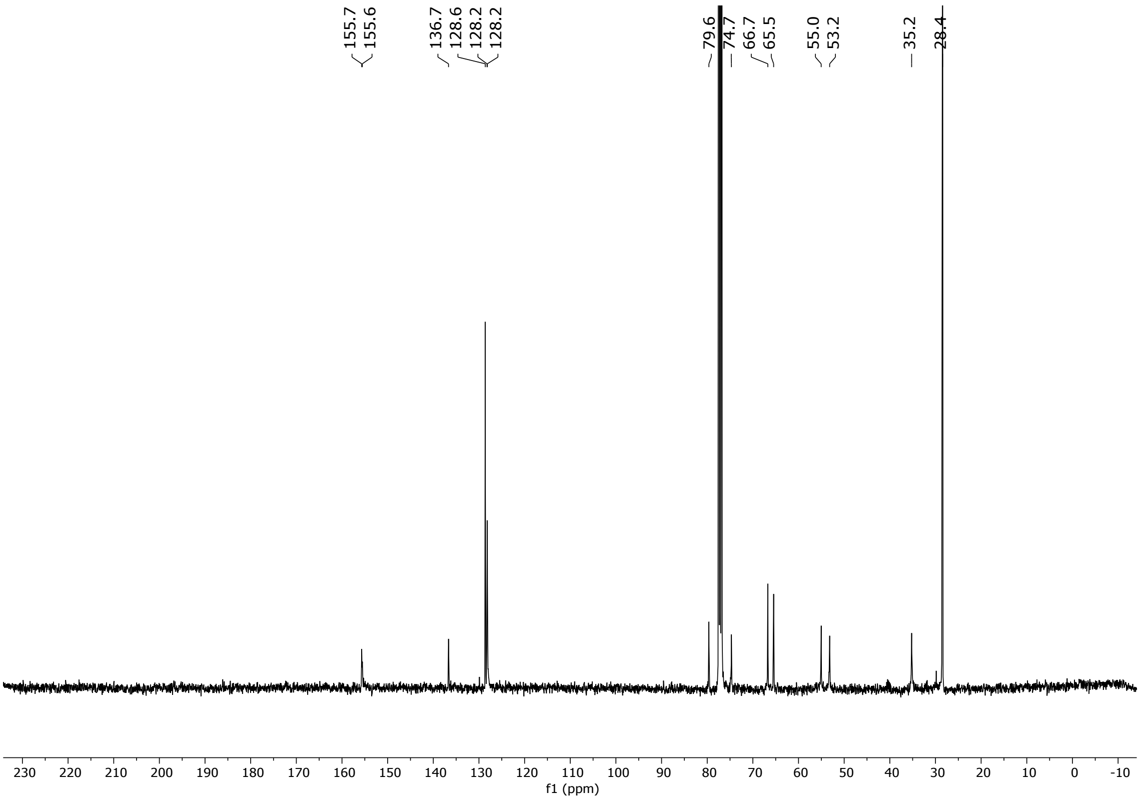

Figure S8. ^{13}C NMR spectrum of compound **3** (100 MHz, $CDCl_3$, 298K).

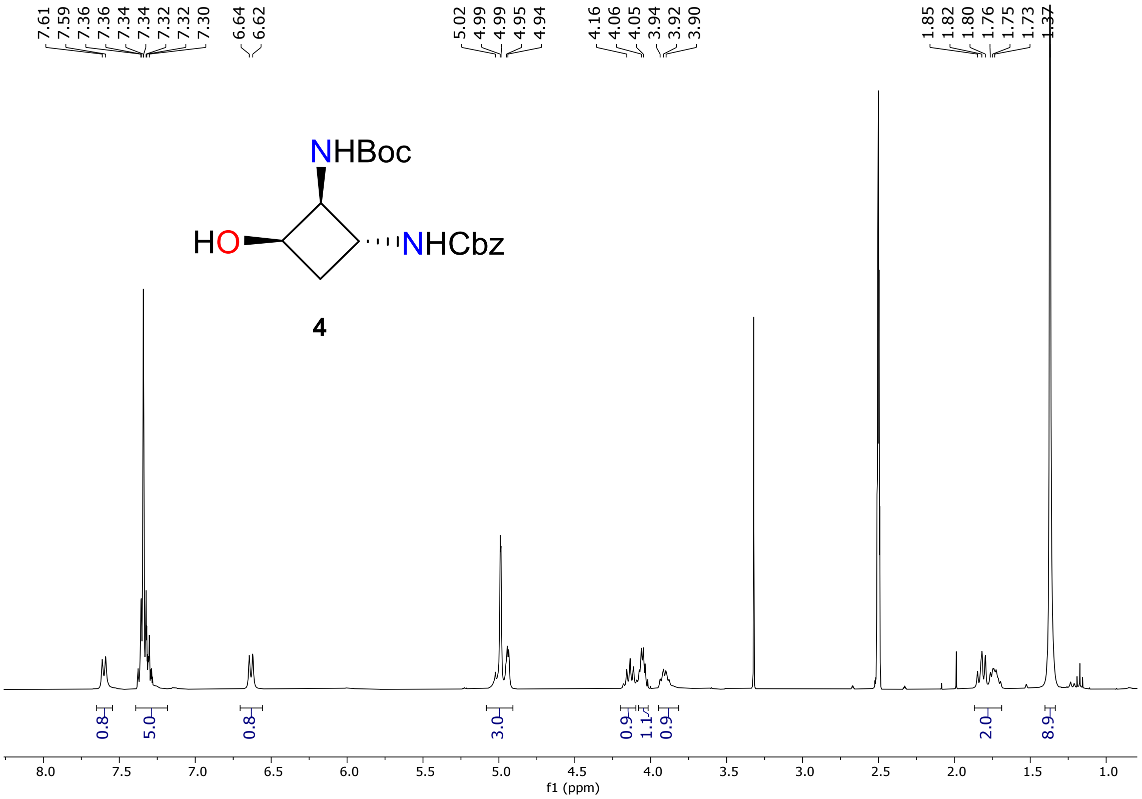

Figure S9. ^{1}H NMR spectrum of compound **4** (400 MHz, DMSO-d_6, 298K).

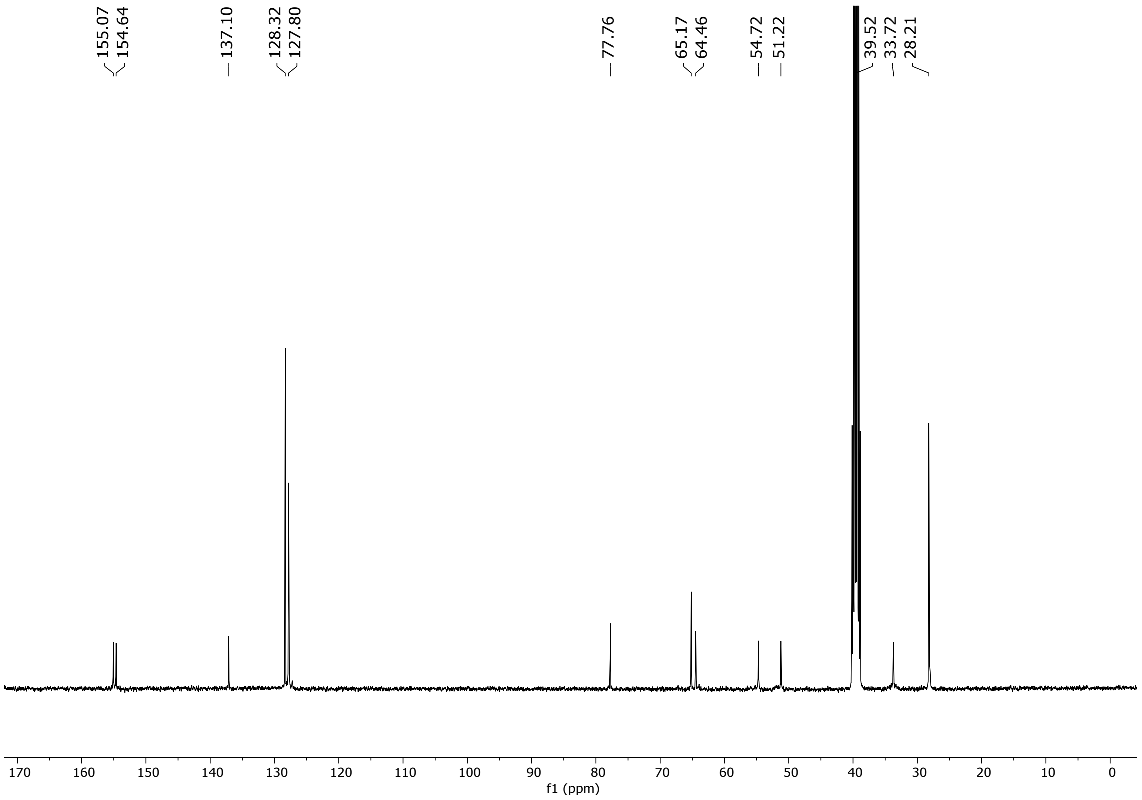

Figure S10. ^{13}C NMR spectrum of compound **4** (100 MHz, DMSO-d_6, 298K).

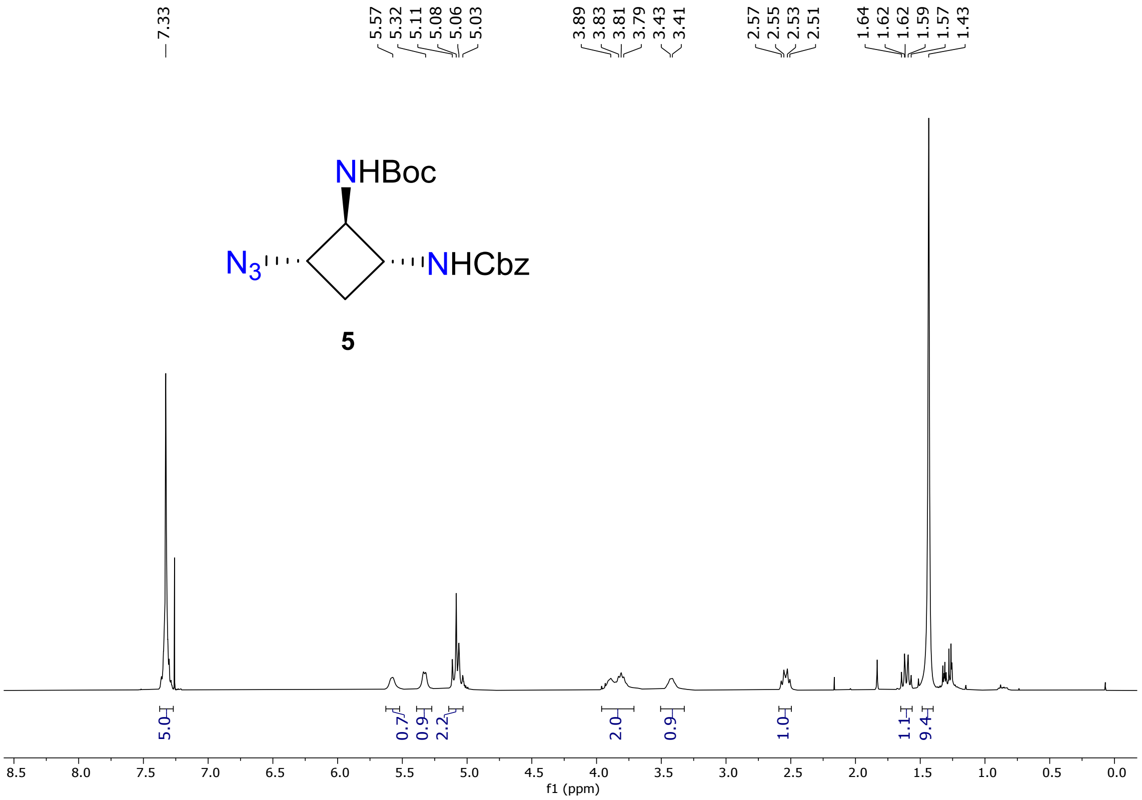

Figure S11. ^{1}H NMR spectrum of compound **5** (400 MHz, $CDCl_3$, 298K).

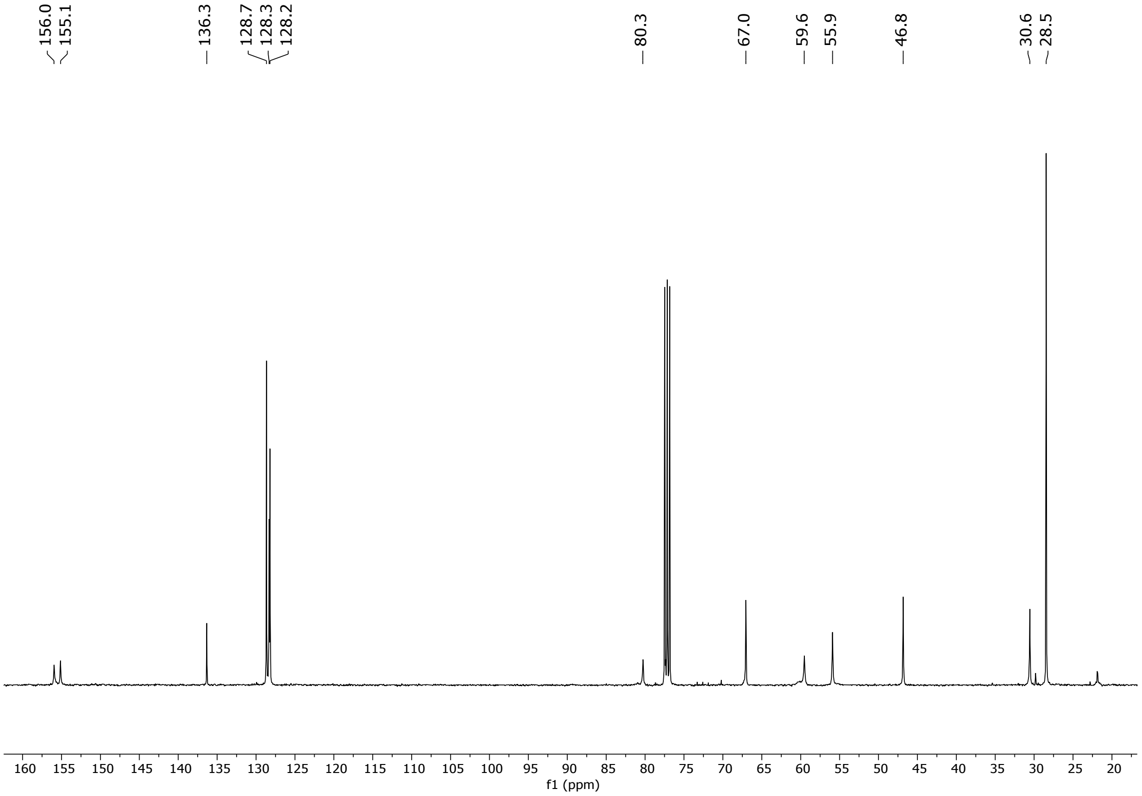

Figure S12. ^{13}C NMR spectrum of compound 5 (100 MHz, $CDCl_3$, 298K).

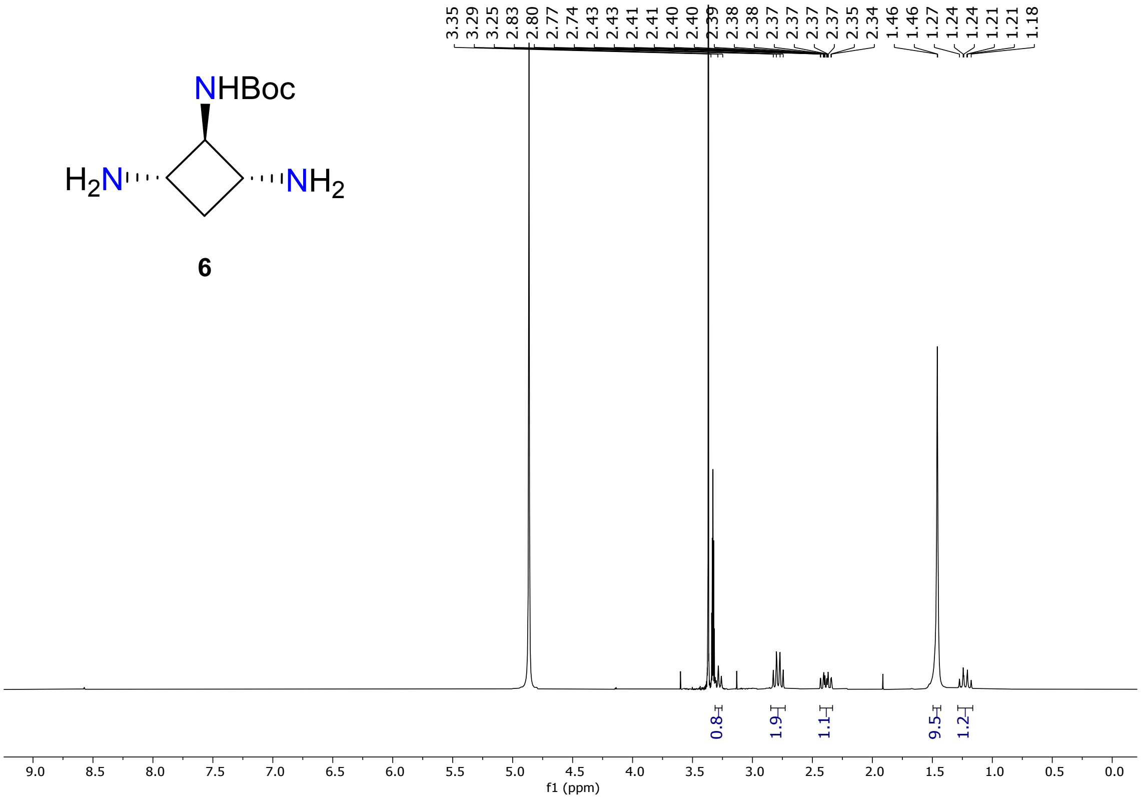

Figure S13. ^{1}H NMR spectrum of compound **6** (300 MHz, MeOD, 298K).

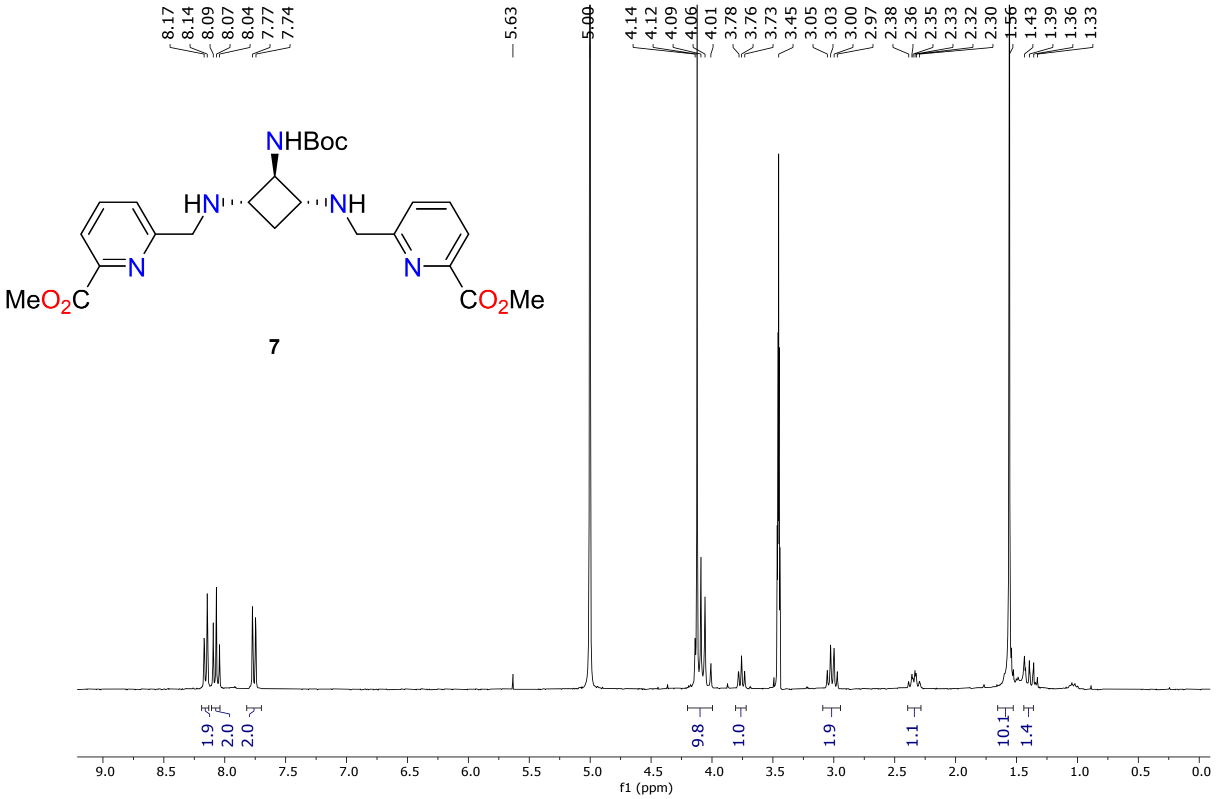

Figure S14. ^{1}H NMR spectrum of compound **7** (300 MHz, MeOD, 298K).

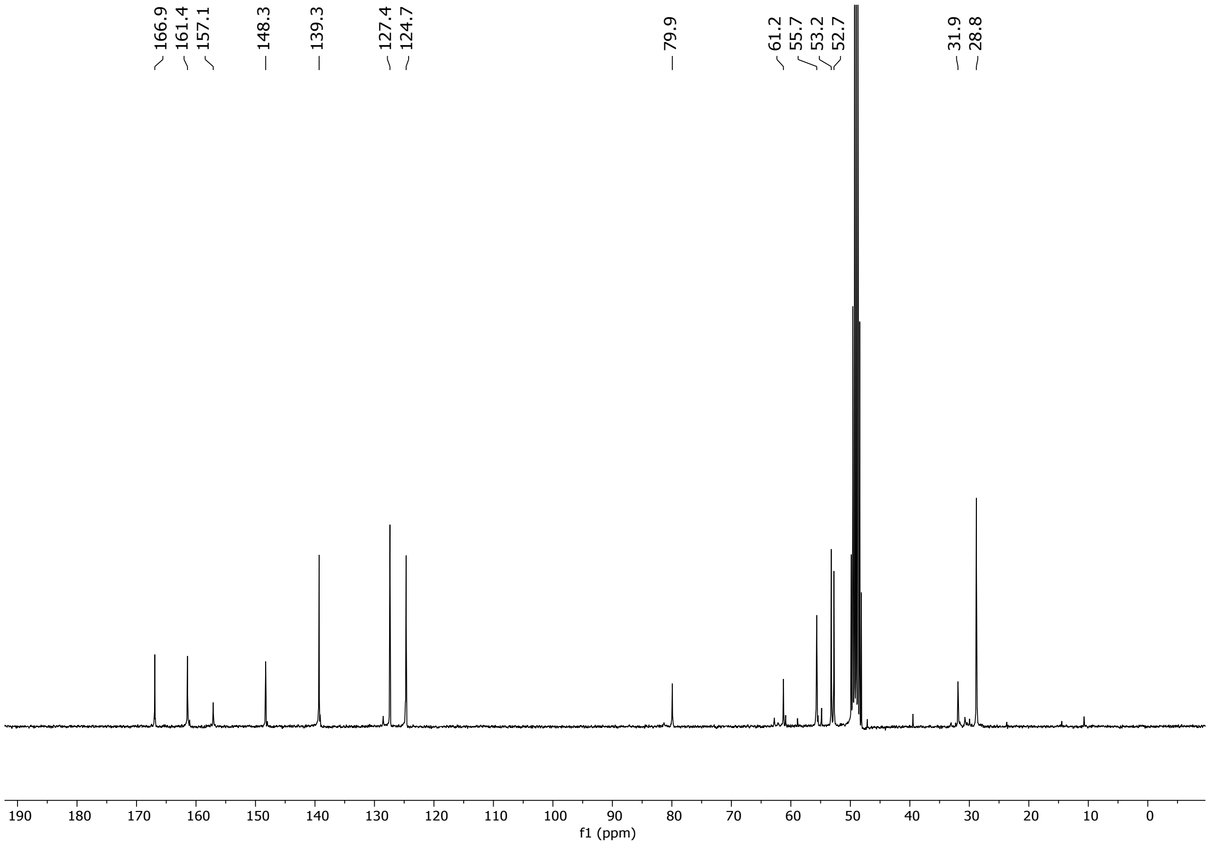

Figure S15. ^{13}C NMR spectrum of compound **7** (100 MHz, MeOD, 298K).

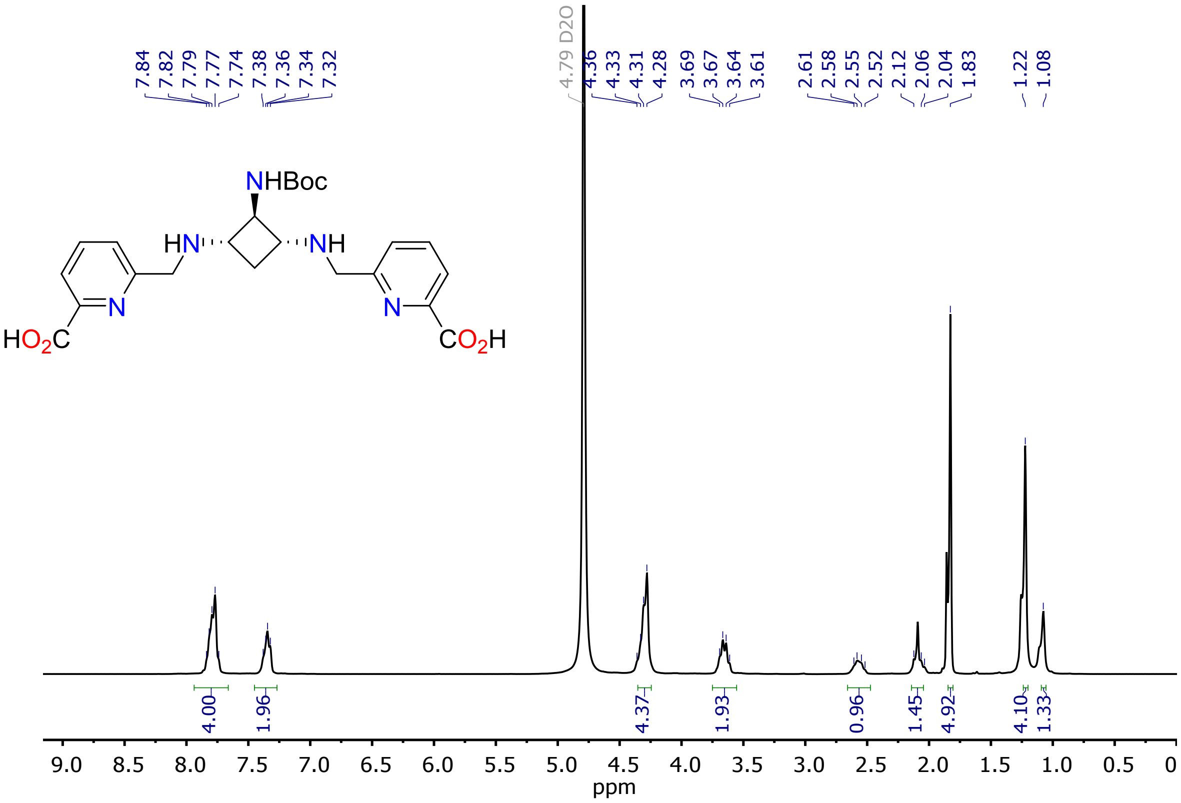

Figure S16. ^{1}H NMR spectrum of compound **H_2CBuDEDPA-NHBoc** (300 MHz, D_2O, pD 6.0, 298K).

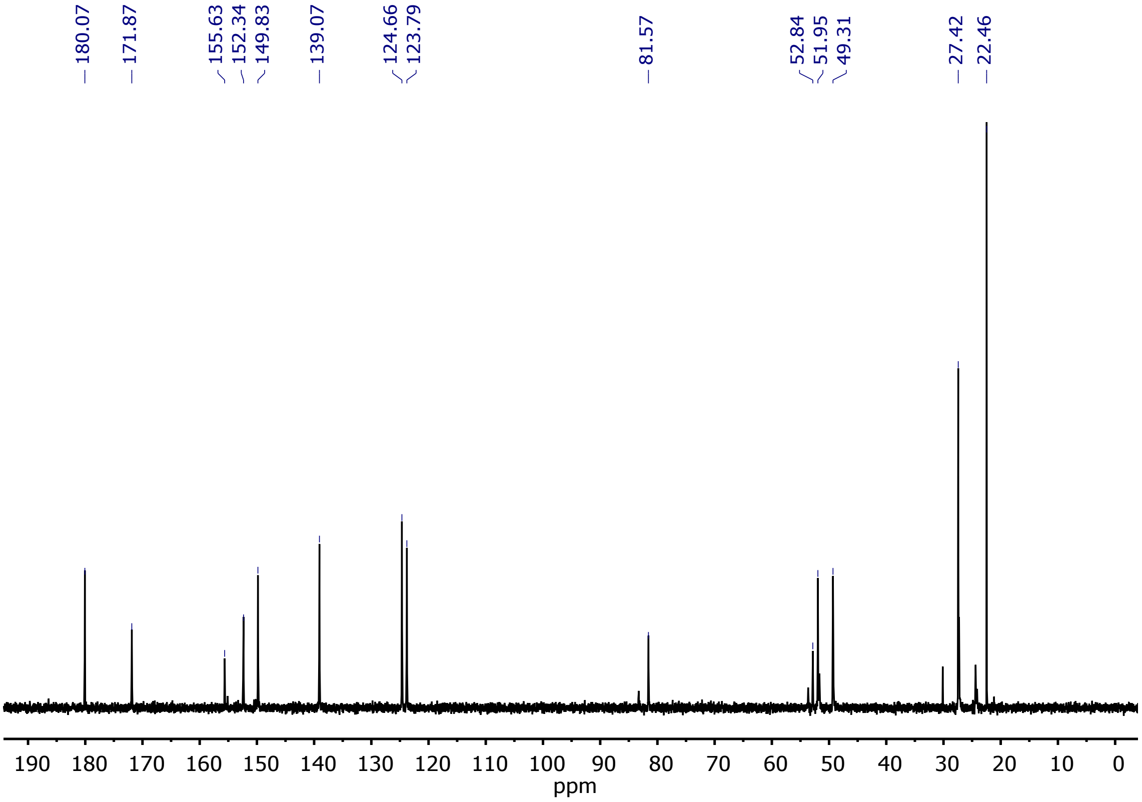

Figure S17. ^{13}C NMR spectrum of compound **H_2CBuDEDPA-NHBoc** (75 MHz, D_2O, pD 6.0, 298K).

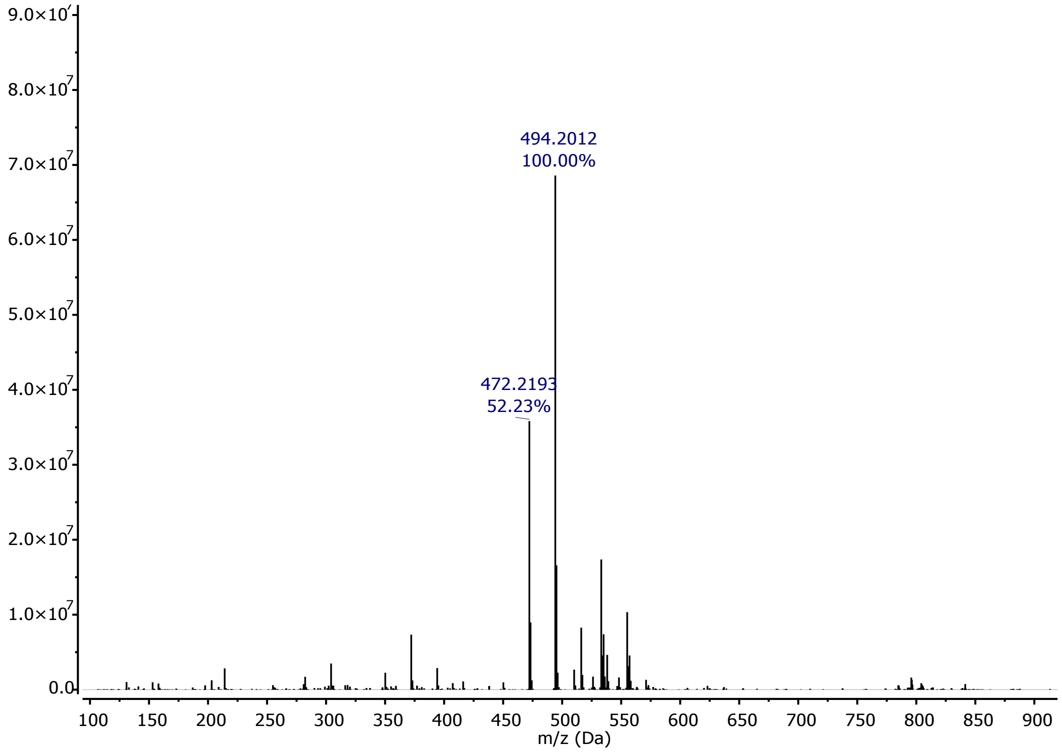

Figure S18. Experimental high resolution mass spectrum (ESI$^+$) of **H_2CBuDEDPA-NHBoc**.

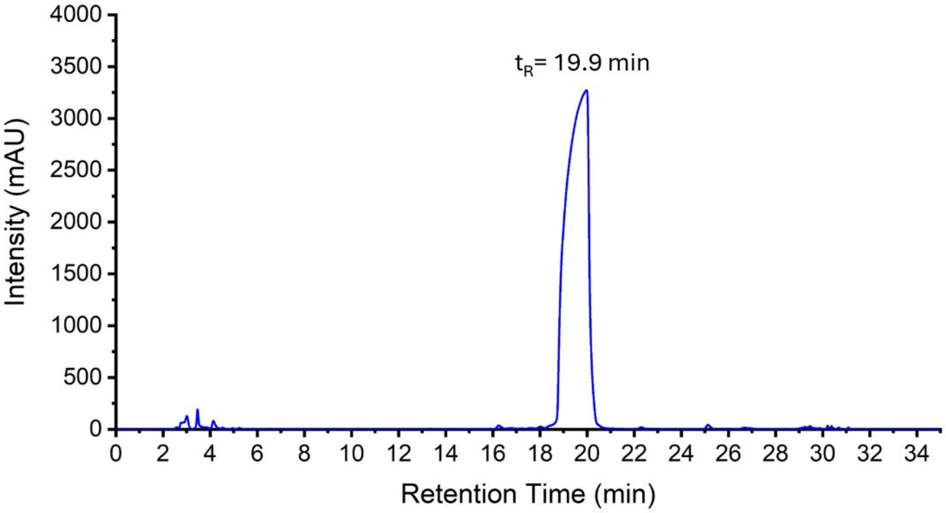

Figure S19. HPLC chromatogram (272 nm) of **H_2CBuDEDPA-NHBoc** at t_R= 19.9 min.

Table S1. MPLC method applied for the purification of **H_2CBuDEDPA-NHBoc** (with mobile phases A= ammonium acetate 10 mM aqueous solution and B= CH_3CN + 10% A).

Column Volumes (CV)	Time (min)	B (%)	Flow (mL/min)
0.00	0.00	0	15
6.00	8.12	0	15
13.50	18.28	12	15
16.30	22.07	12	15
21.00	28.43	20	15
24.00	32.49	100	15
28.00	37.91	100	15

Table S2. HPLC method applied for the purification of **H_2CBuDEDPA-NHBoc** (with mobile phases A= ammonium acetate 10 mM aqueous solution and B= CH_3CN + 10% A).

Time (min)	B (%)	Flow (mL/min)
0	5	20
5	5	20
25	20	20
30	95	20
35	95	20

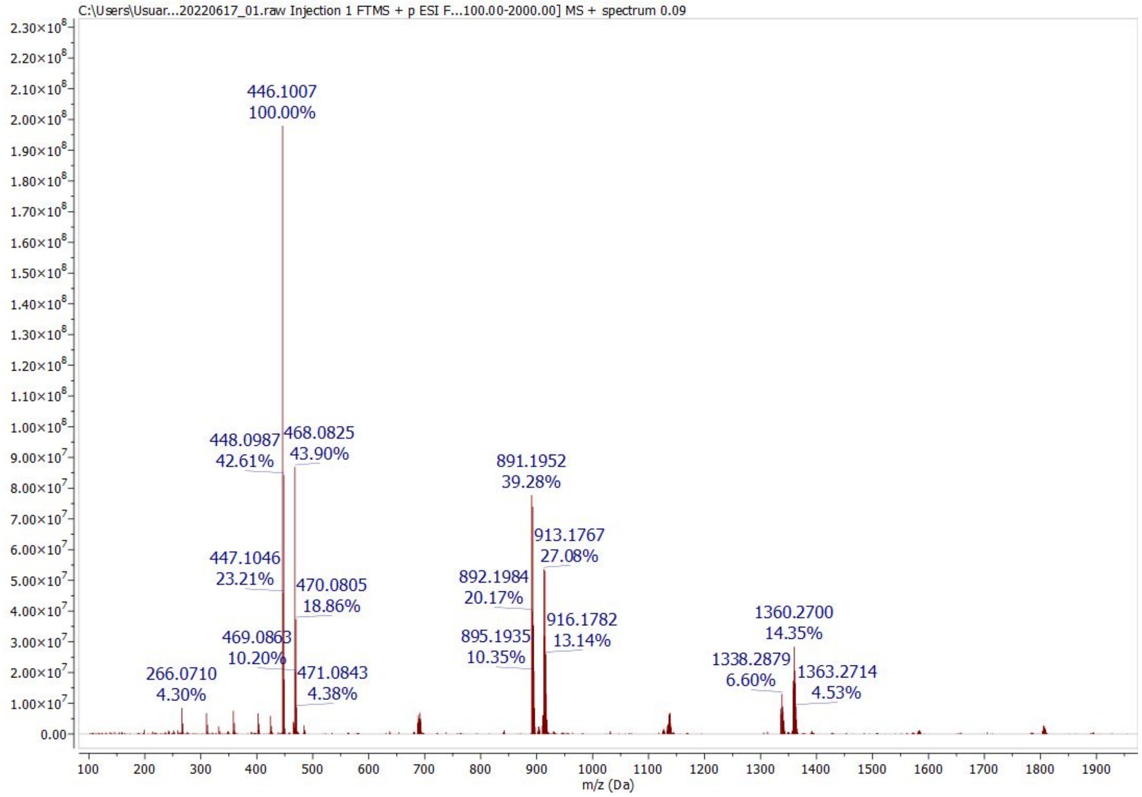

Figure S20. Experimental high resolution mass spectrum (ESI$^+$) of **[Cu(CHXDEDPA)]**.

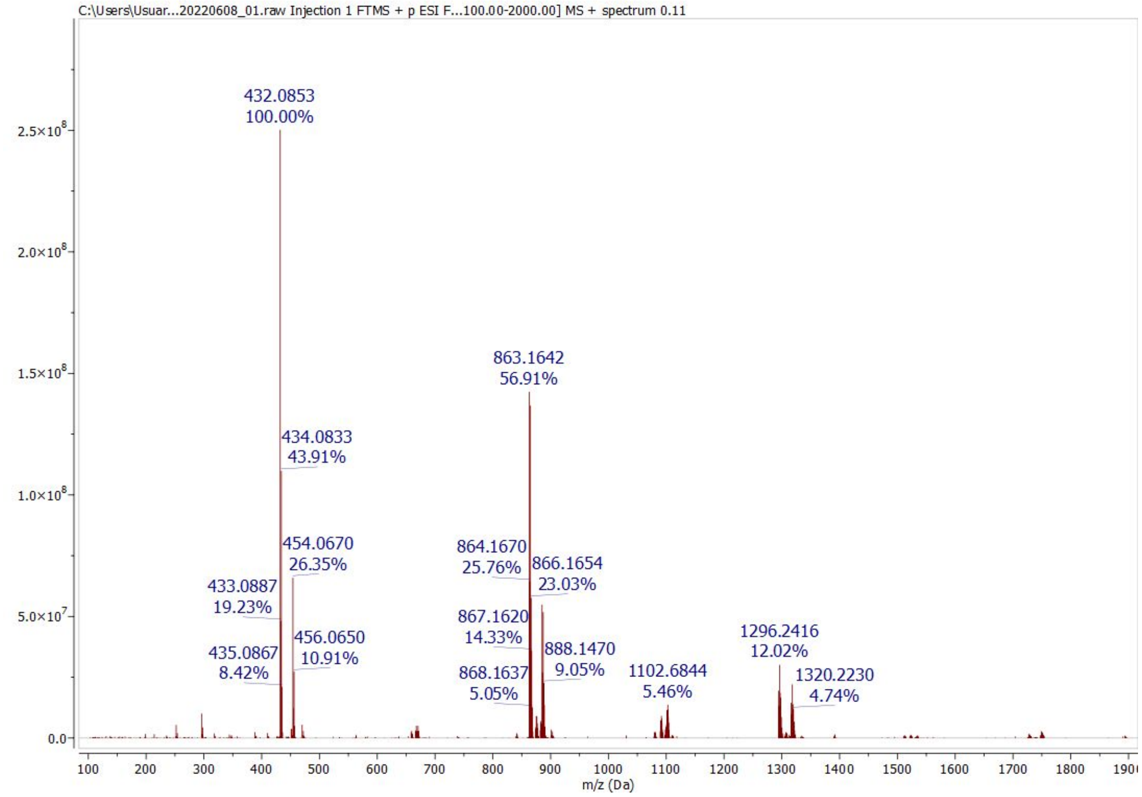

Figure S21. Experimental high resolution mass spectrum (ESI$^+$) of **[Cu(CpDEDPA)]**.

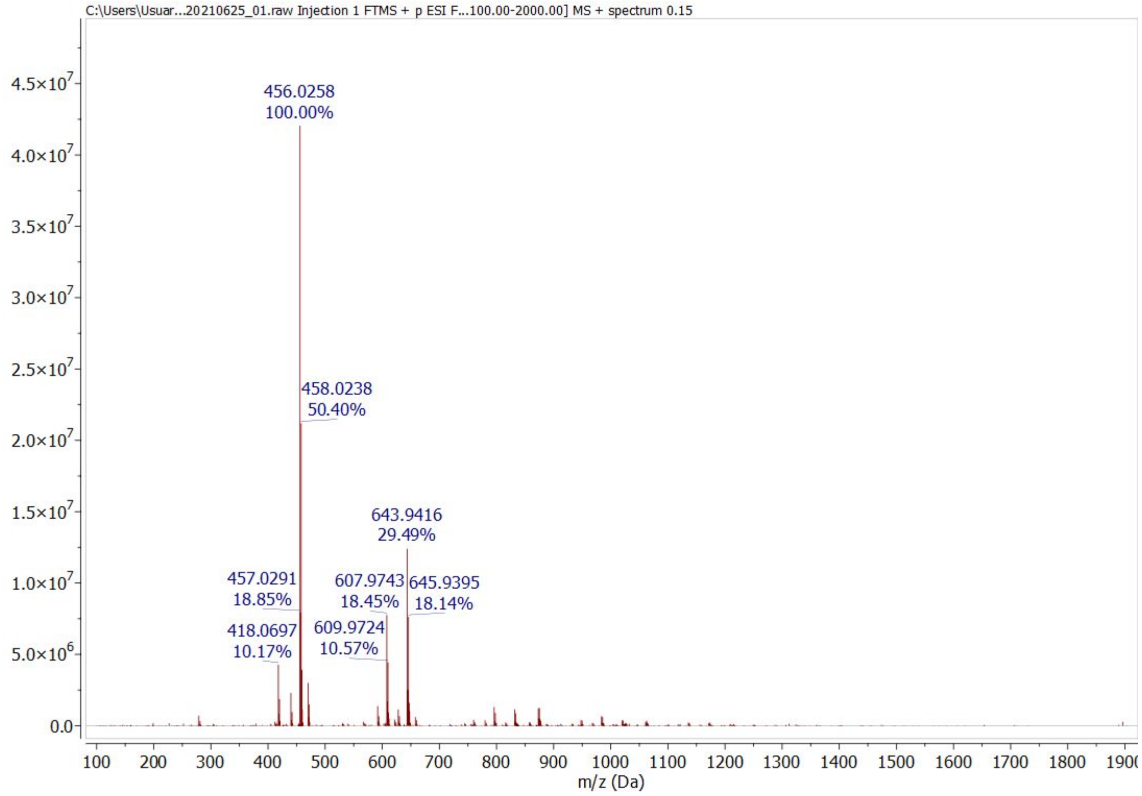

Figure S22. Experimental high resolution mass spectrum (ESI$^+$) of **[Cu(CBuDEDPA)]**.

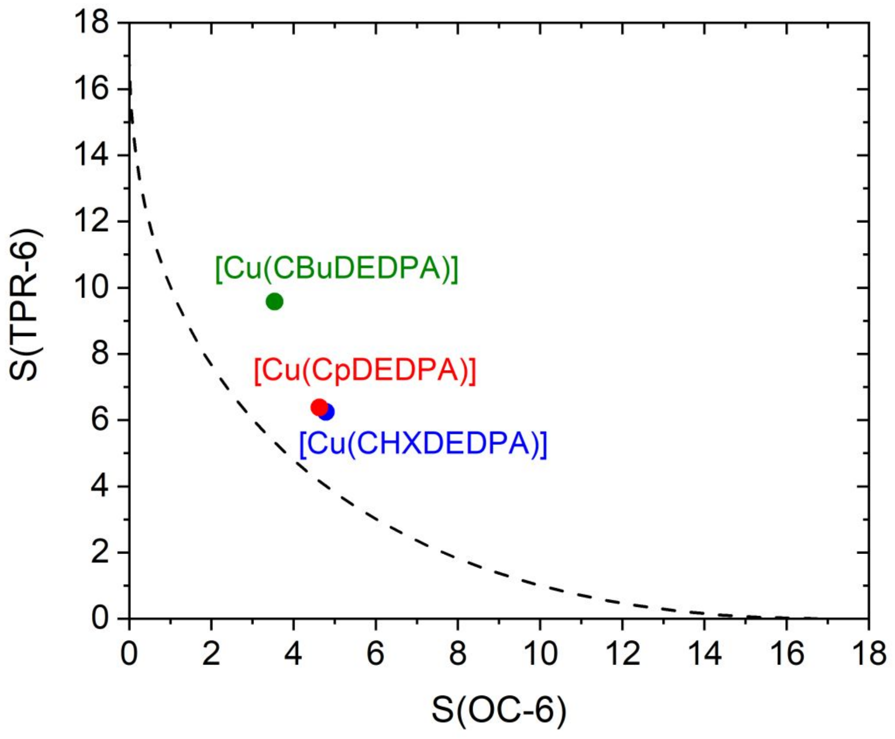

Figure S23. Minimal distortion pathway between an octahedron and a trigonal prism (dashed line) and Shape measures obtained for octahedral [S(OC-6)] and trigonal prismatic [S(TPR-6)] in **[Cu(CBuDEDPA)]**, **[Cu(CpDEDPA)]** and **[Cu(CHXDEDPA)]**.

Table S3. Selected bond angles(º) of the Cu(II) coordination environments in **[Cu(CHXDEDPA)]**, **[Cu(CpDEDPA)]** and **[Cu(CBuDEDPA)]** determined by single-crystal X-ray measurements.

		**[Cu(CHXDEDPA)]**	**[Cu(CpDEDPA)]**	**[Cu(CBuDEDPA)]**
cis donors in equatorial plane (ideal value 90º)	N(1)-Cu(1)-N(2)	80.1	80.1	78.1
	N(2)-Cu(1)-N(4)	105.2	107.2	113.2
	N(4)-Cu(1)-O(1)	95.4	93.7	93.1
	O(1)-Cu(1)-N(1)	79.5	80.9	77.1
cis donors in equatorial-axial relative position (ideal value 90º)	N(1)-Cu(1)-O(3)	94.2	93.5	96.7
	N(1)-Cu(1)-N(3)	112.2	113.0	105.6
	N(2)-Cu(1)-O(3)	92.1	87.5	93.1
	N(2)-Cu(1)-N(3)	91.7	83.5	80.7
	N(4)-Cu(1)-O(3)	75.9	77.8	80.1
	N(4)-Cu(1)-N(3)	78.0	77.6	79.9
	O(1)-Cu(1)-O(3)	92.7	106.2	107.2
	O(1)-Cu(1)-N(3)	93.0	92.5	89.3
Donors in axial-axial relative position (ideal value 180º)	O(1)-Cu(1)-N(2)	159.3	157.2	149.4
	N(4)-Cu(1)-N(1)	168.7	168.1	168.3
	O(3)-Cu(1)-N(3)	153.7	149.8	154.6

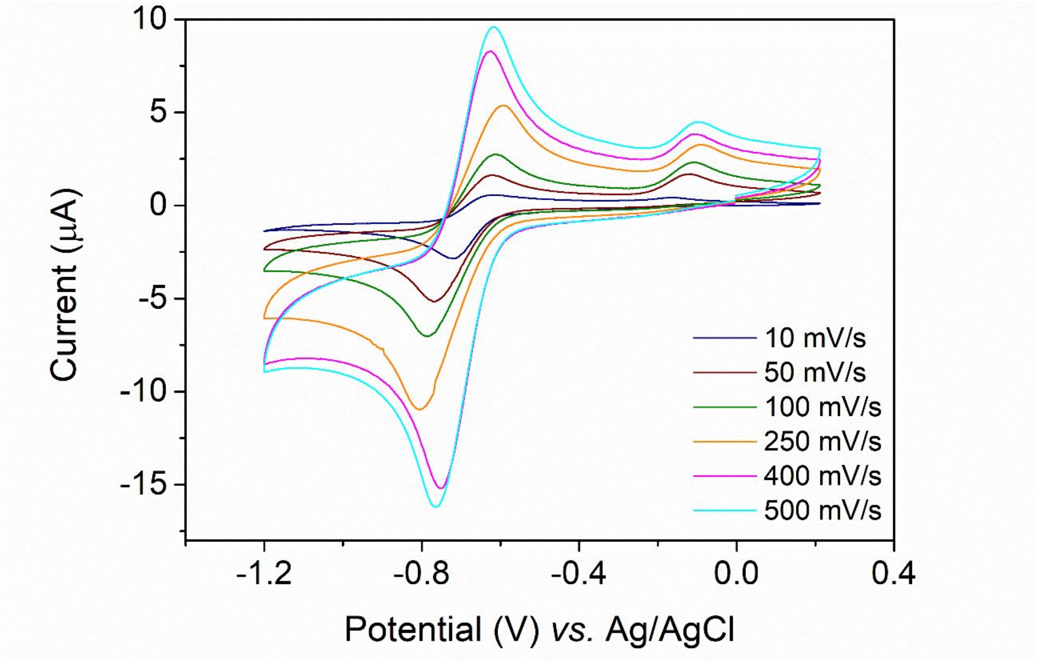

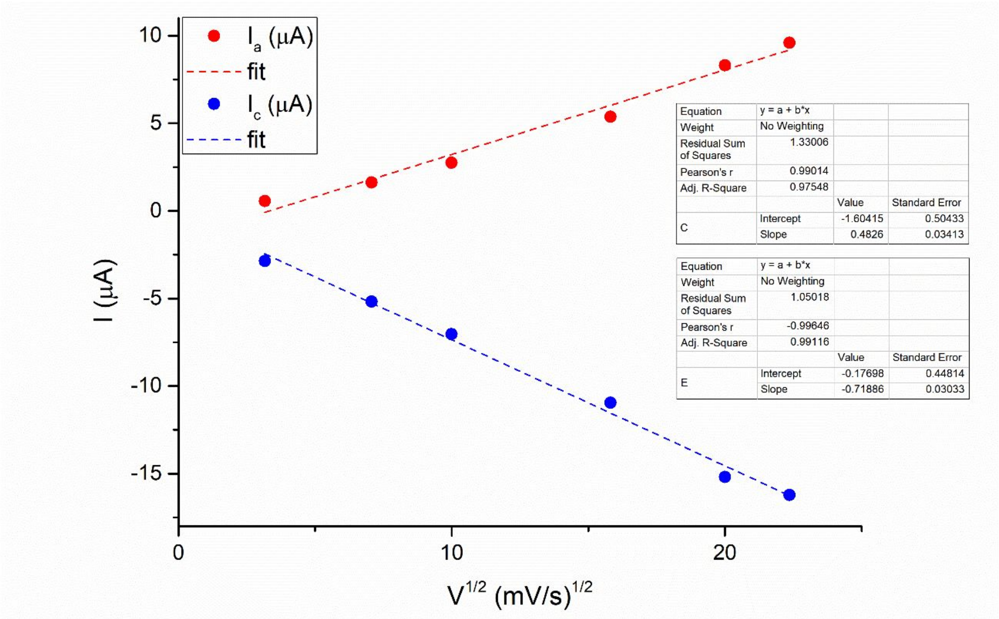

Figure S24. Cyclic voltammogram of **[Cu(CHXDEDPA)]** complex in aqueous solution in 0.15 M NaCl (1.4 mM, pH= 6.6), recorded at 10, 50, 100, 250, 400 and 500 mV·s^{-1} (top); and plots of the linear dependence of anodic and cathodic peak currents with the square root of the scan rate (bottom).

**[Cu(CHXDEDPA)]** (mV/s)	E_a (V)	I_a (μA)	E_c (V)	I_c (μA)	$E_{1/2}$ (V)	Ea-Ec (V)	$E_{1/2}$ (V)
10	-0.62027	0.56305	-0.72037	-2.8537	-0.67032	0.1001	-0.45032
50	-0.62027	1.62781	-0.7692	-5.17578	-0.694735	0.14893	-0.474735
100	-0.61295	2.74658	-0.78629	-7.0343	-0.69962	0.17334	-0.47962
250	-0.59097	5.37415	-0.80338	-10.95	-0.697175	0.21241	-0.477175
400	-0.62515	8.30078	-0.75211	-15.2	-0.68863	0.12696	-0.46863
500	-0.62027	9.60083	-0.76431	-16.211	-0.69229	0.14404	-0.47229

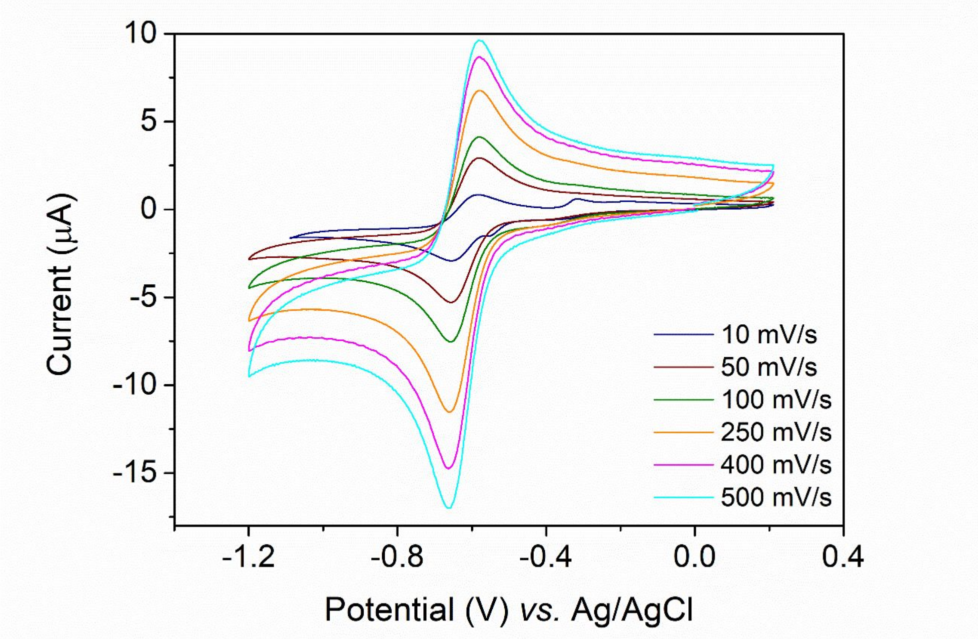

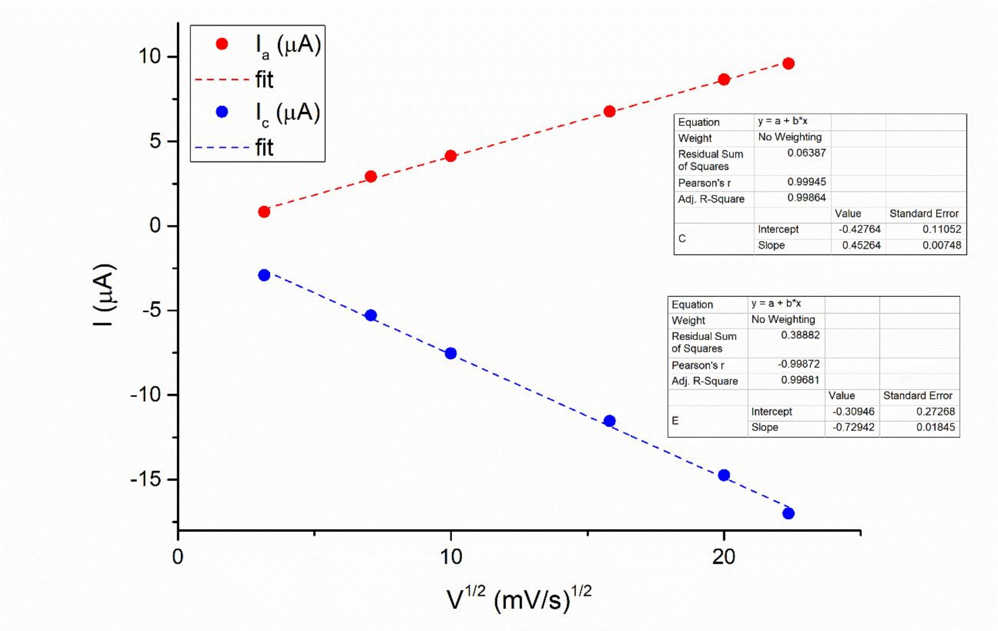

Figure S25. Cyclic voltammogram of **[Cu(CpDEDPA)]** complex in aqueous solution in 0.15 M NaCl (1.3 mM, pH= 5.9), recorded at 10, 50, 100, 250, 400 and 500 mV·s^{-1} (top); and plots of the linear dependence of anodic and cathodic peak currents with the square root of the scan rate (bottom).

**[Cu(CpDEDPA)]** (mV/s)	E_a (V)	I_a (μA)	E_c (V)	I_c (μA)	$E_{1/2}$ (V)	Ea-Ec (V)	$E_{1/2}$ (V)
10	-0.58365	0.83923	-0.65445	-2.91595	-0.61905	0.0708	-0.399
50	-0.58121	2.92999	-0.65445	-5.2887	-0.61783	0.07324	-0.398
100	-0.57877	4.133	-0.65933	-7.547	-0.61905	0.08056	-0.399
250	-0.57877	6.77185	-0.65933	-11.5417	-0.61905	0.08056	-0.399
400	-0.57632	8.64868	-0.66177	-14.7461	-0.619045	0.08545	-0.399
500	-0.57388	9.60083	-0.66422	-17.0074	-0.61905	0.09034	-0.399

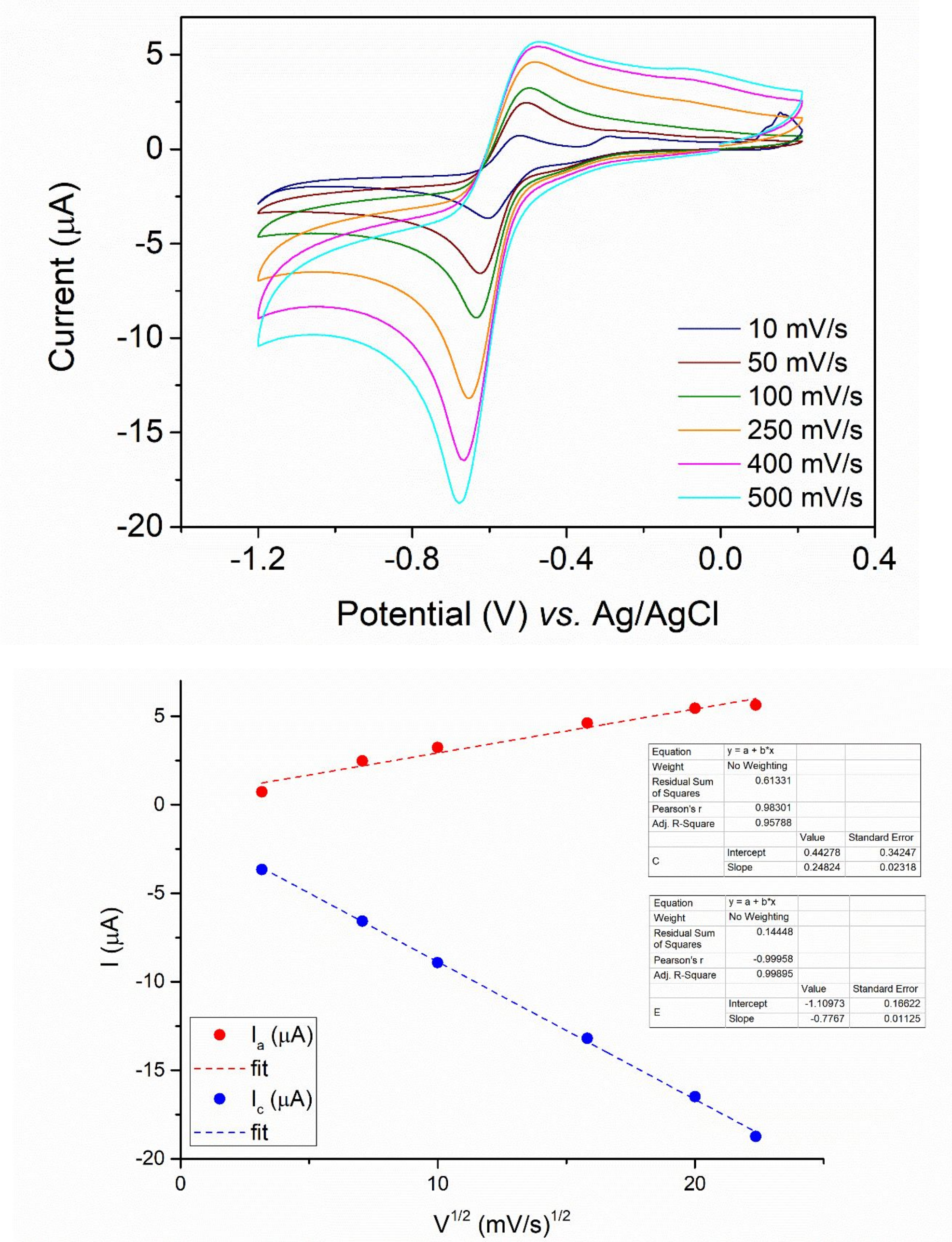

Figure S26. Cyclic voltammogram of [Cu(CBuDEDPA)] complex in aqueous solution in 0.15 M NaCl (1.3 mM, pH= 5.0), recorded at 10, 50, 100, 250, 400 and 500 mV·s^{-1} (top); and plots of the linear dependence of anodic and cathodic peak currents with the square root of the scan rate (bottom).

**[Cu(CBuDEDPA)]** (mV/s)	E_a (V)	I_a (μA)	E_c (V)	I_c (μA)	$E_{1/2}$ (V)	Ea-Ec (V)	$E_{1/2}$ (V)
10	-0.51161	0.72449	-0.60562	-3.65265	-0.558615	0.09401	-0.339
50	-0.50552	2.46613	-0.62515	-6.57043	-0.565335	0.11963	-0.345335
100	-0.49332	3.23822	-0.63248	-8.91724	-0.5629	0.13916	-0.3429
250	-0.48843	4.61426	-0.65201	-13.1897	-0.57022	0.16358	-0.35022
400	-0.47623	5.44128	-0.66666	-16.4856	-0.571445	0.19043	-0.351445
500	-0.47134	5.63542	-0.67886	-18.74	-0.5751	0.20752	-0.3551

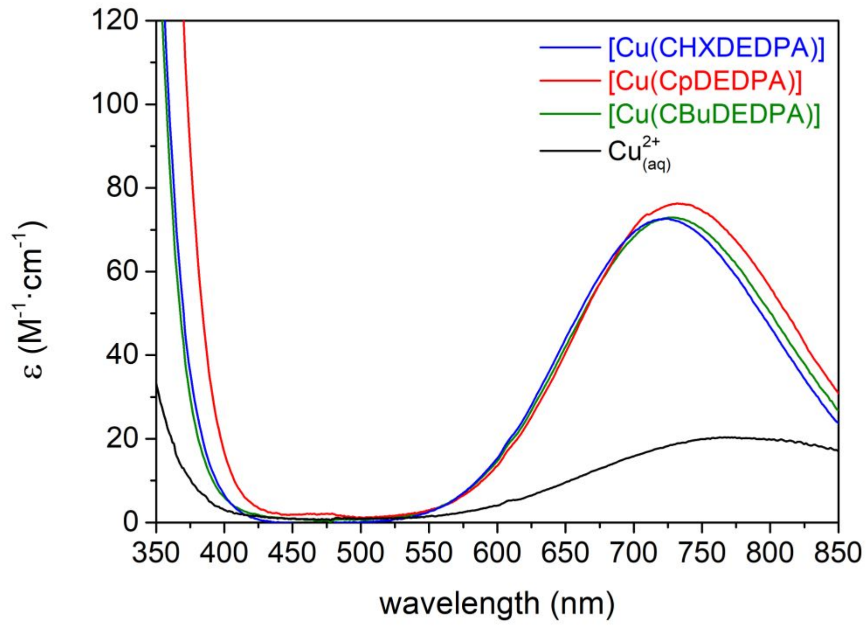

Figure S27. Spectra of **[Cu(CHXDEDPA)]** (1.4 mM, blue curve), **[Cu(CpDEDPA)]** (1.3 mM, red curve), **[Cu(CBuDEDPA)]** (1.3 mM, green curve) and $CuCl_2$ (1.3 mM, black curve) dissolved in water at 298 K.

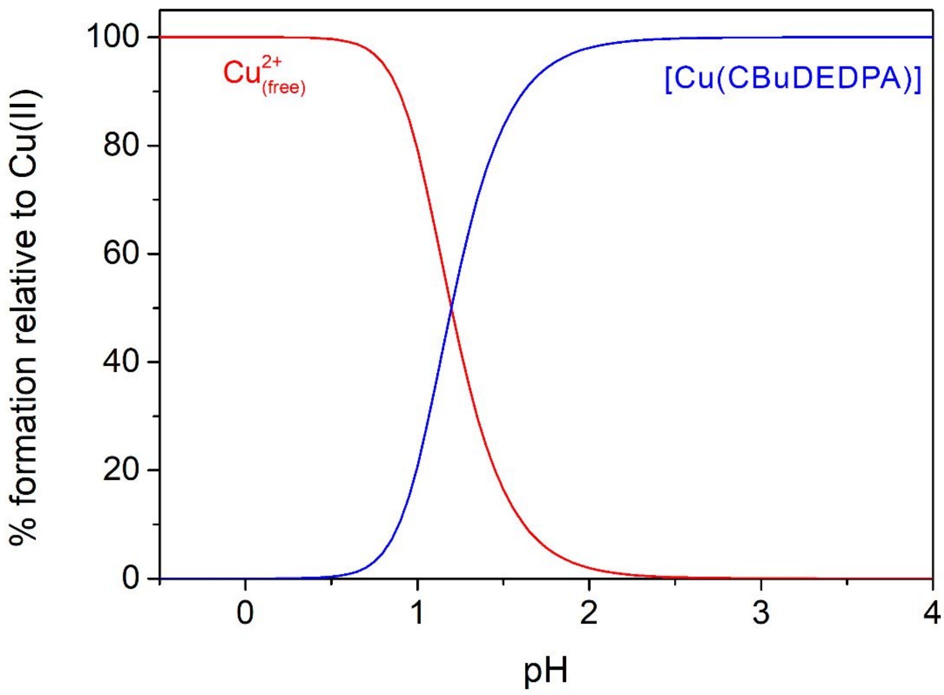

Figure S28. Species diagram calculated for **[Cu(CBuDEDPA)]** complex ([L]= [Cu^{2+}]= 10^{-3} M).

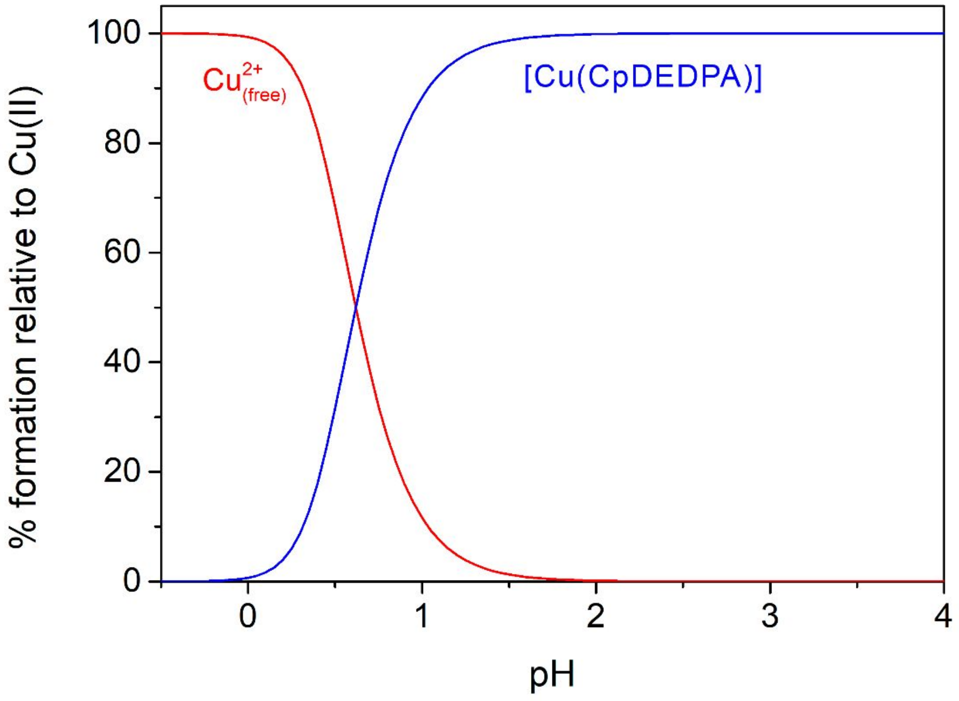

Figure S29. Species diagram calculated for **[Cu(CpDEDPA)]** complex ([L]= [Cu^{2+}]= 10^{-3} M).

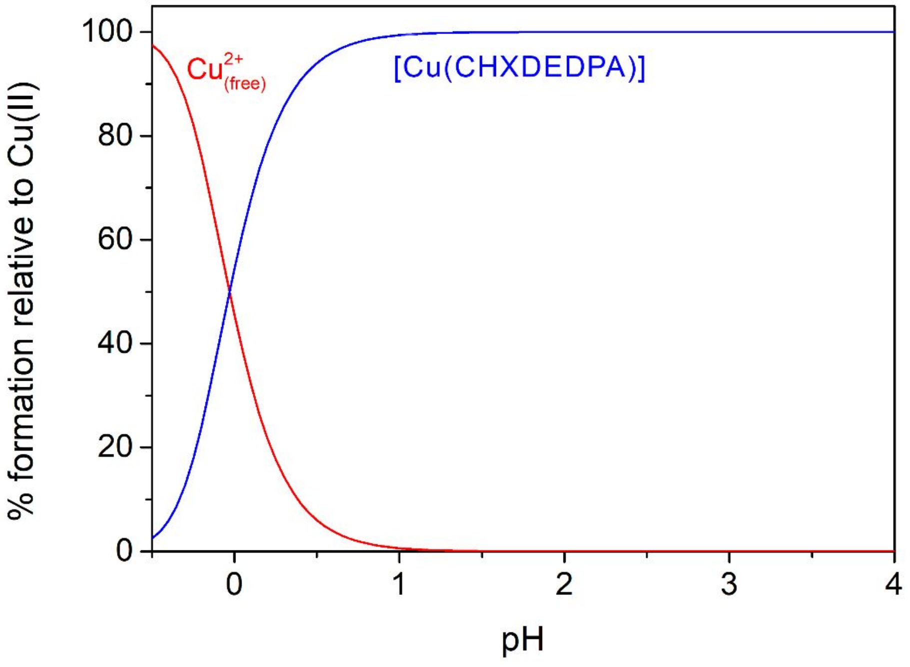

Figure S30. Species diagram calculated for **[Cu(CHXDEDPA)]** complex ([L]= [Cu2+]= 10-3 M).

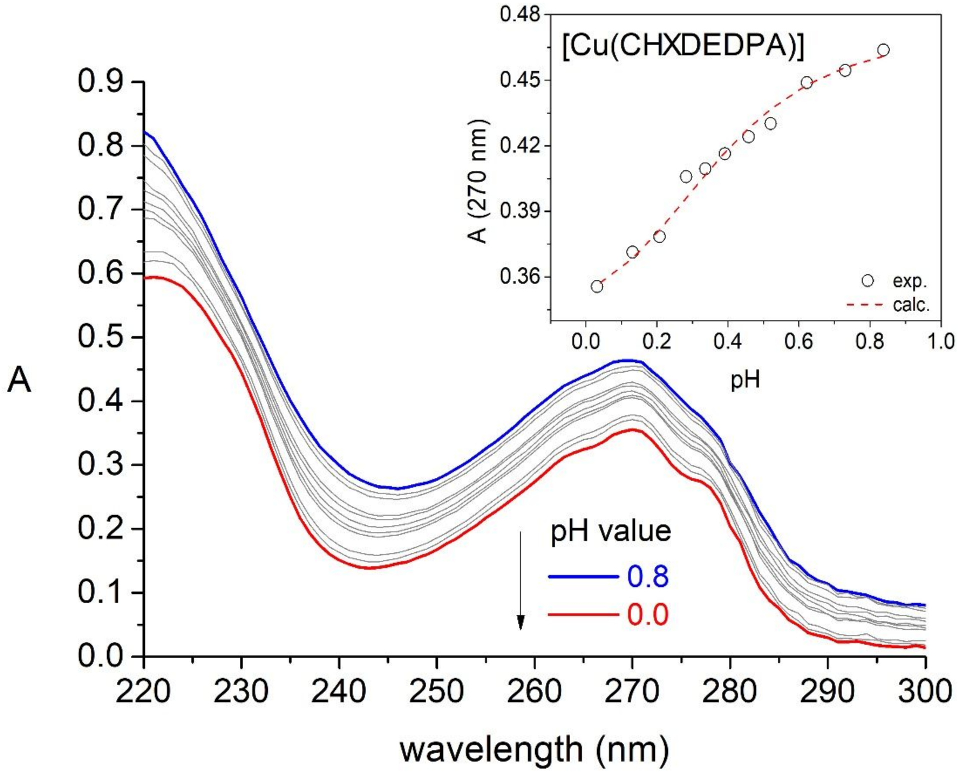

Figure S31. Spectrophotometric titration of **[Cu(CHXDEDPA)]** (3.68×10^{-5}, I = 1 M NaCl) with pH. The inset shows the experimental absorbance values at 270 nm and the dashed line the fit of the data for stability constant determination.

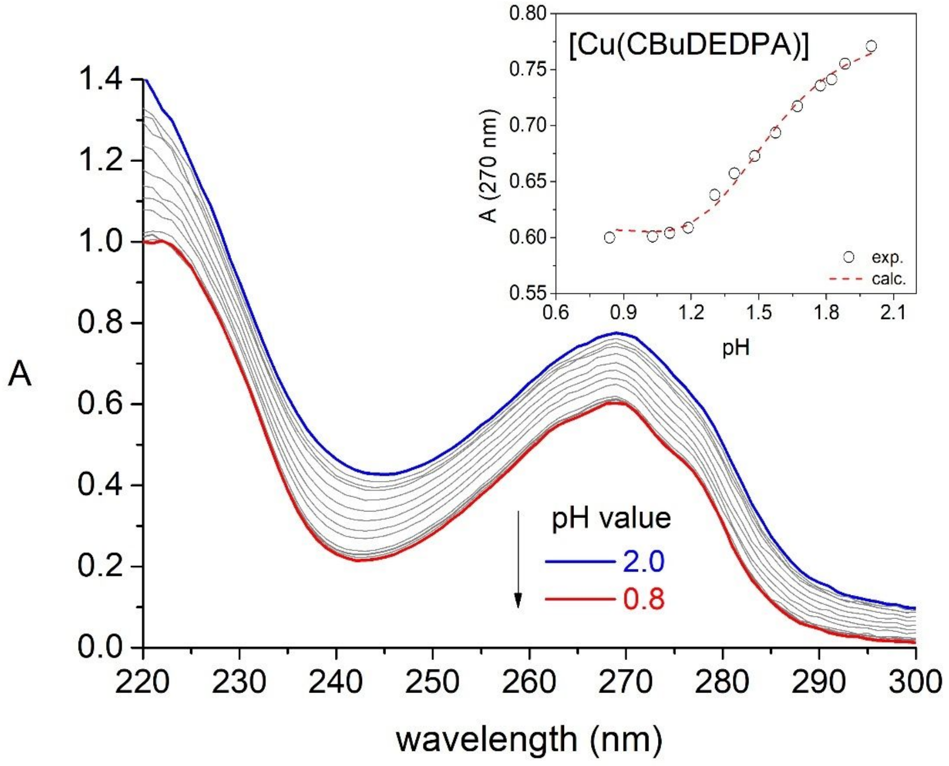

Figure S32. Spectrophotometric titration of **[Cu(CBuDEDPA)]** (6.68×10^{-5}, I = 1 M NaCl) with pH. The inset shows the experimental absorbance values at 270 nm and the dashed line the fit of the data for stability constant determination.

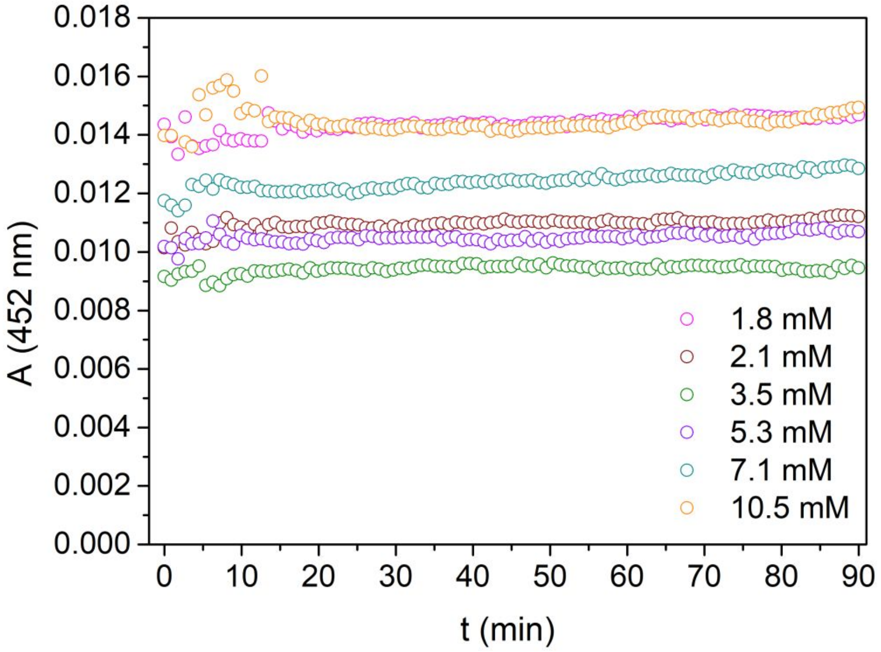

Figure S3. Dependence of absorbance versus [AA] for **[Cu(CHXDEDPA)]** (84 μM); [NC]= 0.20 mM; [buffer]= 0.18 M and pH 6.7 recorded at 298 K.

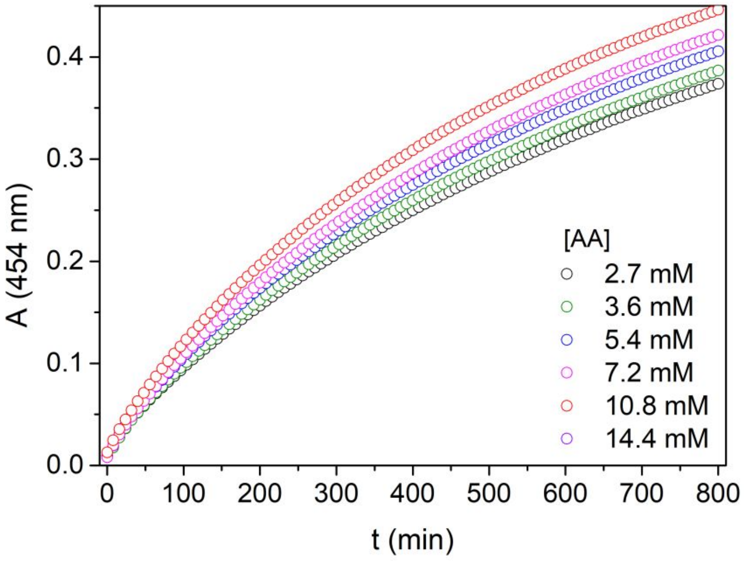

Figure S284. Variation of the absorption at selected wavelength versus [AA] for **[Cu(CBuDEDPA)]** (91 μM); [NC]= 0.24 mM; [buffer]= 0.12 M; pH 6.3 recorded at 298 K.

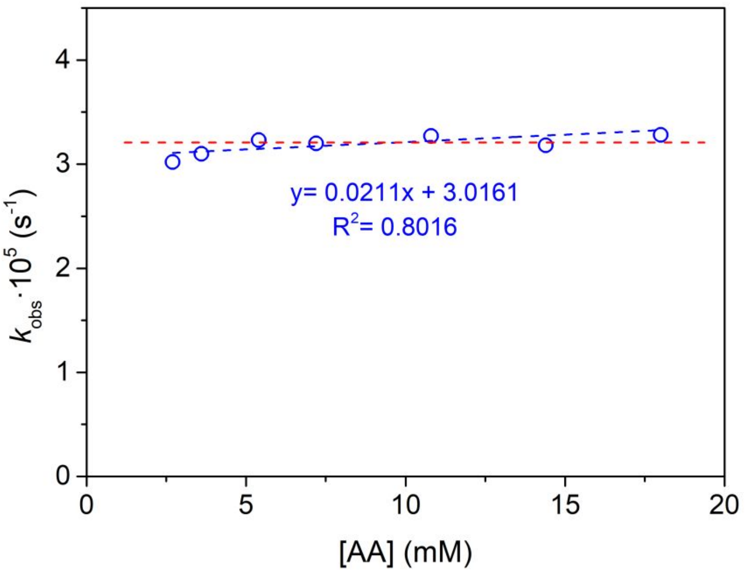

Figure S295. Dependence of k_{obs} versus [AA] for **[Cu(CBuDEDPA)]** (91 μM); [NC]= 0.24 mM; [buffer]= 0.12 M and pH 6.3 recorded at 298 K.

Table S4. Parameters obtained when studying the influence of [AA] concentration for **[Cu(CBuDEDPA)]** (91 μM); ([NC]=0.24 mM; buffer $HPO_4^{-2}/H_2PO_4^-$ 0.122 M, pH 6.3; I= 0.15 M).

**[AA]/mM**	k_{obs}**/10^{-5} s^{-1}**	**A$_\infty$**
2.7	3.02	0.4807
3.6	3.10	0.4919
5.4	3.23	0.5071
7.2	3.20	0.5311
10.8	3.27	0.5112
14.4	3.18	0.5891
18.0	3.28	0.5121

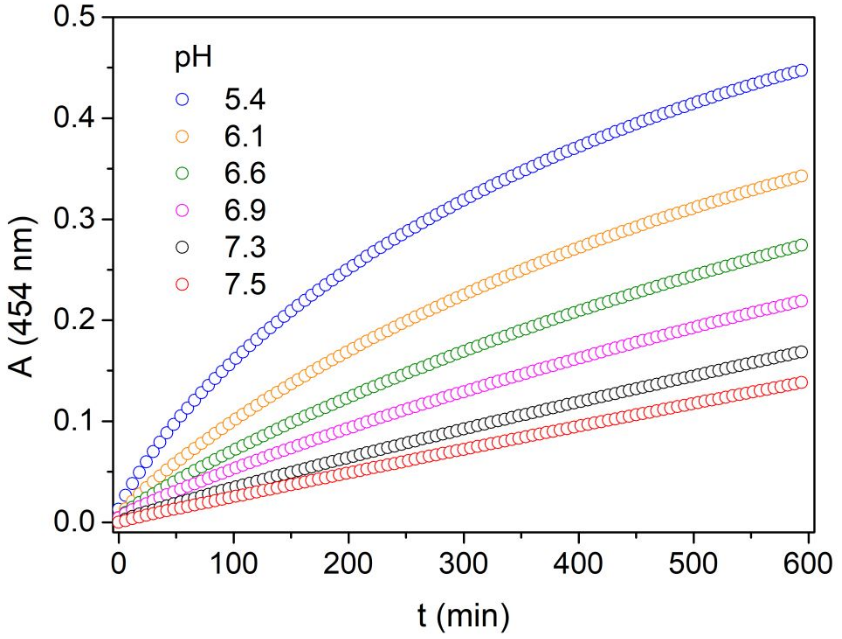

Figure S306. Variation of the absorption at selected wavelength versus [pH] for **[Cu(CBuDEDPA)]** (91 μM); [NC]= 0.24 mM; [buffer]= 0.12 M and [AA]= 5.4 mM recorded at 298 K.

Table S5. Parameters obtained when studying the influence of pH for **[Cu(CBuDEDPA)]** (91 μM); ([NC]=0.24 mM; [5.4 mM; buffer $HPO_4^{-2}/H_2PO_4^{-}$ 0.122 M; I= 0.15 M) recorded at 298 K.

**pH**	$k_{obs}/10^{-5}$ s^{-1}	$[H^+]/10^{-6}M$	$[H^+]10^{-6}/10^{-5}k_{obs}$	A_∞
5.37	5.78	4.26579	0.73802	0.5215
5.57	5.08	2.6915	0.52982	0.4764
5.91	4.15	1.2302	0.29645	0.5182
6.18	3.63	0.66069	0.18201	0.4982
6.41	2.83	0.38904	0.13747	0.4802
6.65	2.32	0.22387	0.0965	0.5082
6.92	1.87	0.12022	0.06429	0.5451
7.19	1.285	0.06456	0.05024	0.5315
7.28	1.21	0.05248	0.04337	0.5151
7.429	0.936	0.03723	0.03978	0.5092

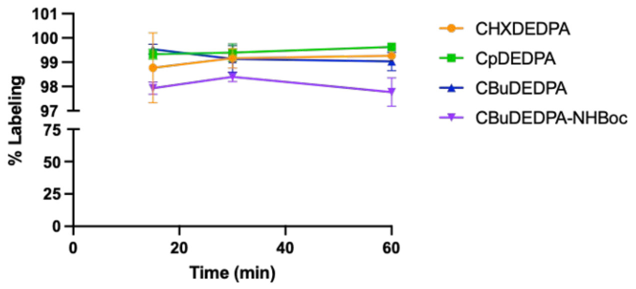

Figure S37. Labeling efficiency for 10 nmol of ligand (n=3).

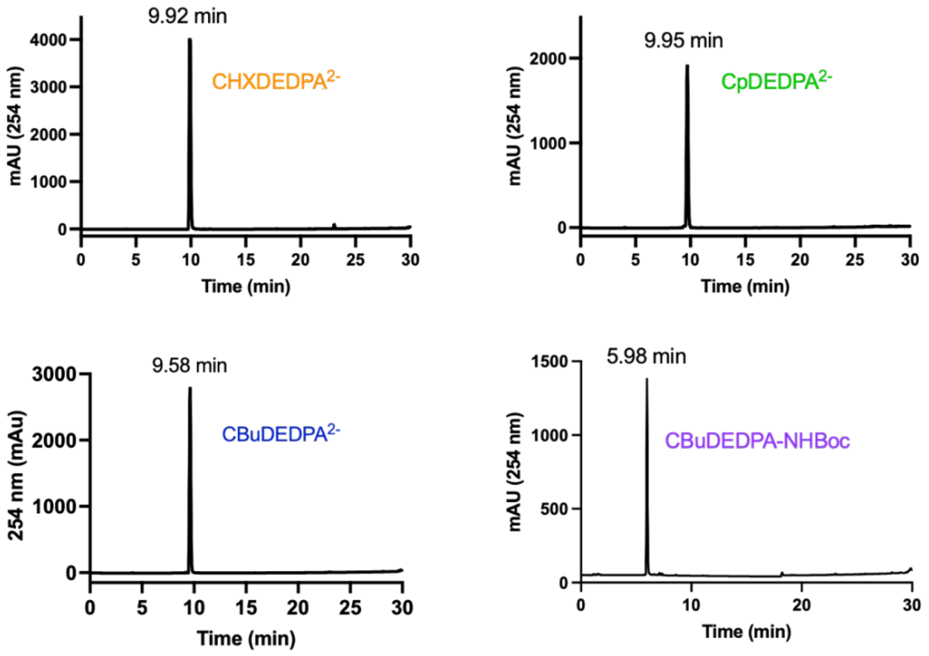

Figure S318. HPLC traces of ligands. For **CHXDEDPA**, **CpDEDPA** and **CBuDEDPA** (Method A: (A) 0.1% TFA in water and (B) 0.1% TFA in CH_3CN). For **CBuDEDPA-NHBoc** (Method B: (A) 10 mM NH_4OAc pH 7 (B) CH_3CN). Gradient: 0-2 min: 5% B. 2-24 min: 5-95%B. 24-26 min: 95%B. 26-28 min: 95-5%B. 28-30 min: 5%B, UV detection at 220 and 254 nm.

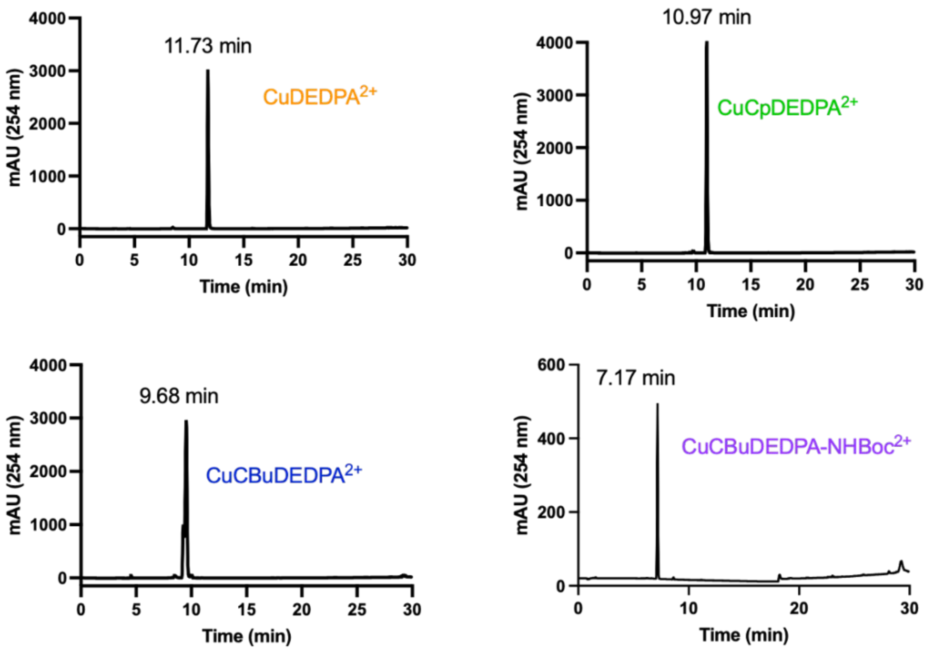

Figure S32. HPLC traces of $^{nat}Cu^{2+}$ complexes. Complexation in MeOH/H_2O (pH 5, 15 min). For **CHXDEDPA**, **CpDEDPA** and **CBuDEDPA** (Method A: (A) 0.1% TFA in water and (B) 0.1% TFA in CH_3CN). For **CBuDEDPA-NHBoc** (Method B: (A) 10 mM NH_4OAc pH 7 (B) CH_3CN). Gradient: 0-2 min: 5% B. 2-24 min: 5-95%B. 24-26 min: 95%B. 26-28 min: 95-5%B. 28-30 min: 5%B, UV detection at 220 and 254 nm.

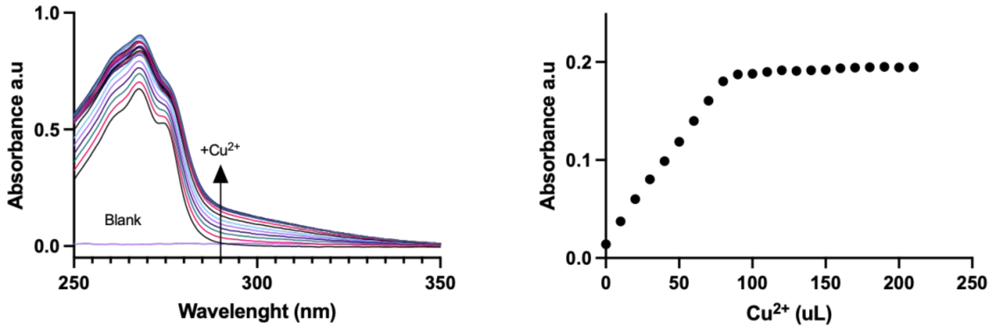

Figure S40. Spectrophotometric titration of **CHXDEDPA** with Cu^{2+}. UV-Vis absorbance spectra of **CHXDEDPA** upon Cu^{2+} addition (left) and UV-vis titration to endpoint to determine ligand concentration (right).

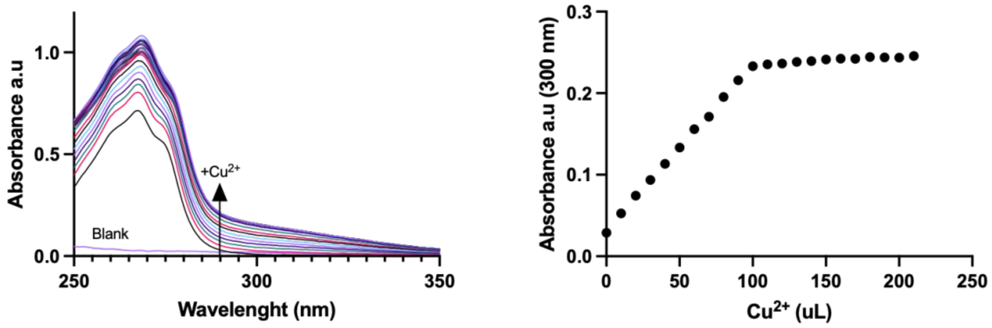

Figure S33. Spectrophotometric titration of **CpDEDPA** with Cu^{2+}. UV-Vis absorbance spectra of **CpDEDPA** upon Cu^{2+} addition (left) and UV-vis titration to endpoint to determine ligand concentration (right).

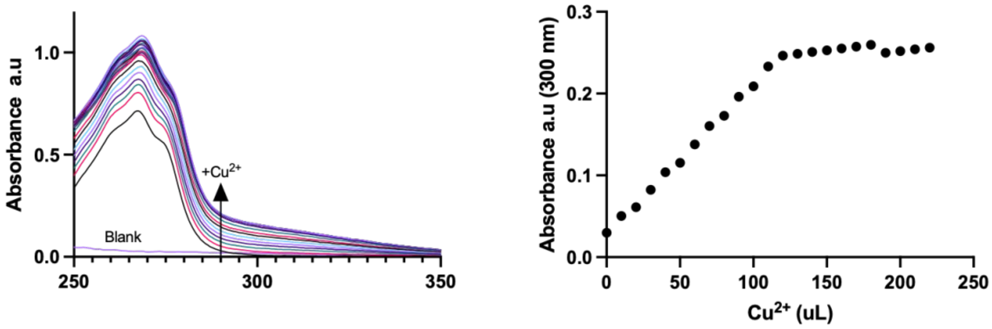

Figure S34. Spectrophotometric titration of **CBuDEDPA** with Cu^{2+}. UV-Vis absorbance spectra of **CBuDEDPA** upon Cu^{2+} addition (left) and UV-vis titration to endpoint to determine ligand concentration (right).

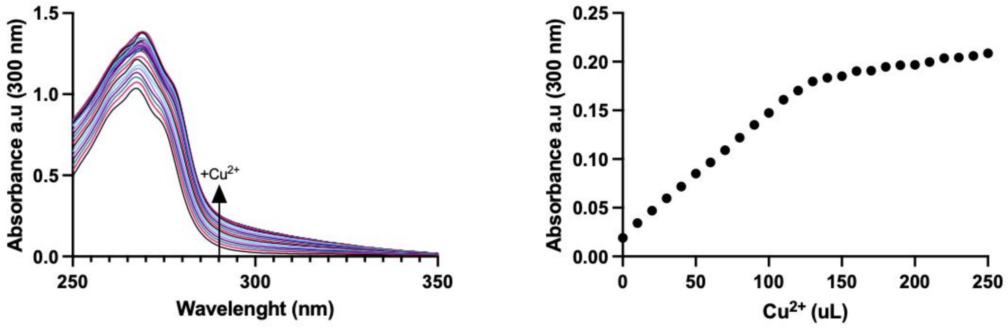

Figure S353. Spectrophotometric titration of **CBuDEDPA-NHBoc** with Cu^{2+}. UV-Vis absorbance spectra of **CBuDEDPA-NHBoc** upon Cu^{2+} addition (left) and UV-vis titration to endpoint to determine ligand concentration (right).

Table S6. Apparent Molar Activity (AMA) in mCi.µmol^{-1}.

	15 min	30 min	60 min
**CHXDEDPA**	631	610	670
**CpDEDPA**	565	647	682
**CBuDEDPA**	4939	6148	6637
**CBuDEDPA-NHBoc**	3583	3981	4245

Table S7. Percentage of the intact ^{64}Cu-complexes in PBS buffer.

	**CHXDEDPA**	**CpDEDPA**	**CBuDEDPA**	**CBuDEDPA-NHBoc**
0 min	96.73±0.64	97.57±0.15	97.90±0.02	95.33±0.21
1 min	97.47±0.55	96.83±0.21	97.53±0.55	94.73±0.11
2 min	97.73±0.06	98.10±0.87	97.53±0.55	95.03±0.21
3 min	98.03±0.21	96.73±0.29	97.83±0.06	95.23±0.12
12 h	98.70±0.10	97.63±0.15	97.53±0.55	95.47±0.12
24 h	96.93±0.55	96.80±0.26	97.53±0.55	95.47±0.12

Table S8. Percentage of the intact ^{64}Cu-complexes in DTPA solution.

	**CHXDEDPA**	**CpDEDPA**	**CBuDEDPA**
0 min	97.87±0.59	95.83±3.02	97.63±0.74
1 min	98.20±0.38	98.30±0.10	98.07±0.64
3 min	98.33±0.49	97.93±0.15	98.83±0.32
12 h	98.77±0.06	98.60±0.10	99.20±0.36
24 h	96.93±0.55	83.67±1.50	91.63±1.79

Table S9. Crystal data and structure refinement for **[Cu(CHXDEDPA)]·$(CH_3)_2CO\cdot H_2O$**, **[Cu(CpDEDPA)]·$4H_2O$** and **[Cu(CBuDEDPA)]**.

	[Cu(CHXDEDPA)]·$(CH_3)_2CO\cdot H_2O$	[Cu(CpDEDPA)]·$4H_2O$	[Cu(CBuDEDPA)]
Empirical formula	$C_{23}H_{30}CuN_4O_6$	$C_{19}H_{28}N_4O_8Cu$	$C_{18}H_{18}N_4O_4Cu$
Molecular weight MW	522.05	503.99	417.90
Crystal system	Orthorhombic	Monoclinic	Monoclinic
Space group	$P2_12_12_1$	P21/n	P21/c
a/Å	9.6799(2)	9.1524(7)	8.2649(2)
b/Å	12.5176(4)	13.3645(8)	11.6035(4)
c/Å	19.1379(6)	17.6647(13)	23.1196(7)
β/º		91.948(3)	90.9790(10)
Volume (Å^3)	2318.92(11)	2159.4(3)	2216.89(11)
Z	4	4	4
ρ_{calc} (g/cm^3)	1.495	1.550	1.252
μ (mm^{-1})	0.990	1.066	1.012
θ range	1.94º - 28.29º	2.47º - 28.27º	2.49º - 28.29º
R_{int}	0.0386	0.1163	0.0253
Measured reflections	43323	9154	60532
Independent reflections / unique (I >2σ (I)	5761 / 5373	9154 / 8019	5486 / 5232
Goodness-of-fit on F^2	1.037	1.109	1.110
Flack parameter	-0.008(4)		
R_1	0.0252	0.0459	0.0428
wR_2 (all data)	0.0649	0.1354	0.1139
Larg. diff. peak and hole (eÅ^{-3})	0.29/-0.52	0.50 and -0.76	0.47 and -0.46

References

(1) Chang, Z.; Boyaud, F.; Guillot, R.; Boddaert, T.; Aitken, D. J. A Photochemical Route to 3- and 4-Hydroxy Derivatives of 2-Aminocyclobutane-1-Carboxylic Acid with an All-Cis Geometry. *J. Org. Chem.* **2018**, *83* (1), 527–534. https://doi.org/10.1021/acs.joc.7b02559.

(2) Platas-Iglesias, C.; Mato-Iglesias, M.; Djanashvili, K.; Muller, R. N.; Elst, L. V.; Peters, J. A.; de Blas, A.; Rodríguez-Blas, T. Lanthanide Chelates Containing Pyridine Units with Potential Application as Contrast Agents in Magnetic Resonance Imaging. *Chem. Eur. J.* **2004**, *10* (14), 3579–3590. https://doi.org/10.1002/chem.200306031.
